# Assessment of developmental neurotoxicity induced by chemical mixtures using an adverse outcome pathway concept

**DOI:** 10.1186/s12940-020-00578-x

**Published:** 2020-02-24

**Authors:** Francesca Pistollato, Emilio Mendoza de Gyves, Donatella Carpi, Stephanie K. Bopp, Carolina Nunes, Andrew Worth, Anna Bal-Price

**Affiliations:** European Commission, Joint Research Centre (JRC), Ispra, Italy

**Keywords:** Mixture risk assessment, Adverse outcome pathway, Human induced pluripotent stem cells, Neuronal differentiation, Children health

## Abstract

**Background:**

In light of the vulnerability of the developing brain, mixture risk assessment (MRA) for the evaluation of developmental neurotoxicity (DNT) should be implemented, since infants and children are co-exposed to more than one chemical at a time. One possible approach to tackle MRA could be to cluster DNT chemicals in a mixture on the basis of their mode of action (MoA) into ‘similar’ and ‘dissimilar’, but still contributing to the same adverse outcome, and anchor DNT assays to common key events (CKEs) identified in DNT-specific adverse outcome pathways (AOPs). Moreover, the use of human in vitro models, such as induced pluripotent stem cell (hiPSC)-derived neuronal and glial cultures would enable mechanistic understanding of chemically-induced adverse effects, avoiding species extrapolation.

**Methods:**

HiPSC-derived neural progenitors differentiated into mixed cultures of neurons and astrocytes were used to assess the effects of acute (3 days) and repeated dose (14 days) treatments with single chemicals and in mixtures belonging to different classes (i.e., lead(II) chloride and methylmercury chloride (heavy metals), chlorpyrifos (pesticide), bisphenol A (organic compound and endocrine disrupter), valproic acid (drug), and PCB138 (persistent organic pollutant and endocrine disrupter), which are associated with cognitive deficits, including learning and memory impairment in children. Selected chemicals were grouped based on their mode of action (MoA) into ‘similar’ and ‘dissimilar’ MoA compounds and their effects on synaptogenesis, neurite outgrowth, and brain derived neurotrophic factor (BDNF) protein levels, identified as CKEs in currently available AOPs relevant to DNT, were evaluated by immunocytochemistry and high content imaging analysis.

**Results:**

Chemicals working through similar MoA (i.e., alterations of BDNF levels), at non-cytotoxic (IC_20_/100), very low toxic (IC_5_), or moderately toxic (IC_20_) concentrations, induce DNT effects in mixtures, as shown by increased number of neurons, impairment of neurite outgrowth and synaptogenesis (the most sensitive endpoint as confirmed by mathematical modelling) and increase of BDNF levels, to a certain extent reproducing autism-like cellular changes observed in the brain of autistic children.

**Conclusions:**

Our findings suggest that the use of human iPSC-derived mixed neuronal/glial cultures applied to a battery of assays anchored to key events of an AOP network represents a valuable approach to identify mixtures of chemicals with potential to cause learning and memory impairment in children.

## Background

Chemicals that are known to cause developmental neurotoxicity (DNT) belong to different classes, such as organic solvents, metals, or use categories, such as industrial chemicals, pesticides, endocrine disrupters (EDs), drugs or cosmetics. Approximately 218 chemicals are identified as neurotoxicants, of which 27 are metals or inorganic compounds, 41 are organic solvents, 48 are other organic substances and 102 are pesticides [1]. In a study by Maffini and Neltner [2], more than 300 compounds were identified as potential DNT chemicals. These chemicals belong also to various regulatory silos including food contaminants, food contact materials and food additives, such as flavourings, colourings and preservatives. The examples above illustrate that common, similar or related toxic effects triggered by various chemicals are regulated under separate pieces of legislation, and that combined effects of chemicals across different regulatory domains are possible, but not currently considered [3]. At the same time, it is well documented that “mixture effects” can be greater than the effects triggered by the most potent single chemical in a mixture, due to their additive or, in some cases, even synergistic effects. Taking into consideration the vulnerability of the developing brain, mixture risk assessment (MRA) for DNT effects should be implemented, since humans, including the unborn, infants and children are indisputably co-exposed to more than one chemical at a time [3]. For instance, breast milk [4] and cord blood [5] have been found to contain chemicals regulated as pesticides, along with those regulated as cosmetics (including UV filters, parabens, phthalates), and POPs, including polychlorinated biphenyls (PCBs), confirming that simultaneous co-exposure to multiple chemicals generally occurs during pregnancy, in new-borns and toddlers.

Since DNT chemicals may work through several mechanisms simultaneously, understanding the individual chemical contribution to a mixture effect is complex and makes MRA challenging. One possible approach could be to cluster DNT chemicals in a mixture on the basis of their mode of action (MoA) into ‘similar’ and ‘dissimilar’, but still contributing to the same adverse outcome (AO). This approach was recommended in the 2013 “Scientific Opinion” of EFSA Plant Protection Products and their Residues (PPR) Panel on the relevance of dissimilar mode of action (MoA) for pesticides residues in food” [6], supported by the more recent general guidance on risk assessment of combined exposure to multiple chemicals [7].

In this study, in line with the EFSA Scientific Opinions, selected chemicals were clustered into two categories: (i) similar MoA: chemicals working at least through one common MoA; in our case alterations of BDNF levels leading to (or associated with) alterations of synaptogenesis (Lead(II) chloride, a heavy metal; Chlorpyrifos, a pesticide; Bisphenol A, an organic synthetic compound and ED), and (ii) dissimilar MoAs, working through multiple mechanisms, but not directly linked to alterations of BDNF levels (Methylmercury, a heavy metal; Valproic acid, a drug; PCB138, a persistent organic pollutant (POP) and ED). Finding chemicals belonging to different classes and working through at least one common mechanism (in our case, alterations of BDNF levels, defined as KE of DNT AOPs) has been performed based on literature reviewing (Additional file 1: Tables S1 and S2). The applied concentrations of studied chemicals were carefully selected taking into consideration the concentrations of each chemical found in human samples, such as cord blood, mother’s or children’s blood, breast milk or other samples (Additional file 1: Tables S1 and S2).

Additionally, it should be acknowledged that environmental chemicals causing DNT often elicit multiple direct and indirect effects, which may also vary depending on the dose, the brain developmental stage, the duration of exposure, and interactions with other environmental factors. Hence, the categorisation of chemicals into ‘similar’ and ‘dissimilar’ MoA(s), as recommended by the EFSA PPR Panel [6, 7], while enabling the assessment of dose addition, may present some limitations when applied to ‘dirty’ environmental neurotoxicants. Moreover, the EFSA PPR Panel recommended using MRA methods based on dose addition (DA) not only for chemicals that act through similar MoA, but also for the assessment of mixtures of pesticides with dissimilar MoA, provided they produce a common AO. Indeed, DA is regarded as being sufficiently conservative to serve as a default concept also for the evaluation of mixtures of dissimilarly acting chemicals. Consequently, one unifying approach was proposed by the authors of the EFSA Opinion [6] for dealing with mixtures in regulatory practice, irrespective of MoA.

In this regard, it has been proven that certain EDs show dose-additivity even if they do not share the same primary molecular target [8]. Since then, further scientific evidence has corroborated the relevance of addressing also combined effects from dissimilarly acting chemicals in MRA, in particular for chemicals having effects on (or acting via) the endocrine system [6].

**Fig. 1:**
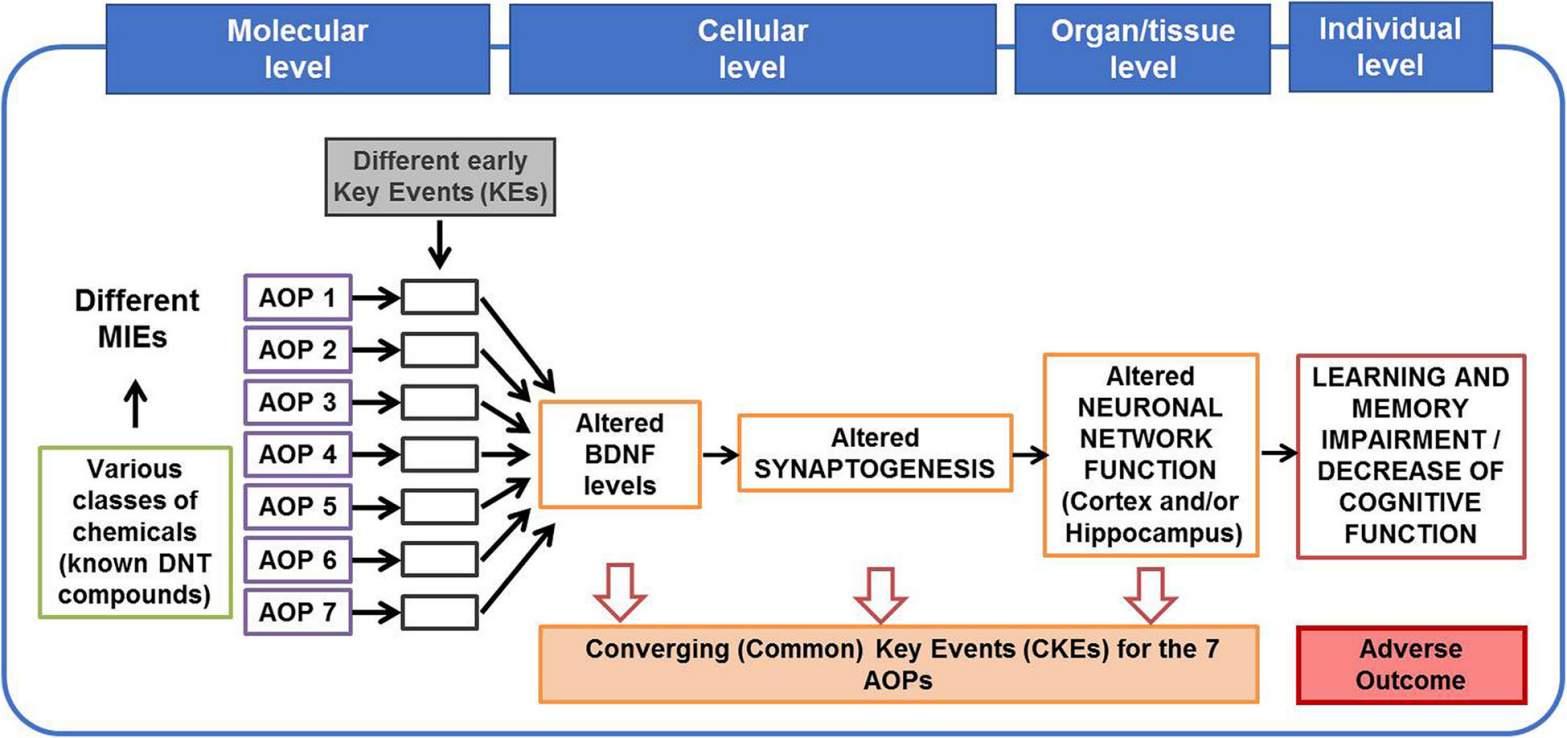
Seven DNT AOPs with multiple MIEs leading to the similar AO. Alteration of brain derived neurotrophic factor (BDNF) levels, alteration of synaptogenesis and alterations of neuronal network functions) are defined as KEs common to majority of these seven AOPs, which all lead to the adverse outcome (AO) defined as learning and memory impairment or cognitive function deficit

To date, seven DNT AOPs have been developed [9–15] or are still under finalisation [14, 16] in which cognitive damage, including learning and memory impairment in children has been identified as an AO. These AOPs are triggered by different molecular initiating events (MIEs) and various early key events (KEs), but three KEs before the AO are common KEs (CKEs) for most of them: (i) altered brain derived neurotrophic factor (BDNF) levels; (ii) altered synaptogenesis, and (iii) altered neuronal network function, as summarised in Fig. 1.

These CKEs, linked in a causal manner, as described by key event relationships (KERs) in the AOPs, are essential for inducing learning and memory impairment. The BDNF-ERK-CREB (extracellular signal-regulated kinase / cyclic AMP response element-binding protein) signalling cascade (KE upstream) plays a critical role during brain development including neuronal survival, differentiation (dendrite and neurite formation), synaptogenesis and neuronal network formation [17, 18]. Therefore, any change in the BDNF level (increase or decrease) could result in alterations of synaptogenesis, leading to neuronal network dysfunction, as described in the KERs of the AOP ID 12 [13], AOP ID 13 [12], or AOP ID 54 [9], and strongly supported by empirical data [19–28].

In this study we used in vitro assays anchored to the CKEs described in these AOPs [29–32] to determine whether mechanistic knowledge described in the AOP network (Fig. 1) could serve as a frame for DNT testing, facilitating data interpretation and their possible application for regulatory purposes.

The battery of the in vitro assays was applied to hiPSC-derived neural stem cells (NSCs) differentiated into a mixed culture of neurons and astrocytes, since this model recapitulates, most of the key processes critical and specific for human brain development including neural progenitor cell commitment, proliferation, migration, neuronal and glial differentiation, synaptogenesis, and neuronal network formation and function [33–35]. The readiness of these in vitro methods for regulatory purposes has been recently evaluated based on 13 established semi-quantitative criteria [36]. It is postulated that if a chemical at the concentration relevant to environmental exposure affects at least one of these key neurodevelopmental processes in a statistically significant manner it should be defined as potential developmental neurotoxicant [37].

These key neurodevelopmental processes can be quantitatively assessed upon exposure (acute or chronic) to a single chemical or a mixture.

Taking into consideration real life exposure, we have reconstructed mixtures of chemicals following five main criteria: (i) presence of chemicals in human samples, (ii) belonging to different classes (e.g., pesticides, industrial chemicals, heavy metals, polychlorinated biphenyls, EDs, and drugs), (iii) acting through common KEs identified in the AOP network, (iv) working through similar and dissimilar MoAs, according to the EFSA definition [6], and (v) associated with cognitive impairment (AO) in children. Selected chemicals were used as a proof-of-concept to verify whether mixtures of these chemicals affect BDNF levels, neuronal differentiation and synaptogenesis, as postulated in the AOPs. The overall aim of this study was to determine, by following an AOP network-driven testing strategy, whether non-neurotoxic concentrations of single chemicals will produce DNT effects in mixtures.

## Methods

### Human induced pluripotent stem cell (hiPSC)-derived neural stem cells (NSCs) differentiated into mixed culture of neurons and astrocytes

Neural stem cells (NSCs), originally derived from IMR90-hiPSCs (kindly provided by Prof Marc Peschanski, I-Stem, France), were used to obtain differentiated neurons in mixed neuronal/astrocytic culture. Further information regarding the test system characterisation and detailed procedures on how to culture and differentiate these cells can be found in [38]. In brief, NSCs obtained from neuroectodermal derivatives (rosettes) were passaged, plated onto reduced growth factor matrigel-coated 96 well plates (pre-coated with poly-D-lysine) at a density of 7000 cells/well (i.e., 21.000 cells/cm^2^), and differentiated for either 21 or 28 days in vitro (DIV). At 21 DIV a mixed population of neurons (35–42% glutamatergic neurons, 15–20% GABAergic neurons, 13–20% dopaminergic neurons) and astrocytes (15–20%) was obtained [38, 39].

### Exposure to single and mixed chemicals

**Fig. 2:**
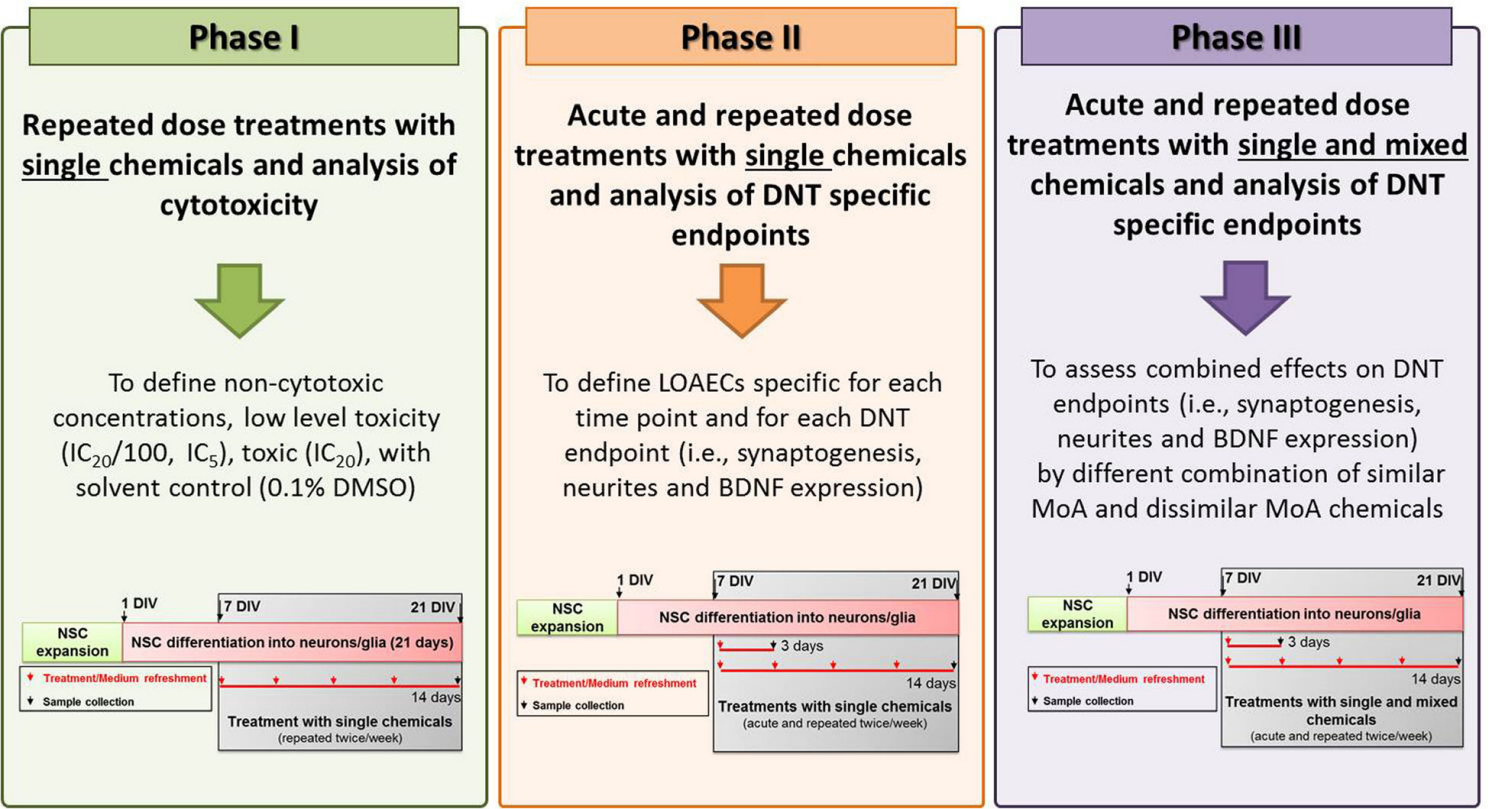
The three experimental phases. Phase 1 aimed at defining non-cytotoxic and very low toxic (IC_20_/100, IC_5_), and moderately toxic (IC_20_) concentrations, compared to solvent control (0.1% DMSO) at the respective time point for each chemical. Goal of Phase 2 was to define for each individual chemical, for each time point (i.e., 3 days and 14 days) and for each analysed DNT endpoint (i.e., synaptogenesis, neurite outgrowth and BDNF levels), the Lowest Observable Adverse Effect Concentrations (i.e., LOAEC-syn, LOAEC-neu and LOAEC-bdnf), based on analysis of statistical significance. During this phase, treatments with single chemicals at the concentrations defined in Phase 1 (i.e., non-cytotoxic (IC_20_/100), very low toxic (IC_5_) and moderately toxic (IC_20_)) were performed to assess their effects on synaptogenesis, neurite outgrowth and BDNF levels. During Phase 3, treatments with single chemicals and mixtures at the LOAECs concentrations defined at the end of Phase 2 were performed to assess possible combined effects, assessing the same DNT-specific endpoints as described in Phase 2

After 7 days of differentiation (7 DIV), NSCs were treated with Lead(II) chloride (Lead) (Sigma, 200 mM stock solution in DMSO), Chlorpyrifos (CPF) (Sigma, 500 mM stock solution in DMSO), Bisphenol A (BPA) (Sigma, 400 mM stock solution in DMSO), Methylmercury(II) chloride (Methyl-Hg) (Sigma, 10 mM stock solution in DMSO), Valproic acid sodium salt (VA) (Sigma, 1000 mM stock solution in purified milliQ water), PCB 138 (Sigma, 100 mM stock solution in DMSO) administered as single chemicals or in mixtures (i.e., combining BPA, CPF and lead; similar MoA chemicals (Additional file 1: Table S1), or Methyl-Hg, PCB138, VA; dissimilar MoA chemicals (Additional file 1: Table S2), or all six chemicals together (All; see Phase 3 below)), for either 3 days (acute treatments) or 14 days (repeated dose treatments, refreshing medium containing chemical(s) twice/week). The experimental approach was divided into three phases, as summarised in Fig. 2.

In Phase 1, dose-response curves for cytotoxicity of the individual chemicals were assessed after 14 day treatment for identifying non-cytotoxic (IC_20_/100), very low cytotoxic (IC_5_) and moderately toxic (IC_20_) concentrations, compared with solvent in control cultures at the respective time point (0.1% DMSO) for each chemical (all from Sigma). CellTiter-Blue® Reagent was used to measure cytotoxicity as described by the manufacturer.

In Phase 2, treatments with single chemicals at non-cytotoxic, very low cytotoxic or moderately toxic concentrations defined in Phase 1 (IC_20_/100, IC_5_ and IC_20_ respectively) were performed to assess their effects on synaptogenesis, neurite outgrowth and BDNF protein levels, in vitro assays anchored to KEs defined in DNT relevant AOPs and assessed by quantitative immunocytochemistry using high content imaging (HCI) analysis (Cellomics). Goal of Phase 2 was to define for each individual chemical the Lowest Observable Adverse Effect Concentration (LOAEC) specific for each time interval (3 days and 14 days) and for each analysed DNT endpoint, i.e., LOAEC-syn (for synaptogenesis), LOAEC-neu (for neurite outgrowth) and LOAEC-bdnf (for BDNF protein levels). These LOAECs were calculated based on analysis of statistical significance (detailed below), comparing effects induced by the three selected chemical concentrations (IC_20_/100, IC_5_ and IC_20_) vs solvent control culture (0.1% DMSO) at the respective time point, and were used to assess the effects of mixtures on DNT-specific endpoints during Phase 3.

In Phase 3, treatments with single chemicals and different chemical mixtures at the LOAEC-syn, LOAEC-neu and LOAEC-bdnf defined at the end of Phase 2 were performed to assess possible combined effects and mixture-specific LOAECs for each DNT specific endpoint, compared to solvent control culture (0.1% DMSO) at the respective time point. Three different categories of mixtures were created: (i) a mixture containing 3 chemicals with similar MoA (3-sim); (ii) a mixture containing 3 chemicals with dissimilar MoAs (3-dissim), and (iii) a mixture containing all 6 chemicals together (All). LOAECs of chemicals used to prepare the mixtures differed depending on the sensitivity of DNT endpoints and the time of treatment. At the beginning of Phase 3, cell viability analysis was performed again to account for possible cytotoxic effects elicited by chemical mixtures. Based on these results, if high toxicity was observed, mixtures were further diluted by applying a 2 dilution factor to mixture-specific LOAECs for each DNT endpoint (3-Sim, 3-Dissim and All).

### Analysis of cell viability with CellTiter-blue®

IMR90-NSCs undergoing differentiation were exposed to different concentrations of chemicals for 14 days to determine a cytotoxic curve and define non-cytotoxic, very low or moderately toxic concentrations (Phase 1), and a second time to determine possible cytotoxic effects elicited by mixtures after either 3 or 14 day (Phase 3). Briefly, at the end of treatment period, cells were incubated with CellTiter-Blue® Reagent (at a 1:6 dilution, i.e., 30 μL were added to 150 μL medium per well) in the incubator (37 °C, 5% CO_2_) for 3–4 h. Resazurin is the active ingredient of CellTiter-Blue® Reagent, which upon entering live cells gets converted to resorufin that is red and highly fluorescent, and its absorbance has been read on a spectrophotometer. After the incubation, 100 μL medium/reagent were transferred into new plates, and fluorescence was measured at 530–560 nm/590 nm (excitation/emission) in a multiwell fluorimetric reader (Tecan). The results were normalised to the mean of solvent treated cells (0.1% DMSO).

### Quantitative immunocytochemistry (IC) using high content imaging (HCI) analysis

After 3 days and 14 days, cells were fixed with 4% formaldehyde, washed twice with PBS 1X (w/o calcium and magnesium), and stored in PBS 1X at 4 °C prior to use. When ready for staining, cells were permeabilised in PBS 1X containing 0.1% Triton-X-100 and 3.5% bovine serum albumin (BSA) for 15 min at room temperature, and further incubated with 3.5% BSA in 1X PBS (blocking solution) to prevent nonspecific binding of the antibodies. For the analysis of synaptogenesis, cells were stained with microtubule-associated protein-2 (MAP2, chicken, 1:3000, Abcam), synaptophysin (pre-synaptic marker) (SYP, rabbit, 1:300, Abcam), and post-synaptic density protein 95 (PSD95, mouse, 1:300, Abcam) specific antibodies. Additionally, analysis of neurite outgrowth (by β-III-tubulin (mouse, 1:500, Thermofisher) staining) and BDNF (rabbit, 1:70, Thermofisher) levels was performed. Cells were also stained for: neurofilament 200 (NF200, rabbit, 1:1000, Sigma-Aldrich), glial fibrillary acidic protein (GFAP, mouse, 1:500, Merck-Millipore), and nestin (rabbit, 1:200, Sigma-Aldrich). All primary antibodies were diluted in blocking solution and incubated overnight at 4 °C. Cells were washed twice with PBS 1X and further incubated for 45 min with fluorochrome-conjugated secondary antibodies (1:500, all Abcam), and nuclei counterstained with 1 μg/mL DAPI (Thermofisher). Quantification of mean fluorescence intensity and of the relative percentages of cell types was performed using the ArrayScan algorithm ‘Neuronal Profiling V4.1’ bioapplication, which applies a specific nuclear mask around the DAPI staining defined according to nuclear morphology, discarding invalid nuclei (i.e., pyknotic and bright nuclei) and, on the valid nuclei (i.e., homogenous round-shaped nuclei, indicative of live cells) an additional cell body mask was applied according to the type of antibody/antigen staining, as already described [40]. Other masks were used to respectively identify neurites and the fluorescence intensity levels of SYP, PSD95 and BDNF proteins. Secondary antibody incubation alone was used to determine the intensity level of fluorescent background. The ArrayScan™ XTI High Content Platform (Cellomics) was set up to take a minimum of 12–16 pictures/well at 10x magnification. A total of 6 to 8 internal replicates for each condition were performed. For qualitative analysis, 20x and 40x magnification pictures were also taken.

### Bench mark dose Modelling

Parametrical dose response analysis was applied to the observed perturbation of each DNT specific endpoint after exposure to single chemicals for 14 days. The fitting curves were computed for seven different mathematical models (i.e., Hill, Power, Linear, Polynomial 2, Exponential 2, Exponential 3, Exponential 4, and Exponential 5) by using the BMDExpress.2 open access software (https://github.com/auerbachs/BMDExpress-2/wiki). The best-fit model, i.e., lowest Akaike information criterion and higher fit *P* value, was selected for each chemical and endpoint, allowing retrieving the Bench Mark Dose (BMD) associated with a 5% change of response (BMD_5_). The upper (BMDU) and the lower (BMDL) bounds were also calculated to estimate the uncertainty of the BMD_5_ (Additional file 4 _Figure S10_Tables S3-S4-S5). Notably, the non-monotonic dose response curves for each chemical and specific for each DNT endpoint followed different trends and shapes, preventing the application of the model to the response of the mixture, as described in the 2016 EFSA Guidance on ‘The use of the benchmark dose approach in risk assessment’ [41]. Therefore, to evaluate the potency of the individual chemicals in the mixtures, we calculated, for each DNT endpoint, the Bench Mark Response (BMR) of single chemicals considering the concentrations used in the mixtures, according to the best fit model calculated in the Parametrical dose response analysis. The single chemical BMR values were compared with the measured mixture effects (normalized to untreated control). Moreover, the concentration addition approach and the Toxic Unit (TU) model [42] were applied, considering, for three chemicals, the following formula:

$$ \mathrm{TU}=\left[\mathrm{chem}1\right]/{\mathrm{BMD}}_{5\left(\mathrm{chem}1\right)}+\left[\mathrm{chem}2\right]/{\mathrm{BMD}}_{5\left(\mathrm{chem}2\right)}+\left[\mathrm{chem}3\right]/{\mathrm{BMD}}_{5\left(\mathrm{chem}3\right)}. $$

According to this approach, when TU ≤ 1, the predicted additive response caused by the mixture is lower than 5%, whilst if TU > 1, mixture effects cannot be predicted by this model.

### Statistical analysis

Statistical significance was assessed by one-way ANOVA with Dunnett’s Multiple Comparison Test as Post Test, comparing all conditions vs solvent control (Ctr, 0.1% DMSO) (or vs NSCs, undifferentiated cells) using the GraphPad Prism 5 software (http://www.graphpad.com/). All data represent the average of at least 3 biological replicates ± standard error mean (S.E.M.). For all graphs, an asterisk over a data point indicates a significant difference with the control group. For all graphs, * *p* < 0.05, ** *p* < 0.01, *** *p* < 0.001.

## Results

### Criteria for chemical selection

Based on human epidemiological data and presence of chemicals in human samples (e.g., [4, 5]), chemicals that are linked to cognitive deficit in children, including learning and memory impairment (AO of the DNT AOPs) were identified and divided into two groups according to the KEs of the relevant AOPs: those were (1) impairment of BDNF synthesis and release associated with alterations of synaptogenesis (similar MoA), and (2) alteration of synaptogenesis through multiple mechanisms, not directly linked to changes of BDNF levels (dissimilar MoA).

Summing up, the following five criteria were applied for chemical selection:

1. Chemicals associated with cognitive/learning and memory impairment in children (epidemiological studies)
2. Chemicals acting through identified CKEs in the DNT AOPs
3. Chemicals representing different classes (i.e., pesticides, industrial chemicals, heavy metals, POPs, EDs, and drugs)
4. Chemicals found in human samples (e.g., breast milk, cord blood, urine, hair, umbilical cord plasma, brain tissues, maternal blood, or children’s blood)
5. Chemicals working through similar and dissimilar MoAs (as described above), according to the EFSA definition [6].

Additional file 1: Tables S1 and S2 summarise the chemicals that have been selected according to the criteria outlined above. In particular, Lead(II) chloride (Lead), Chlorpyrifos (CPF), Bisphenol A (BPA), Methylmercury(II) chloride (Methyl-Hg), PCB138, and Valproic acid (VA) were prioritised for their: (i) proven effects on synaptogenesis alteration (CKE), (ii) involvement in cognitive impairment (AO), (iii) documented effects in epidemiological studies, (iv) known MoA, categorised as similar (BPA, CPF and lead; Additional file 1: Table S1), and dissimilar (Methyl-Hg, PCB138, VA, Additional file 1: Table S2) based on the established criteria.

The effects of these six chemicals (administered individually or in mixtures) were assessed on hiPSC-derived NSCs undergoing differentiation towards neurons and astrocyte-like cells after 3 or 14 days of exposure.

### Characterisation of synaptogenesis, neurite outgrowth and BDNF protein levels in hiPSC-derived NSCs undergoing differentiation in the control culture

**Fig. 3:**
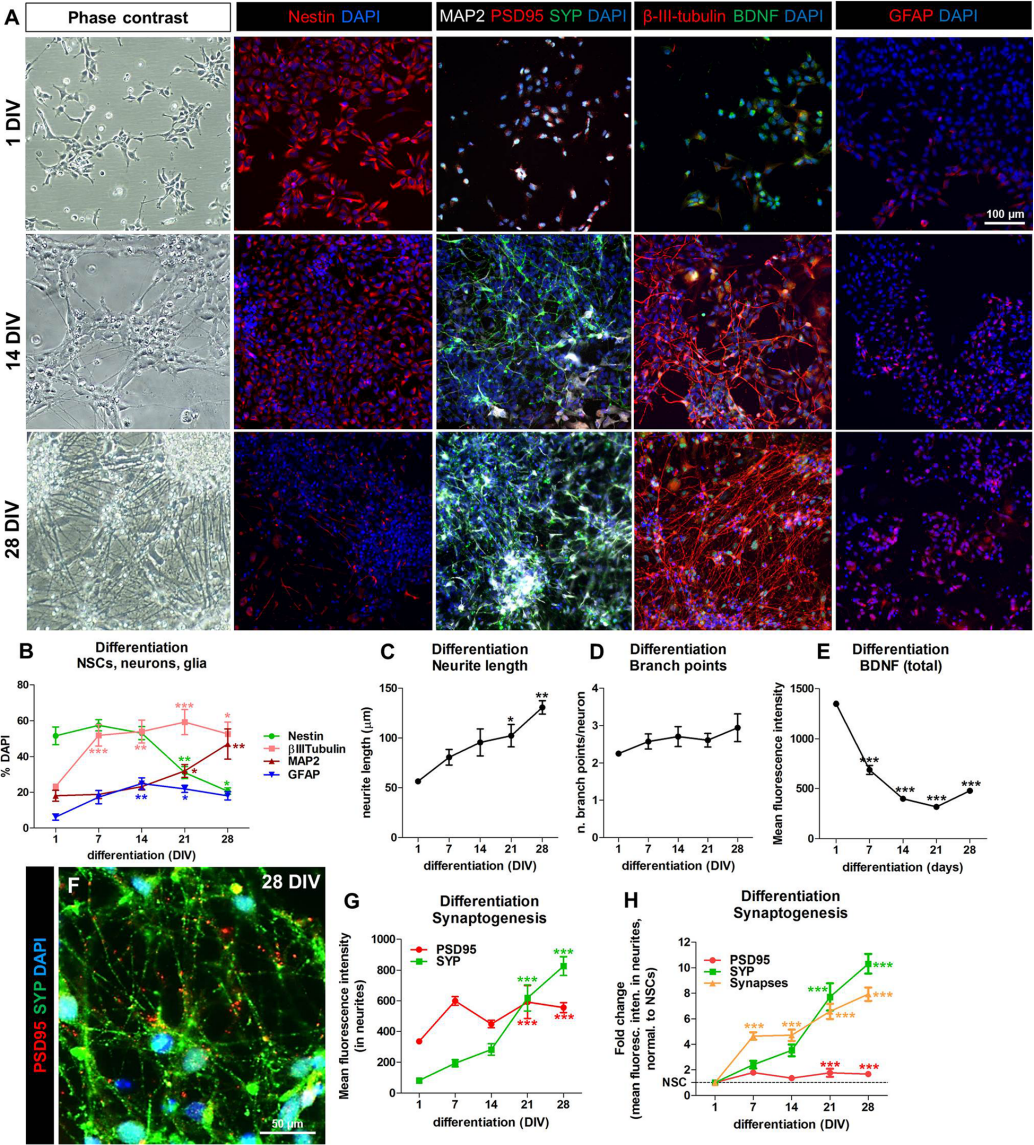
Characterisation of synaptogenesis, neurite outgrowth and BDNF protein levels in hiPSC-NSCs undergoing differentiation toward neurons and astrocytes. (**a**) Representative phase contrast and immunocytochemical (IC) images of NSCs (1 DIV, upper row), NSCs following 14 days (14 DIV, middle row) and 28 days of differentiation (28 DIV, lower row). Immunocytochemical images display cells stained for nestin (red), microtubule-associated protein-2 (MAP2, white) with postsynaptic density protein 95 (PSD95, red) and synaptophysin (SYP, green), β-III-tubulin (red) and brain derived neurotrophic factor (BDNF, green), and glial fibrillary acidic protein (GFAP, red). (**b**) Quantification of nestin, β-III-tubulin, MAP2 and GFAP expressing cells shown as percentage of DAPI stained cells, comparing cells at 7, 14, 21 and 28 DIV of differentiation, to NSCs (1 DIV). Analysis was performed using immunofluorescence and high content imaging (HCI), using the Array Scan vTi platform and the Neuronal profiling V4.1 BioApplication. (**c**) Neurite length analysis and (**d**) number of branch points/neuron were evaluated by using β-III-tubulin staining. (**e**) Quantification of the total BDNF levels (mean average intensity normalised to undifferentiated NSCs, 1 DIV). (**f**) Representative immunocytochemical image (at 40x magnification) of NSCs differentiated for 28 DIV and stained for PSD95 (red) and SYP (green). (**g**, **h**) Total levels (**g**) and normalised levels (**h**) of PSD95 and SYP proteins expressed as mean fluorescence intensity localised in neurites (stained with MAP2, not shown in the picture). In **h**, values were relative to undifferentiated NSCs; in H also the number of synapses (i.e., number of overlapping SYP and PSD95 spots in the neurites) is shown. Data are represented as mean ± S.E.M. of 3–4 biological replicates

Synaptogenesis was determined based on the co-localised expression of pre- and post-synaptic proteins (i.e., SYP and PSD95 respectively, along with the dendritic marker MAP2) following a Thermo-Fisher standardised protocol (https://www.thermofisher.com/it/en/home/life-science/cell-analysis/cellular-imaging/high-content-screening/hcs-applications/hcs-synaptogenesis-assay.html). In the same culture, neurite length (assessed in cells stained for β-III-tubulin) and BDNF protein levels in IMR90-NSCs undergoing differentiation were also characterised. Data showed that the expression of the neuronal markers β-III-tubulin and MAP2 increased over time, which is indicative of neuronal differentiation. Additionally, about 15–20% of cells expressing glial fibrillary acidic protein (GFAP) was present (indicative of astrocytes), along with about 20–30% of cells retaining nestin expression (NSCs) (Fig. 3a, b) at 21 DIV. The length of neurites and the number of branch points/neurite analysed by β-III-tubulin staining, progressively increased during differentiation (Fig. 3a, c and d) (up to 28 DIV). Moreover, the levels of both SYP (presynaptic marker) and PSD95 (post-synaptic marker) did also increase over time by about 7 and 1.7 fold respectively (at 21 DIV) especially at the level of dendrites (stained with MAP2), along with the number of synapses (i.e., number of overlapping SYP/PSD95 spots in the neurites) (~ 6.5 fold at 21 DIV) (Fig. 3a, f-h). Moreover, we found that BDNF protein levels were particularly high in proliferating NSCs, and decreased during differentiation (Fig. 3a, e).

### Cytotoxicity analysis of single chemicals (phase 1)

We analysed cytotoxicity elicited by the six single chemicals with the aim to define non-cytotoxic (IC_20_/100), very low (IC_5_) and moderately toxic (IC_20_) concentrations. HiPSC-derived NSCs were differentiated for 7 days; starting from 7 DIV, cells were exposed for 14 days to single chemicals, refreshing medium and chemical treatments twice/week. Comparative analysis with media containing solvent (0.1% DMSO) was performed for each chemical. Table 1 summarises the individual chemical concentrations tested in Phase 1, along with chemical concentrations found in human samples (i.e., blood and cord blood) for comparative purposes.

**Table 1:** Chemical concentrations tested in vitro (Phase 1) in relation to concentrations found in human samples

Chemical	Abbreviation	Concentrations tested in vitro	Concentrations found in human samples
Lead(II) chloride	Lead	200, 50, 12.50, 3.13, 0.78, 0.20 μM	Cord blood: range 1.09–11.41 μg/L ➔0.0039–0.041 μM Children blood: Range 1.71–10 μg/dL ➔0.061–0.36 μM IPChem: Blood-whole blood: 3.76–69, 3.42–28.8, 4.13–43.6, 6.05–23.1 μg/L (range 3.42–69) ➔ 0.012–0.25 μM Cord blood-whole blood (considering for plasma, 1.025 g/mL) 2.68–36.4 ng/g ➔ 0.00988–0.13 μM
Chlorpyrifos	CPF	500, 125, 31.25, 7.81, 1.95, 0.49 μM	Cord plasma: 4.65 ng/mL ➔ 0.013 μM Cord blood: Range 2.5–6.17 pg/g plasma (considering for plasma, 1.025 g/mL) ➔ 7.3 × 10^− 6^ - 1.8 × 10^− 5^ μM
Bisphenol A	BPA	400, 100, 25, 6.25, 1.56, 0.39, 0.10 μM	Children serum: Range 0.85–22.5 ng/mL ➔ 0.0037–0.098 μM IPChem: Blood – plasma: n.d.- 3.5 ng/g (considering for plasma, 1.025 g/mL) ➔ n.d. - 0.016 μM Cord blood-whole blood: n.d.-1.9 ng/g (considering for plasma, 1.025 g/mL) ➔ n.d. - 0.0085 μM
Methyl-mercury(II) chloride	Methyl-Hg	10, 2.50, 0.63, 0.16, 0.04, 0.01, 0.0024, 0.0006 μM	Cord blood: range 0.70–35 μg/L ➔ 0.0028–0.14 μM Children blood: Range 1.46–6.81 μg/L ➔ 0.0058–0.027 μM IPChem: Blood-whole blood: 0.11–10.2, 0.002–4.17, 0.19–7.93, 0.13–5.95 μg/L (range 0.002–10.2) ➔ 8 × 10^− 6^ - 0.041 μM Cord blood-whole blood: 0.16–14.1 ng/g (considering for plasma, 1.025 g/mL) ➔0.00065–0.058 μM Cord blood-whole blood: n.d. - 8.4 μg/L ➔ n.d.-0.033 μM Blood –plasma: n.d. - 4.2 μg/L ➔ n.d.- 0.017 μM
Valproic acid	VA	10.000, 2500, 625, 156, 39, 10 μM	Cord blood: Range 3.87–75 μg/ml ➔ 26.8–520 μM
PCB138	PCB138	100, 25, 6.25, 1.56, 0.39, 0.10, 0.02 μM	Cord plasma: Range 0.14–0.18 ng/mL ➔ 3.87 × 10^− 4^ - 5 × 10^− 4^ μM IPChem: cord plasma: 270–460 ng/L ➔ 0.00075–0.0013 μM

**Fig. 4:**
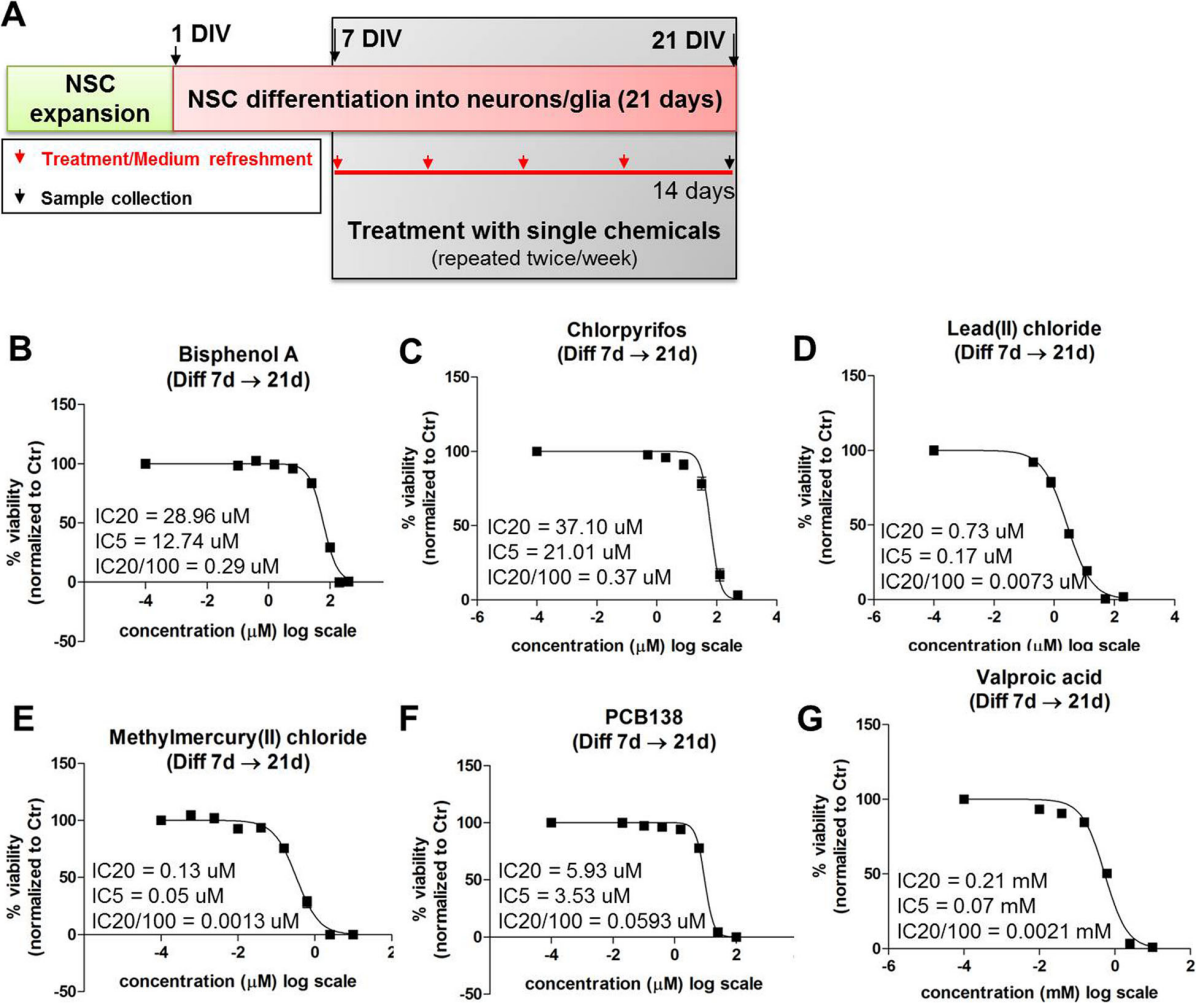
Analysis of cell viability upon treatments with single chemicals in hiPSC-derived NSCs undergoing differentiation. (**a**) hiPSC-derived NSCs were differentiated for 7 DIV, followed by 14 days treatment with different concentrations of individual chemicals. After 14 days (i.e., 21 DIV), resazurin test was performed (**b**-**g**). All samples were normalised to solvent control (0.1% DMSO, Ctr) at the respective time point. All chemicals were tested in 3 to 5 experimental replicates, considering 6 internal replicates for each concentration. After measurement, the normalised values were imported into GraphPad Prism, where a non-linear fit (sigmoidal dose-response (variable slope)) was performed in order to calculate the inhibitory concentration (IC) values reported in (**b**-**g**)

Based on cytotoxicity curve analysis, IC_20_/100, IC_5_ and IC_20_ were defined (Fig. 4). Those concentrations were then used to assess single chemical effects on selected DNT-specific endpoints (Phase 2).

### Effects of single chemicals on selected DNT-specific endpoints (phase 2)

During Phase 2 we defined, for each chemical, the LOAECs specific for each time point (i.e., 3d and 14d treatment) and for each DNT endpoint (i.e., synaptogenesis, neurite outgrowth and BDNF levels), permitting the definition of LOAEC-syn, LOAEC-neu and LOAEC-bdnf. To this aim, cells were treated with individual chemicals at the IC_20_/100, IC_5_ and IC_20_ concentrations defined at the end of Phase 1 (Fig. 4b-g). Treatments and medium were refreshed twice a week; after 3 days and 14 days, cells were fixed and stained with antibodies specific for the analysis of synaptogenesis (i.e., SYP (pre-synaptic marker) co-localised with PSD95 (post-synaptic marker), dendrites (MAP2), neurite outgrowth (i.e., β-III-tubulin) and BDNF. Quantitative analysis of protein levels and distribution was assessed by HCI, using the ArrayScan XTI High Content Imaging Platform (Cellomics) analysis. The main effects elicited on studied DNT endpoints by individual chemicals are described in the following sections, illustrated in Additional file 2: Figure S1 to S6, and summarised in Table 2. It should be considered that any statistically significant variation, i.e., an increase or a decrease of the assessed neurodevelopmental features (synaptogenesis, neurite outgrowth and BDNF levels) compared to solvent control at the respective time point, might be indicative of a potential DNT effect.

**Table 2:** Summary of the main effects induced by single chemicals administered at IC_20_, IC_5_ and IC_20_/100. In bracket, the lowest concentration eliciting a statistically significant modification of at least one of the measured DNT features is indicated (Phase 2)

3 days	BPA	CPF	Lead	Methyl-Hg	PCB138	VA
Synaptogenesis (SYP, PSD95)	⇓ PSD95t (IC_20_)	⇑ SYPt (IC_20_)	⇓ PSD95n (IC_20_/100)	⇔	⇑ PSD95n (IC_20_)	⇔
Neurite outgrowth	⇔⇑ n. neurites (IC_5_)	⇓ ⇔	⇔	⇔	⇑ (IC_20_/100)	⇑ n. neurites (not shown) (IC_5_)
BDNF levels	⇔	⇑ BDNFt (IC_20_)	⇔	⇔	⇔ ⇑ ratio (IC_20_/100)	⇔
14 days	BPA	CPF	Lead	Methyl-Hg	PCB138	VA
Synaptogenesis (SYP, PSD95)	⇓ PSD95n (IC_5_)	⇓ SYPn ⇑ PSD95t (IC_5_)	⇑ SYPt ⇓ PSD95n (IC_20_/100)	⇓ SYPn (IC_5_)	⇔	⇔
Neurite outgrowth	⇓ n. branch points (IC_5_)	⇓ ⇔	⇔	⇓ n. branch points (IC_20_)	⇔	⇔
BDNF levels	⇔ ⇓ ratio (IC_5_)	⇑ BDNFt (IC_20_) ⇓ ratio (IC_20_)	⇔	⇓ ratio (IC_20_)	⇑ (IC_5_)	⇑ BDNFn and ratio (IC_20_)

Tested concentrations: BPA (0.29 μM, IC_20_/100; 12.74 μM, IC_5_; 28.96 μM, IC_20_); CPF (0.37 μM, IC_20_/100; 21.01 μM, IC_5_; 37.10 μM, IC_20_); Lead (0.0073 μM, IC_20_/100; 0.17 μM, IC_5_; 0.73 μM, IC_20_); Methyl-Hg (0.0013 μM, IC_20_/100; 0.05 μM, IC_5_; 0.13 μM, IC_20_); PCB138 (0.0593 μM, IC_20_/100; 3.53 μM, IC_5_; 5.93 μM, IC_20_); VA (2.1 μM, IC_20_/100; 70 μM, IC_5_; 210 μM, IC_20_)

### Bisphenol a (BPA)

A slight decrease of PSD95 total levels and a tendency towards an increase of SYP in the neurites (not significant) could be observed after 3 day treatment with the highest BPA tested concentration (28.96 μM, IC_20_) compared to solvent control at the respective time point, while 14 day exposure caused a slight decrease of PSD95 at neurite levels with IC_5_ (12.74 μM) (Additional file 2: Figure S1A). The number of synapses (i.e., number of overlapping SYP and PSD95 spots in the neurites) did not significantly change (Additional file 2: Figure S1A).

An increase in the number of neurites (not shown) was visible after 3 days with the IC_5_ treatment, whilst the number of branch points started decreasing after 14 days (Additional file 2: Figure S1B).

Levels of BDNF did not change significantly after 3 or 14 days (Additional file 2: Figure S1C), although a decrease in the ratio between the levels of BDNF in the neurites and in the cell body was observed with IC_5_ (Additional file 2: Figure S1C), possibly resulting in lower levels of BDNF in neurites than in the cell body compared to untreated cells. Notably, this may be indicative of inhibited BDNF axonal transport [25].

### Chlorpyrifos (CPF)

An increase of SYP total levels could be observed at 3 day treatment with the highest tested concentration (37.1 μM, IC_20_), while at 14 days a decrease of SYP at the level of neurites, and an increase of total PSD95 were recorded starting with 21 μM (IC_5_) concentration (Additional file 2: Figure S2A). Moreover, a decrease of SYP/PSD95 co-localisation (i.e., synapses) was observed upon 14 day treatment with IC_20_ (Additional file 2: Figure S2A).

A tendency toward a decrease in neurite outgrowth was visible (although not significant) with all tested concentrations after 3 day and 14 day treatment (Additional file 2: Figure S2B).

Total levels of BDNF increased after 3 and 14 days with the highest tested concentration of CPF (IC_20_) (Additional file 2: Figure S2C), and a decrease of neurite to cell body ratio of BDNF levels was observed in all conditions, being statistically significant at IC_20_ concentration after both 3 days and 14 days (Additional file 2: Figure S2C).

### Lead-II-chloride (Lead)

A decrease of PSD95 levels in the neurites upon treatment even with the lowest concentration (IC_20_/100, 0.0073 μM) of Lead was recorded after 3 day treatment, and an increase of SYP levels could be observed with the highest tested concentration (0.73 μM, IC_20_). The decrease of PSD95 levels in the neurites persisted even upon 14 day treatment (starting from the lowest concentration), while the levels of SYP resulted slightly higher when compared to untreated cells (Additional file 2: Figure S3A). Despite a tendency towards an increase, the co-localisation of SYP/PSD95 did not significantly change (Additional file 2: Figure S3A). Both neurite outgrowth and BDNF protein levels parameters did not significantly change upon treatment with the studied concentrations of Lead (Additional file 2: Figure S3B, C).

### Methylmercury (Methyl-Hg)

While a 3-day treatment with Methyl-Hg at the three tested concentrations did not elicit significant modifications of SYP and PSD95 protein levels and their co-localisation, a decrease of SYP levels in the neurites was observed with IC_5_ (0.05 μM) after 14 days (Additional file 2: Figure S4A). No significant differences in neurites parameters were found after 3 days, while a modest but significant decrease of branch points was recorded after a 14 day treatment with the highest concentration (0.13 μM, IC_20_) (Additional file 2: Figure S4B). While overall BDNF levels did not change under all conditions (Additional file 2: Figure S4C), a decrease in the ratio between the levels of BDNF in the neurites and in the cell body was observed after a 14-day treatment with IC_20_ (Additional file 2: Figure S4C).

### PCB138

Three day treatment with PCB138 did not significantly modify the levels of total SYP and PSD95 proteins, although a modest increase of PSD95 was found at the level of neurites upon treatment with 5.93 μM, IC_20_ concentration (Additional file 2: Figure S5A). After prolonged treatment (14 days), no significant differences in SYP and PSD95 levels and in the number of synapses were recorded (Additional file 2: Figure S5A).

After 3 days, PCB138 induced an increase of neurite outgrowth (i.e., both neurite length and number of branch points) at the lowest concentration (0.0593 μM, IC_20_/100), but these parameters did not significantly change compared to control at the respective time point after a 14-day treatment (Additional file 2: Figure S5B).

An increase in the ratio between the levels of BDNF in the neurites and in the cell body was observed with IC_20_/100 concentration, and after 14 days a modest increase of total BDNF levels was found upon treatment with IC_5_ concentration (Additional file 2: Figure S5C).

### Valproic acid (VA)

VA did not modify the levels of synapse proteins (SYP, PSD95) after 3 or 14 day exposure (Additional file 2: Figure S6A).

An increase in the number of neurites per neuron was found after a 3-day treatment with IC_5_ (0.07 mM, not shown), and the highest concentration (0.21 mM, IC_20_) promoted an increase of both neurite length and the number of branch points. These differences were not recorded after prolonged treatment (14 days) (Additional file 2: Figure S6B).

Levels and distribution of BDNF did not significantly change after 3 days of exposure (Additional file 2: Figure S6C), whilst after 14 days, both BDNF levels in the neurites and BDNF neurite-to-cell body ratio were increased upon treatment with IC_20_ concentration (Additional file 2: Figure S6C).

Based on analysis of statistical significance, we calculated, for each individual chemical, the lowest concentration eliciting a statistically significant modification of at least one of the measured DNT features (Table 2). Such concentrations were specific for each DNT endpoint and for each time point (i.e., 3 days and 14 days) (defined as LOAEC-syn, LOAEC-neu and LOAEC-bdnf) and were used to prepare chemical mixtures (Table 3).

**Table 3:** LOAECs specific for each time interval and DNT endpoint and their serial dilutions tested in Phase 3

**3 days**	**BPA**	**CPF**	**Lead**	**Methyl-Hg**	**PCB138**	**VA**	**(μM)**
**Synaptogenesis**	28.96	37.1	0.007	0.26	5.93	420	**LOAEC-syn**
	14.48	18.55	0.004	0.13	2.97	210	**LOAEC/2-syn**
	7.24	9.28	0.002	0.07	1.48	105	**LOAEC/4-syn**
**Neurite outgrowth**	12.74	74.2	1.46	0.26	0.06	70	**LOAEC-neu**
	6.37	37.1	0.73	0.13	0.03	35	**LOAEC/2-neu**
	3.19	18.55	0.37	0.07	0.015	17.5	**LOAEC/4-neu**
**BDNF levels**	57.92	37.1	1.46	0.26	0.06	420	**LOAEC-bdnf**
	28.96	18.55	0.73	0.13	0.03	210	**LOAEC/2-bdnf**
	14.48	9.28	0.37	0.07	0.015	105	**LOAEC/4-bdnf**
**14 days**	**BPA**	**CPF**	**Lead**	**Methyl-Hg**	**PCB138**	**VA**	(μM)
**Synaptogenesis**	12.74	21.01	0.007	0.05	0.06	2.1	**LOAEC-syn**
	6.37	10.51	0.004	0.025	0.03	1.05	**LOAEC/2-syn**
**Neurite outgrowth**	12.74	74.2	1.46	0.13	11.86	420	**LOAEC-neu**
	6.37	37.1	0.73	0.07	5.93	210	**LOAEC/2-neu**
	3.19	18.55	0.37	0.03	2.97	105	**LOAEC/4-neu**
	1.59	9.28	0.18	0.016	1.48	52.5	**LOAEC/8-neu**
**BDNF levels**	12.74	37.1	1.46	0.13	3.53	210	**LOAEC-bdnf**
	6.37	18.55	0.73	0.07	1.77	105	**LOAEC/2-bdnf**
	3.19	9.28	0.37	0.03	0.88	52.5	**LOAEC/4-bdnf**

### Effects of mixtures on cell viability (phase 3)

We sought to investigate whether single chemicals tested in Phases 1 and 2 elicited DNT effects on hiPSC-derived NSCs undergoing differentiation when added in mixtures. Therefore, we mixed together chemicals at concentrations starting from the LOAEC-syn, LOAEC-neu and LOAEC-bdnf calculated at the end of Phase 2, and further diluted the obtained mixtures if necessary, as described below.

In detail, cells were again treated for either 3 or 14 days with individual chemicals (internal control for mixtures) and the following three different types of mixtures: (i) a mixture containing 3 chemicals with similar MoA (‘3-Sim’); (ii) a mixture containing 3 chemicals with dissimilar MoAs (‘3-Diss’), and (iii) a mixture containing all 6 chemicals together (‘All’). When a LOAEC for a certain chemical and DNT endpoint could not be immediately identified (due to lack of statistical significance in Phase 2), the highest concentrations tested in Phase 3 (IC_20_) were multiplied by a factor of two. For instance, this applied to CPF in relation to the analysis of neurite outgrowth: as no significant differences of neurite-related parameters were recorded after any studied concentration, therefore the IC_20_ of CPF was multiplied by two (i.e., 37.1 (× 2) = 74.2 μM) and was retained as the highest concentration (putative LOAEC-neu) to be tested in Phase 3 (see Table 3). In contrast, if the mixtures were highly cytotoxic, LOAEC concentrations were diluted by a factor of 2, 4, or even 8, if necessary.

**Fig. 5:**
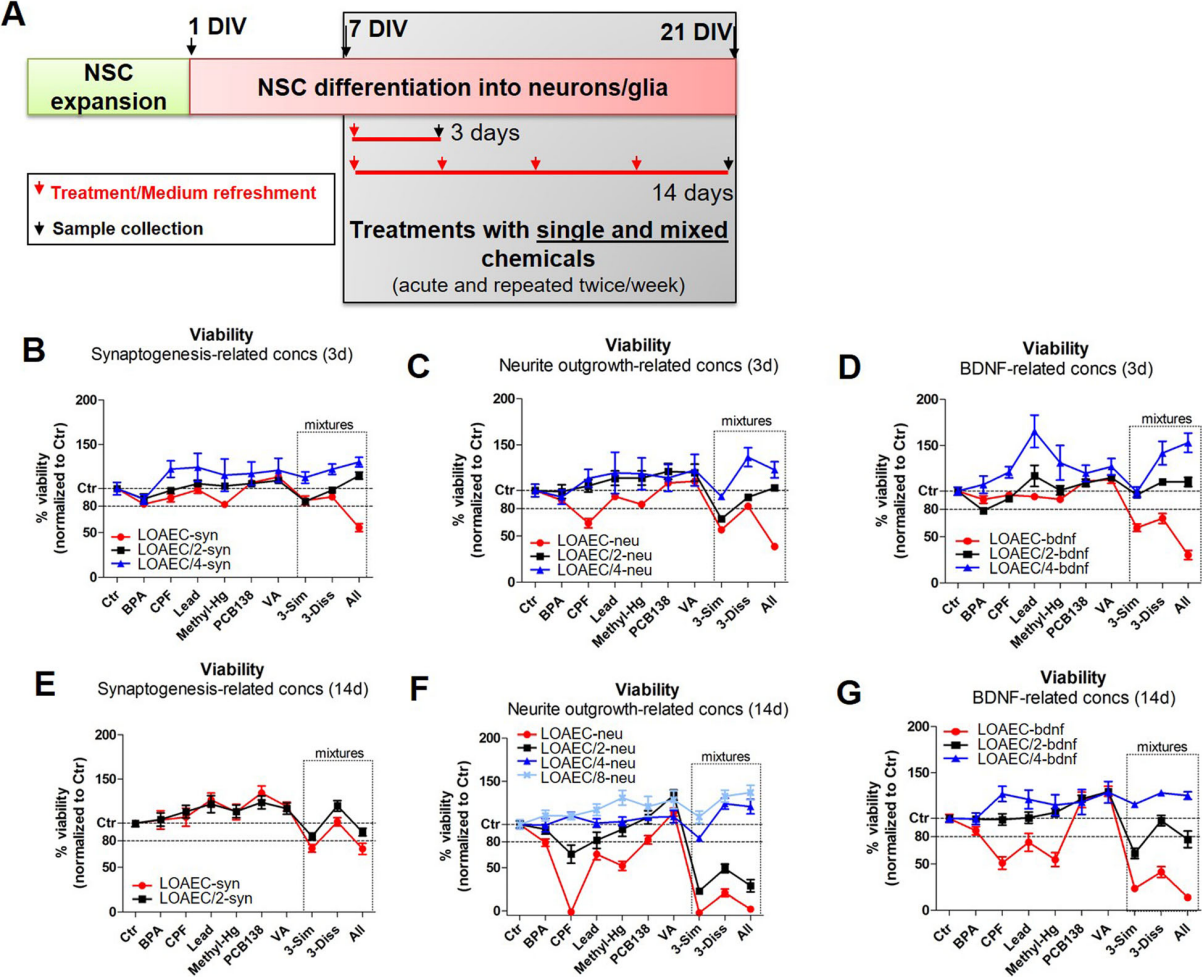
Analysis of cell viability upon treatments with mixtures using CellTiter-Blue assay. (**a**) hiPSC-derived NSCs were differentiated for 7 DIV and treated with individual chemicals or three different types of mixtures for either 3 days (**b**-**d**) or 14 days (**e**-**g**) with different LOAECs specific for each DNT endpoint (i.e., synaptogenesis (**b** and **e**), neurite outgrowth (**c** and **f**), and BDNF levels (**d** and **g**)). After 3 days (**b**-**d**) or 14 days (**e**-**g**), resazurin test (with CellTiter Blue) was performed. All samples were normalised to control medium with solvent (0.1% DMSO, Ctr) at the respective time point. LOAECs (red curves) and their serial dilutions (respectively black (LOAEC/2), blue (LOAEC/4) and light blue (LOAEC/8) curves) were tested to assess whether mixed chemicals elicited cytotoxic effects. Mixture labelled as ‘3-Sim’ contained similar MoA chemicals (affecting BDNF levels i.e., BPA, CPF and Lead), whilst mixture with dissimilar MoA chemicals (i.e., Methyl-Hg, PCB138 and VA) is labelled as ‘3-Diss’. The ‘All’ mixture comprised all 6 chemicals together. Data are represented as mean ± S.E.M. of 3–4 biological replicates

Cytotoxicity after 3 day (acute) and 14 day (repeated dose) of treatment was determined in order to evaluate the possible cytotoxic effects elicited by mixtures compared to individual chemicals (Fig. 5). In general, the mixture comprising the three chemicals with similar MoA (i.e., BPA, CPF and Lead) reduced cell viability more potently than the mixture with dissimilar MoA chemicals (i.e., Methyl-Hg, PCB138 and VA). Additionally, some of the tested mixtures (e.g., neurite-related and BDNF-related LOAECs mixtures, red curves in Fig. 5f, g) were extremely cytotoxic (with > 80% reduction of cell viability) and therefore were not further considered for the assessment of the effects of mixtures on the selected DNT endpoints.

### Effects of mixtures on DNT specific endpoints (phase 3)

#### Synaptogenesis (SYP and PSD95)

**Fig. 6:**
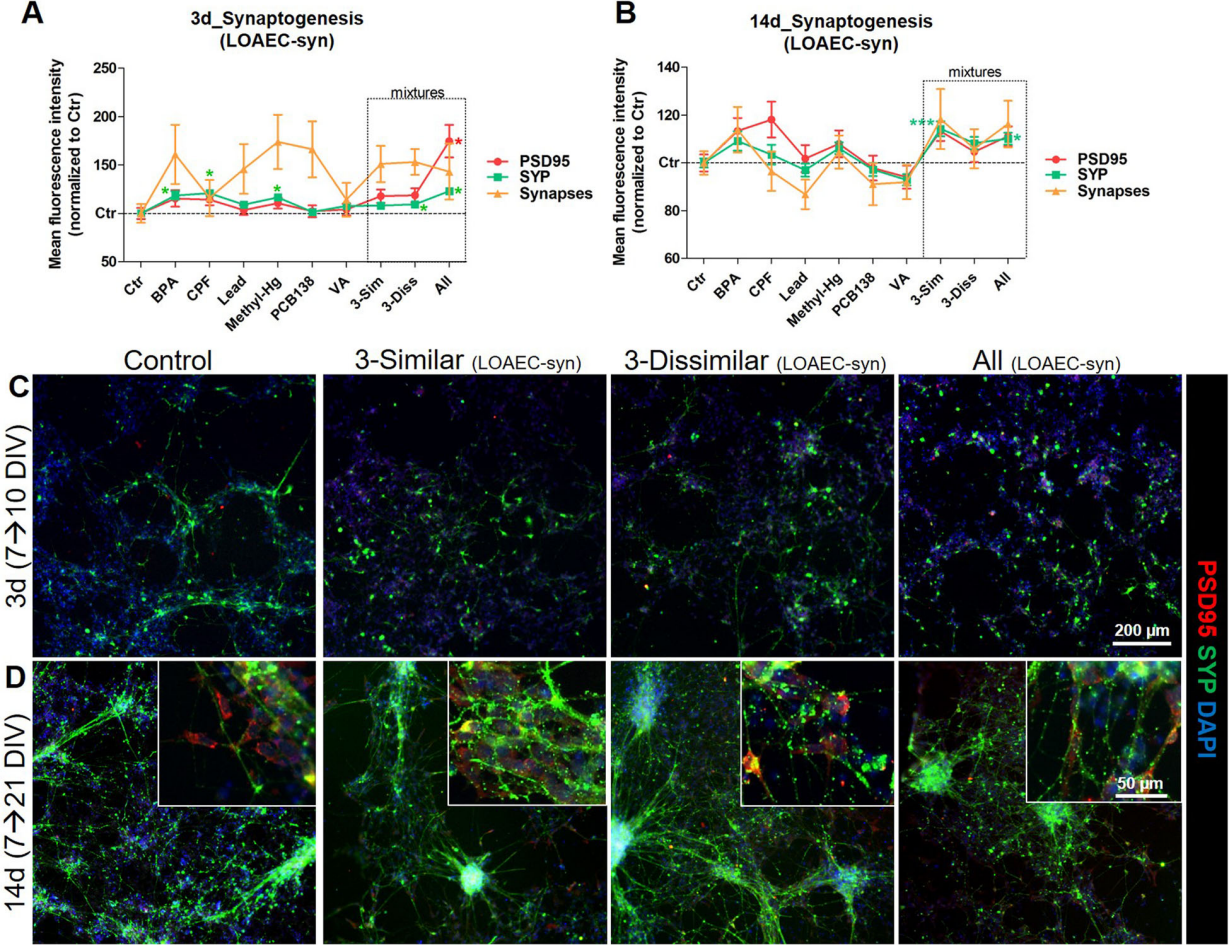
Mixture effects on synaptogenesis. hiPSC-derived NSCs were differentiated for 7 DIV, and then treated for either 3 days (**a** and **c**) or 14 days (**b** and **d**) with single chemicals (BPA, CPF, Lead, Methyl-Hg, PCB138 and VA) and three types of mixtures: (i) a mixture with the 3 similar MoA chemicals (labelled ‘3-Sim’), (ii) a mixture with the 3 dissimilar MoA chemicals (labelled ‘3-Diss’), and (iii) a mixture with all 6 chemicals (labelled ‘All’). (**a**, **b**) Graphs reporting total levels of PSD95 (red), total levels of SYP (green) and number of overlapping SYP/PSD95 spots (synapses, yellow) analyzed upon treatment with LOAEC-syn concentrations. (**c**, **d**) Representative immunocytochemical image (at 10x magnification, with 40x magnifications insets) of cells treated with mixtures at different LOAEC-syn concentrations (see Table 3) for either 3 days (**c**) or 14 days (**d**) and stained for PSD95 (red) and SYP (green). All samples were normalised to solvent control (0.1% DMSO, Ctr) at the respective time point. Data are represented as mean ± S.E.M. of 3–4 biological replicates

After 3 day treatment (Fig. 6a, c), individual chemicals, BPA, CPF, Methyl-Hg or PCB138 already at LOAEC/2-syn concentrations induced an increase of total SYP at levels comparable to the increase of SYP elicited by the mixture containing the 3 similar (‘3-Sim’) or the one with the 3 dissimilar (‘3-Diss’) MoA chemicals (Additional file 3: Figure S7B, black curve). This suggests that those chemicals may be the main drivers of observed increased SYP expression in the neurons exposed to these mixtures. Moreover, individual chemicals at LOAEC/4-syn (i.e., LOAEC-syn concentration diluted 4 times; blue curves, Additional file 3: Figure S7A-C) did not elicit significant changes of synapogenesis, which, on the contrary, was impacted by exposing the cells to the three types of mixtures. In particular, the ‘3-Sim’ mixture at low concentrations (LOAEC/4-syn) caused a significant increase of both SYP levels and the number of SYP/PSD95 overlapping spots in the neurites (i.e., synapses), while these effects were less prominent upon treatment with ‘3-Diss’ mixture and the mixture containing all chemicals together (‘All’) (blue curves, Additional file 3: Figure S7B, C). The levels of PSD95 were upregulated upon treatment with the ‘All’ mixture already at LOAEC/2-syn (black curve, Additional file 3: Figure S7A), but did not change under the other conditions.

After a prolonged treatment (14 days), while individual chemicals did not elicit significant effects, mixtures at LOAEC-syn concentrations with similar MoA chemicals (‘3-Sim’) and ‘All’ promoted an increase of SYP levels, a tendency towards increase of PSD95 and their overlapping (marker of synapses) although not significant (Fig. 6b, d and Additional file 3: Figure S7D-F). Notably, at LOAEC/2-syn, while PSD95 levels did not significantly change, an increase of SYP levels was observed upon single treatment with BPA and in ‘3-Sim’ mixtures (Additional file 3: Figure S7E, black curve), suggesting that BPA may be, under these conditions, the main driver of increased synaptogenesis.

#### Neurite outgrowth

**Fig. 7:**
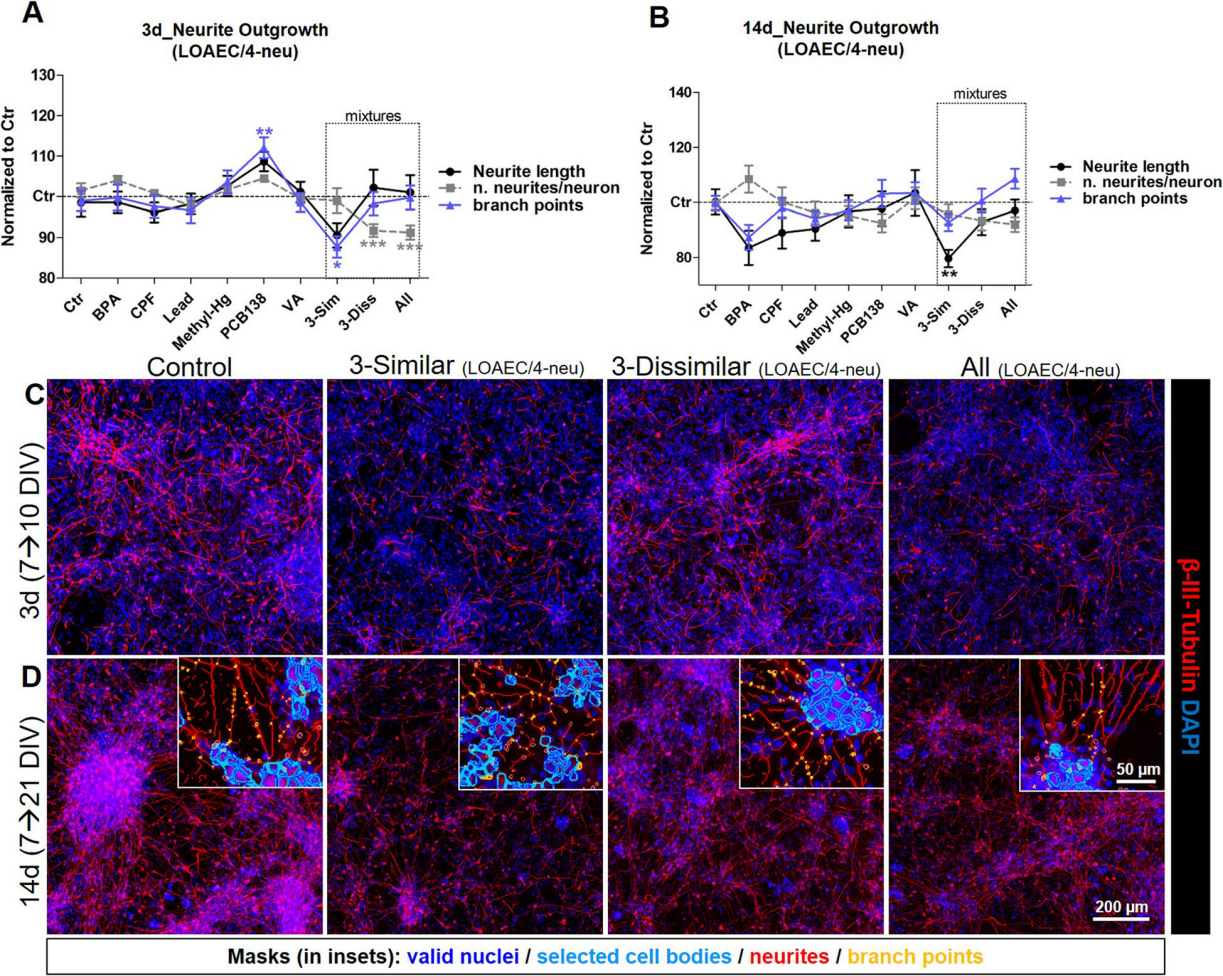
Mixture effects on neurite outgrowth. hiPSC-derived NSCs were differentiated for 7 DIV, and then treated for either 3 days (**a**, **c**) or 14 days (**b**, **d**) with single chemicals (BPA, CPF, Lead, Methyl-Hg, PCB138 or VA) and three types of mixtures: (i) a mixture with the 3 similar MoA chemicals (labelled ‘3-Sim’), (ii) a mixture with the 3 dissimilar MoA chemicals (labelled ‘3-Diss’), and (iii) a mixture with all 6 chemicals (labelled ‘All’). (**a**, **b**) Graphs reporting neurite length (black), the number of branch points/neurite (grey), and the number of neurites/neuron (violet curves) analysed upon treatments with LOAEC/4-neu concentrations (see Table 3). (**c**, **d**) Representative immunocytochemical image (at 10x magnification, with 40x magnifications insets showing applied masks for detection of neurites) of cells treated with neurite outgrowth-related mixtures (at LOAEC/4-neu concentrations) for either 3 days (**c**) or 14 days (**d**) and stained for β-III-Tubulin (red). All samples were normalised to medium containing solvent only (0.1% DMSO, Ctr). Data are represented as mean ± S.E.M. of 3–4 biological replicates

After 3 day treatment, the ‘3-Sim’ mixture was found to downregulate both neurite length and the number of branch points per neurite more than the ‘3-Diss’ mixture (already at LOAEC/4-neu, Fig. 7a and c, and Additional file 3: Figure S8A and B, blue curves), while the the number of neurites/neuron decreased upon treatment with the ‘3-Diss’ and the ‘All’ mixtures (already at LOAEC/4-neu, Fig. 7a and Additional file 3: Figure S8C). Notably, CPF alone at LOAEC/2-neu elicited effects similar to those observed upon ‘3-Sim’ mixture treatment, indicating that among the other chemicals present in the ‘3-Sim’ and ‘All’ mixtures, CPF could be the one driving the most neurotoxic effects (black curves in Additional file 3: Figure S8A-C). Conversely, PCB138, individually tested, already at LOAEC/4-neu was found to elicit a slight increase of both neurite length and the number of branch points (blue curves, Additional file 3: Figure S8A, B). This suggests that after 3 day treatment, PCB138 may trigger opposite effects in both mixtures ‘3-Diss’ and ‘All’. Despite the observed downregulation of neurite features, interestingly the percentage of β-III-tubulin^+^ neurons was found upregulated mainly upon exposure to ‘3-Sim’ mixture (at LOAEC/2-neu concentrations, by 26 ± 4%) at levels comparable to the upregulation induced by individually tested CPF (Additional file 3: Figure S8D). The mixture containing all 6 chemicals together induced a statistically significant upregulation of β-III-tubulin^+^ neurons only at LOAEC-neu concentrations (by 24 ± 4%, Additional file 3: Figure S8D, red curve). Conversely, exposure to ‘3-Diss’ mixture, at all tested concentrations, did not cause significant variations of neuronal cell numbers (Additional file 3: Figure S8D) after a 3-day exposure.

The decrease of neurite outgrowth parameters was even more prominent after a prolonged treatment (14 days) (Fig. 7b, d), with the ‘3-Sim’ mixture causing the highest downregulation of neurite length already at LOAEC/4-neu, (Additional file 3: Figure S8E, dark blue curve). Notably, the downregulating effects of ‘3-Sim’ mixture on neurite length and on the number of branch points/neurite were even more prominent than those induced by the ‘All’ mixture (see black and dark blue curves in Additional file 3: Figure S8E, F), which could be due to opposite effects induced by VA (present in the ‘All’ mixture, but not in the ‘3-Sim’ mixture), which, when individually tested, was found to increase neurite outgrowth (at LOAEC/2-neu, black curves in Additional file 3: Figure S8E, F). Analysis of the proportion of β-III-tubulin^+^ neuronal cells revealed an increase upon treatment with the three mixtures at LOAEC/2-neu (respectively, by 55 ± 7% with ‘3-Sim’; by 44 ± 2% with ‘3-Diss’; by 49 ± 3% with ‘All’) (Additional file 3: Figure S8H), when compared with cells exposed to individual chemicals or to solvent control at the respective time point. On the other hand, the proportion of GFAP^+^ cells (i.e., astrocytes) did not significantly change upon any mixture exposure (not shown).

Taking all results into account, it is conceivable to hypothesise that, after a 14-day treatment at the selected concentrations, CPF (followed by Lead) seems to be the main driver of decreased neurite outgrowth upon exposure to the mixtures of ‘3-Sim’ and ‘All’, possibly partly counteracted by VA present in both mixtures ‘3-Diss’ and ‘All’.

To confirm whether CPF and VA could be considered as respectively the main driver of neurotoxicity and the chemical with the major counteracting effects in the mixtures, cells were treated for 14 days with: (i) the ‘3-Sim’ mixture and a mixture of only BPA and Lead (without CPF, similar MoA chemicals), (ii) the ‘3-Diss’ mixture and a mixture of only Methyl-Hg and PCB138 (without VA, dissimilar MoA chemicals), and (iii) the ‘All’ mixture and a mixture of BPA, Lead, Methyl-Hg and PCB138 (excluding both CPF and VA). The LOAEC/2-neu and LOAEC/4-neu concentrations relevant to measure neurite outgrowth after 14 day treatment were considered for this experiment (see Table 3). The obtained data indicated that, compared to the original ‘3-Sim’ mixture, CPF withdrawal increased the number of live cells by 23 ± 7% (DAPI+ non-pyknotic nuclei) at LOAEC/2-neu concentrations (Additional file 3: Figure S9A, B, black bars). Live cell number also increased (by 31 ± 5%, vs ‘3-Diss’ mixture) in the absence of VA at LOAEC/2-neu concentrations, (Additional file 3: Figure S9A, B), and by 2.5 fold in the absence of both chemicals, compared to the ‘All’ mixture (at LOAEC/2-neu concentrations, Additional file 3: Figure S9A, B).

By withdrawing CPF, both neurite length (Additional file 3: Figure S9C, black bars) and the number of branch points/neurite (Additional file 3: Figure S9D, black bars) only modestly (not significantly) increased compared to the ‘3-Sim’ mixture. Moreover, the number of neurites/neuron, which was found to increase in the ‘3-Sim’ mixture (at LOAEC/2-neu, see Additional file 3: Figure S8G), resulted only slightly lower in the absence of CPF (Additional file 3: Figure S9E, black bars).

On the contrary, in the absence of VA, both neurite length and the number of branch points/neurite decreased in comparison with the original ‘3-Diss’ mixture effects (respectively by 14 ± 3% and 7 ± 4%) (Additional file 3: Figure S9C, D, black bars). Similar effects were observed by withdrawing both VA and CPF from the ‘All’ mixture, with 15 ± 4% decrease of neurite length, 17 ± 5% decrease of branch points, and also 23 ± 3% decrease of neurites/neuron in comparison with the ‘All’ mixture (Additional file 3: Figure S9C-E, black bars). These differences were very modest (generally not significant) upon treatments with LOAEC/4 concentrations (Additional file 3: Figure S9C-E, blue bars).

Altogether, these data suggest that VA may truly counteract the decrease of neurite features (i.e., neurite length and branch points/neurite) observed in mixtures, while withdrawal of CPF from the mixtures seems to modestly reverse the decrease of neurite outgrowth.

#### BDNF levels

**Fig. 8:**
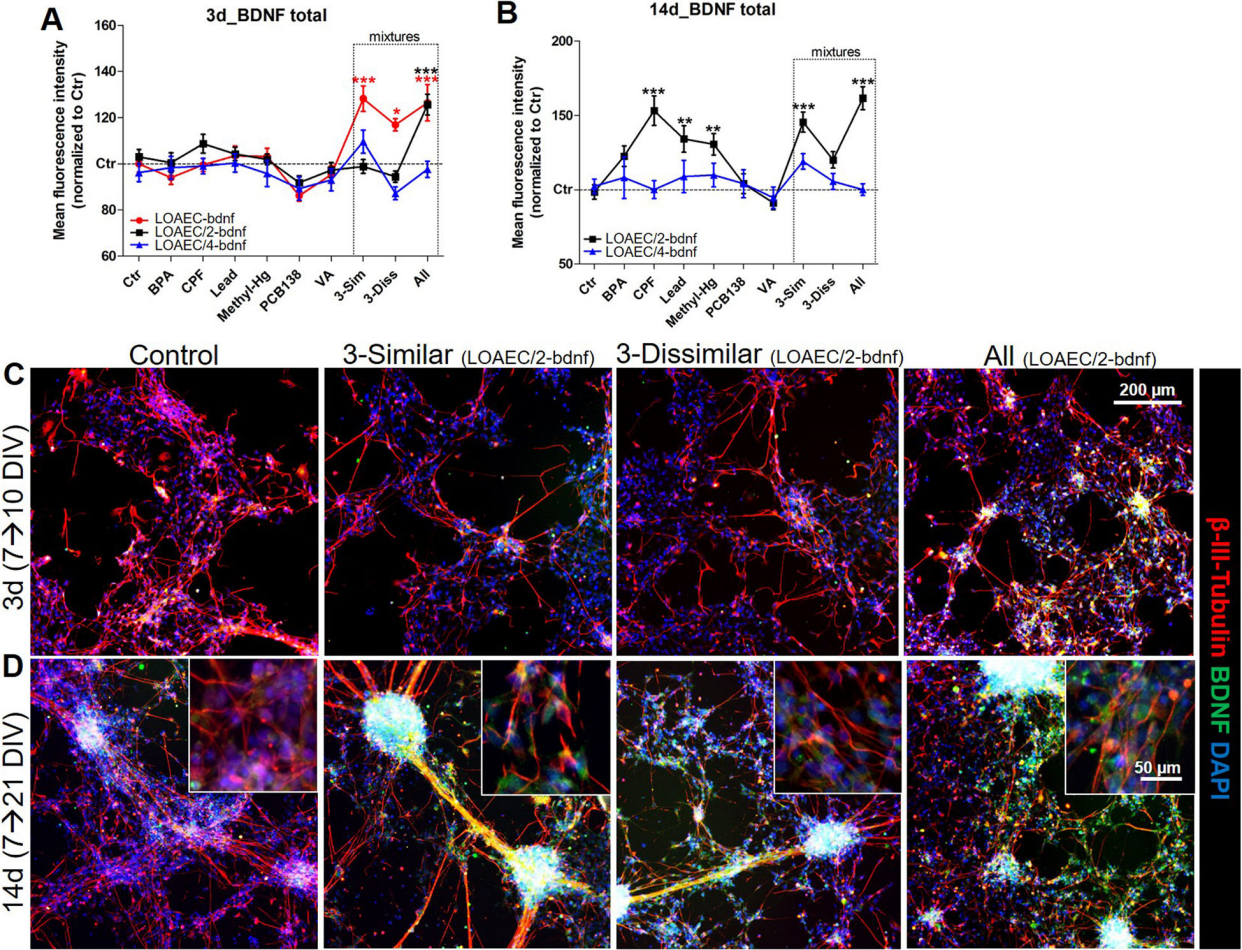
Mixture effects on BDNF levels. hiPSC-derived NSCs were differentiated for 7 DIV, and then treated for either 3 days (A, C) or 14 days (**b**, **d**) with single chemicals (BPA, CPF, Lead, Methyl-Hg, PCB138 and VA) or three types of mixtures: (i) a mixture with the 3 similar MoA chemicals (labelled ‘3-Sim’), (ii) a mixture with the 3 dissimilar MoA chemicals (labelled ‘3-Diss’), and (iii) a mixture with all 6 chemicals (labelled ‘All’). (A, B) Graphs show BDNF total levels measured upon treatment with single chemicals or mixtures at LOAEC-bdnf (red curve, only after 3 day treatment) and their serial dilutions (respectively black (LOAEC/2-bdnf) and blue (LOAEC/4-bdnf) curves). (**c**, **d**) Representative immunocytochemical image (at 10x magnification, with 40x magnifications insets) of cells treated with BDNF-related mixtures (at LOAEC/2-bdnf concentrations) for either 3 days (**c**) or 14 days (**d**) and stained for β-III-Tubulin (red) and BDFN (green). For the analyses, all samples were normalised to solvent control (0.1% DMSO, Ctr) at the respective time point. Data are represented as mean ± S.E.M. of 3–4 biological replicates

Total BDNF levels were found upregulated after 3 day-exposure to both the ‘3-Sim’ (by 28 ± 5% at LOAEC-bdnf) and the ‘All’ chemical (by 26 ± 8% at LOAEC-bdnf) mixtures when compared to ‘3-Diss’ mixture (17 ± 3% increase at LOAEC-bdnf) (red curves, Fig. 8a). At LOAEC/2-bdnf, upregulation of BDNF was osberved only upon exposure to the ‘All’ mixture (25 ± 4% increase) (black curves, Fig. 8a, c).

After 14-day exposure, at concentrations below LOAEC-bdnf (i.e., LOAEC/2-bdnf), CPF alone resulted to be the strongest inducer of BDNF levels (53 ± 9% increase) followed by Lead and Methyl-Hg (Fig. 8b), and this increase of BDNF levels was comparable to the one observed upon treatment with the ‘3-Sim’ (45 ± 6% increase) and ‘All’ mixtures (61 ± 7% increase) (black curves, Fig. 8b) (representative images shown in Fig. 8d). On the other hand, treatment with ‘3-Diss’ mixture only modestly increased BDNF levels (by 20 ± 5%) (not significant) (Fig. 8b). These results suggest that CPF may be the main driver of increased BDNF levels induced by the ‘3-Sim’ and ‘All’ mixtures.

Table 4 summarises the most significant effects along with the LOAEC calculated for each mixture and for each measured endpoint.

**Table 4:** Main effects and mixture-related LOAECs specific for each time interval and DNT endpoint

**3 days**	**‘3-Sim’** **Similar MoA**	**‘3-Diss’** **Dissimilar MoA**	**‘All’**
**Synaptogenesis** **(SYP, PSD95)**	⇑ SYPt ⇑ synapses (LOAEC/4-syn) BPA, CPF	⇑ SYPt ⇑ synapses (LOAEC/4-syn) Methyl-Hg, PCB138	⇑ SYPt (LOAEC/4-syn) BPA, CPF, Methyl-Hg, PCB138
**Neurite outgrowth and % of neurons**	⇓ n. branch points ⇓ neurite length (LOAEC/4-neu) ⇑ β-III-tubulin+ neurons (LOAEC/2-neu) CPF	⇓ n. neurites/neuron (LOAEC/4-neu) PCB138 (counteract)	⇓ n. neurites/neuron (LOAEC/4-neu) ⇑ β-III-tubulin^+^ neurons (LOAEC-neu) CPF PCB138 (counteract)
**BDNF levels**	⇑ total BDNF (LOAEC-bdnf)	⇑ total BDNF (LOAEC-bdnf)	⇑ total BDNF (LOAEC/2-bdnf)
**14 days**	‘3-Sim’ Similar MoA	‘3-Diss’ Dissimilar MoA	‘All’
**Synaptogenesis** **(SYP, PSD95)**	⇑ SYPt (LOAEC/2-syn) **BPA**	⇔	⇑ SYPt (LOAEC-syn) **BPA**
**Neurite outgrowth and % of neurons**	⇓ neurite length (LOAEC/4-neu) ⇑ β-III-tubulin+ neurons (LOAEC/2-neu) **CPF**, Lead	⇓ n. branch points ⇓ neurite length ⇑ β-III-tubulin+ neurons (LOAEC/2-neu) Methyl-Hg, PCB138, VA (counteract)	⇓ n. branch points ⇓ neurite length ⇑ β-III-tubulin^+^ neurons (LOAEC/2-neu) **CPF**, Lead, Methyl-Hg, PCB138 VA (counteract)
**BDNF levels**	⇑ total BDNF (LOAEC/2-bdnf) **CPF**, Lead	⇔ ⇑ total BDNF (LOAEC/2-bdnf) Methyl-Hg	⇑ total BDNF (LOAEC/2-bdnf) **CPF**, Lead, Methyl-Hg

The chemicals hypothesised to be the main drivers of toxicity in the mixture are indicated in Table 4. Where indicated, VA and PCB138 may induce counteracting effects.

#### Assessment of mixture effects via mathematical modelling

**Fig. 9:**
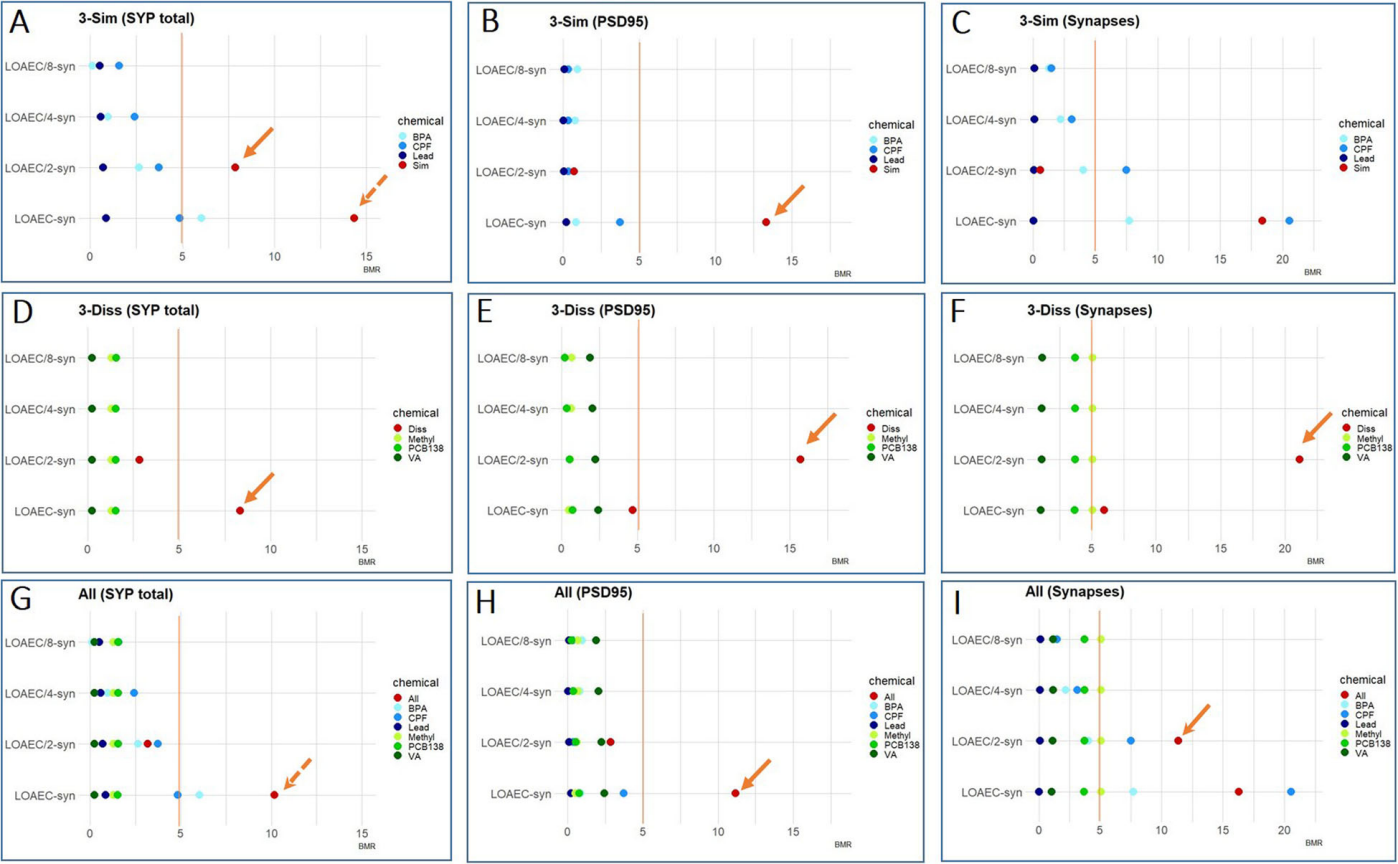
Bench mark responses (BMR) for synaptogenesis. The absolute BMR of single chemicals, calculated considering the concentrations used in the mixtures ('3-Sim' in **a**-**c**, '3-Diss' in **d**-**f**, and 'All' in **g**-**i**), are plotted and compared with the absolute percentage of response experimentally observed in the mixtures for each synaptogenesis feature (normalised to control), respectively: total SYP levels (**a**, **d**, **g**), total PSD95 levels (**b**, **e**, **h**), and number of synapses (**c**, **f**, **i**). The orange line represents the threshold of 5%, corresponding to a Toxic Unit (TU) = 1. According to this approach, when the TU calculated on the basis of single chemical contribution is ≤1, and the percentage of response experimentally observed in the mixtures is > 1, a synergistic effect can be predicted (highlighted by solid arrows). This was observed for the following conditions: total SYP, upon treatment with 3-Sim mixture at LOAEC/2-syn (**a**) and 3-Diss mixture at LOAEC-syn (**d**); total PSD95, upon treatment with 3-Sim mixture at LOAEC-syn (**b**), 3-Diss mixture at LOAEC/2-syn (**e**), and All mixture at LOAEC-syn (**h**); synapses, upon treatment with 3-Diss mixture at LOAEC/2-syn (**f**) and with All mixture at LOAEC/2-syn (**i**)

To evaluate the potency of the individual chemicals in the three tested mixtures, we calculated, for each DNT endpoint, the Bench Mark Response (BMR) of single chemicals considering the concentrations used in the mixtures, as described in materials and methods. This approach enabled the evaluation of single chemical potency, expressed as BMR, and their individual contribution to mixture effects (normalized to untreated control). If tested mixture effects were below the threshold (TU ≤ 1) and the observed mixture response was higher than 5%, this suggested synergistic interactions. Notably, by applying this approach, data showed that only for synaptogenesis a synergistic effect could be identified. In particular for the following features (see solid arrows): total SYP, upon treatment with 3-Sim mixture at LOAEC/2-syn (Fig. 9a) and 3-Diss mixture at LOAEC-syn (Fig. 9d); total PSD95, upon treatment with 3-Sim mixture at LOAEC-syn (Fig. 9b), 3-Diss mixture at LOAEC/2-syn (Fig. 9e), and All mixture at LOAEC-syn (Fig. 9h); synapses, upon treatment with 3-Diss mixture at LOAEC/2-syn (Fig. 9f) and with All mixture at LOAEC/2-syn (Fig. 9i).

**Fig. 10:**
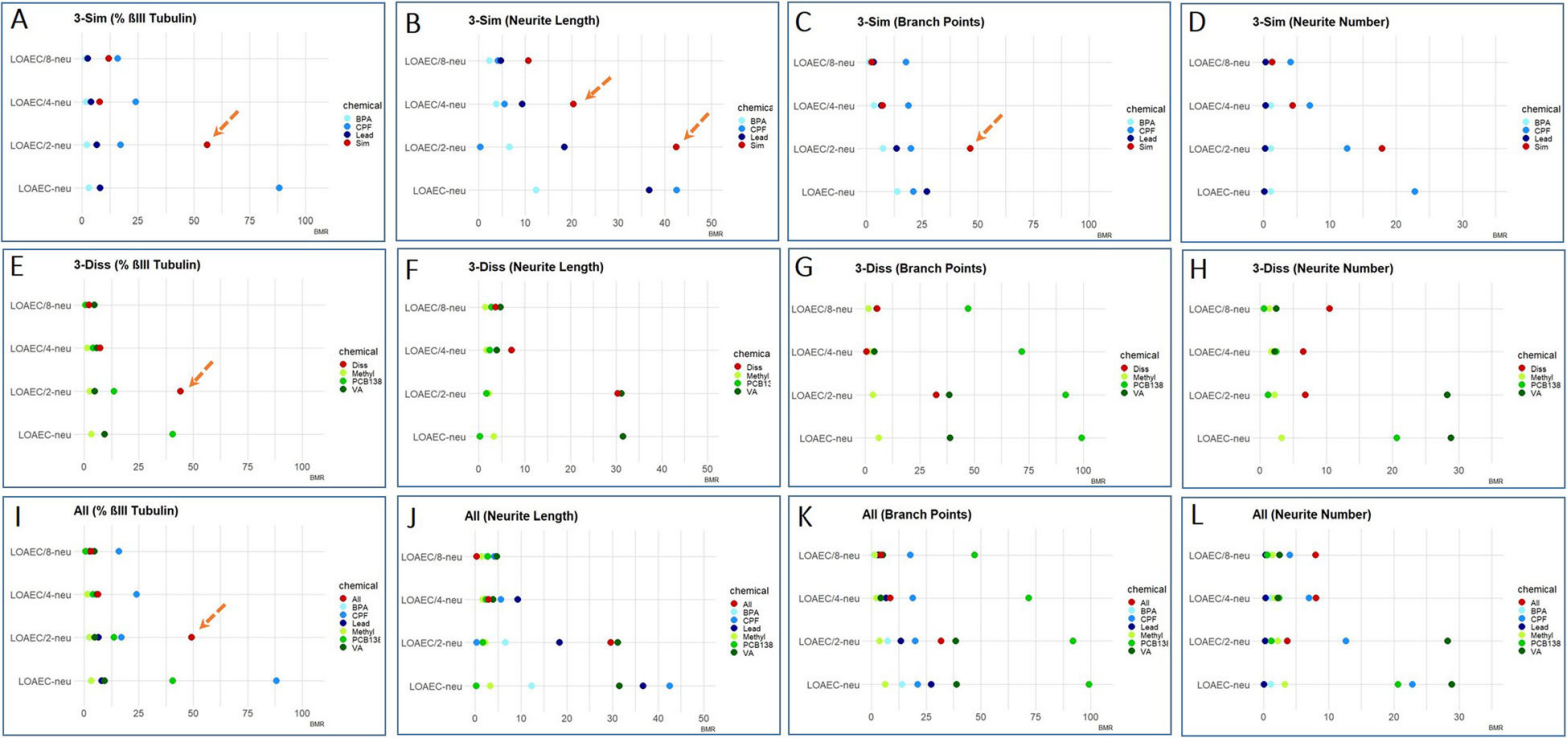
Bench mark responses (BMR) for % of neurons and neurite outgrowth. The absolute BMR value of single chemicals, calculated considering their concentrations used in the mixtures ('3-Sim' in **a**-**d**, '3-Diss' in **e**-**h**, and 'All' in **i**-**l**), are plotted and compared with the absolute percentage of response observed experimentally in the mixtures for each neuronal endpoint (normalised to control), respectively: % of β-III-tubulin+ (**a**, **e**, **i**), neurite length (**b**, **f**, **j**), number of branch points/neurite (**c**,  **g**, **k**), and number of neurites/neuron (**d**, **h**, **l**). For these endpoints the evaluation of the contribution of single chemicals revealed a response above the 5% threshold (TU > 1), and therefore mixture effects cannot be predicted by this model. However, a combined/potentiated mixture effect can be hypothesized, in the case when the response elicited by the mixture was at least two-folds of magnitude higher than individual chemical contributions, indicated by the orange dashed arrows

**Fig. 11:**
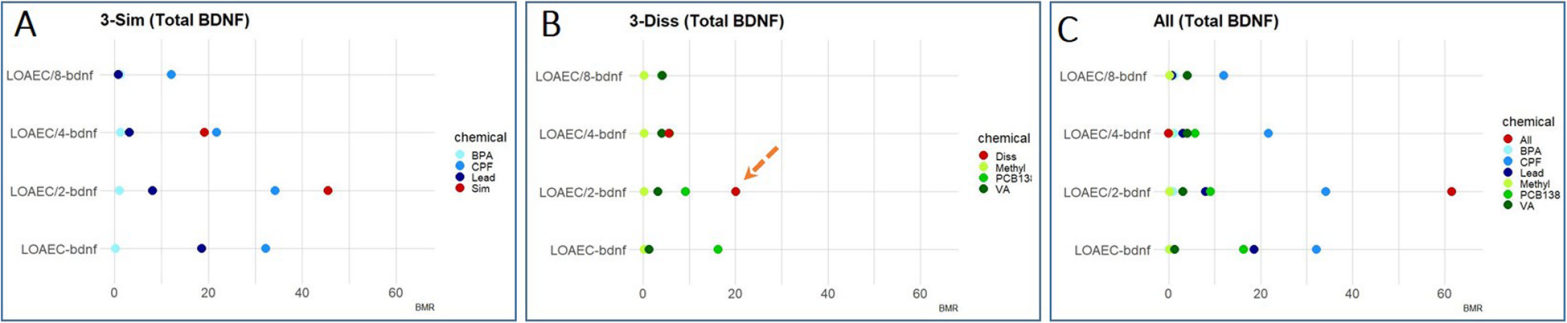
Bench mark response (BMR) for BDNF protein levels. The absolute BMR of single chemicals, based on the concentrations used in the mixtures ('3-Sim' in **a**, '3-Diss' in **b**, and 'All' in **c**), are plotted and compared with the absolute percentage of response experimentally observed in the mixtures for BDNF (normalised to control). Although the calculated TU was > 1, a combined/potentiated mixture effect can be hypothesized, in the case when the response elicited by the mixture was at least two-folds of magnitude higher than individual chemical contributions, as indicated by the orange dashed arrow

On the other hand, for all the other DNT endpoints, in particular the % of neuronal β-III-tubulin+ cells (Fig. 10a, e, i), neurite outgrowth-related features (Fig. 10b, c, d, f, g, h, j, k, l), and total BDNF levels (Fig. 11a-c), the calculated TU resulted > 1, preventing the possibility to model synergism.

Although the additive concentration approach and the TU model were not applicable for these other endpoints, we hypothesized a combined/potentiated mixture effect whenever the response elicited by the mixture was at least two-folds of magnitude higher than individual chemical contributions. This was considered in particular for the following DNT endpoints (indicated with a dashed arrow): SYP levels upon treatment with 3-Sim and All mixtures at LOAEC-syn (Fig. 9a and g); % of β-III-tubulin+ cells upon treatment with 3-Sim mixture at LOAEC/2-neu (Fig. 10a), 3-Diss mixture at LOAEC/2-neu (Fig. 10e), and All mixture at LOAEC/2-neu (Fig. 10i); neurite length upon treatment with 3-Sim mixture at LOAEC/2-neu and LOAEC/4-neu (Fig. 10b); the number of branch points/neurite upon treatment with 3-Sim mixture at LOAEC/2-neu (Fig. 10c); and BDNF levels upon treatment with 3-Diss mixture at LOAEC/2-bdnf (Fig. 11b).

Altogether, these data suggest that the synaptogenesis is the most sensitive DNT endpoint to chemical mixture-induced effects, as confirmed by both experimental data and mathematical modelling. On the other hand, although induction of synergistic effects of mixtures on neuronal cell percentage, neurite outgrowth and BDNF levels could not be confirmed by mathematical modelling, some combined/potentiated mixture effects on these DNT endpoints can be hypothesized based on experimental data, as discussed above.

## Discussion

Data reported in this in vitro study suggest that individual chemicals, representing different classes (industrial chemicals, pesticides, EDs, drugs and POPs) at very low cytotoxic concentrations (relevant to human exposure), may become developmental neurotoxicants in a mixture. The obtained results indicate that testing single chemical-induced toxicity is not sufficient, since the exposure to mixtures produces higher level of toxicity, and the human population, obviously including pregnant woman, infants and children, is exposed to multiple chemicals at the same time. In other words, testing individual chemicals does not reflect real life exposures. As shown in this study, key neurodevelopmental processes, such as neuronal morphological differentiation and formation of synapses (synaptogenesis), are vulnerable stages of brain development that are affected by chemical mixtures, especially when working through similar MoA. Indeed, already after an acute treatment (3 days), increases in pre-synaptic (SYP) and post-synaptic (PSD95) protein levels were observed upon treatment with the similar MoA mixture (‘3-Sim’) as well as the mixture containing all 6 chemicals (‘All’) (Fig. 6 and Additional file 3: Figure S7A, B, D, E). Notably, an increase of SYP or PSD95 above levels found in control cultures at the respective time point could be indicative of altered synaptogenesis, followed up by possible aberrant neuronal network function, as already reported upon exposure to single chemicals, such as BPA [43], VA [44], and perinatal lead exposure [45]. These effects were more prominent in the ‘3-Sim’ and ‘All’ mixtures, after 14 days of exposure, possibly linked to the induction of combined effects elicited by repeated exposure to the chemicals present. Moreover, upon mixture exposure, SYP/PSD95 co-localisation (biomarker of synapses) was mainly present at the perikaryon level (i.e., cell body) rather than at the neurite levels (Additional file 3: Figure S7C, F), suggesting a possible impairment of vesicular transport along axon, resulting in lower number of synapses established at dendrites that could result in alterations of neuronal network formation and function.

Furthermore, alteration of synaptogenesis could also be due to decreased neurite outgrowth found after exposure to the same ‘3-Sim’ chemicals (more potent than dissimilar MoA ones), particularly after the 14-day exposure. Importantly, the effects elicited by CPF alone did not significantly differ from those elicited by the ‘3-Sim’ and the ‘All’ mixtures (see e.g., neurite length and number of branch points, in Additional file 3: Figure S8E, F), suggesting that for these specific DNT features (alteration of synaptogenesis and decrease of neurite outgrowth), CPF (followed by Lead) could be considered as the most plausible driver of toxicity in the 3-Sim mixture under the tested exposure conditions. CPF, like other organophosphates, works mainly through inhibition of acetylcholinesterase (AChE). However, some studies have also shown that, at low concentrations, it works via non-cholinesterase mechanisms. CPF can increase CREB phosphorylation (i.e., affecting BDNF levels) in primary cortical and hippocampal neurons [46], and inhibit neurite outgrowth in PC12 cells [47] and primary cultures of embryonic rat sympathetic neurons [48].

As shown in Table 4, overall the effects elicited by chemicals grouped in the ‘3-Sim’ mixture (linked to BDNF alterations) are often similar in strength to the mixture of all 6 chemicals, while the mixture ‘3-Diss’ with the three dissimilar chemicals is less potent. Notably, a strict use of the concentration addition approach and the TU model for the identification of synergistic effects in mixtures may not be a suitable strategy to predict mixture effects on some of the DNT endpoints analysed in this study (i.e., % of neuronal cells, neurite outgrowth and BDNF levels), for which single chemical contribution to mixture effects resulted above the threshold (TU > 1). This phenomenon was somehow expected, considering that synaptogenesis, neurite outgrowth and BDNF levels are dynamic, biologically complex processes that change significantly with time of culture, resulting in non-monotonic modulation induced by chemicals under investigation.

Although the use of mathematical modelling did not enable the prediction of synergistic effects induced by mixtures on these DNT endpoints, the comparison between individual chemical BMRs and the observed mixture response suggests induction of combined/potentiated effects, especially upon exposure to the 3-Sim mixture for proportion of neuronal cells, neurite outgrowth and BDNF levels.

More potent effects induced by chemicals grouped as similar (in comparison to dissimilar) were also observed in other studies, e.g., chemicals binding to the colchicine-binding site on tubulin monomers leading to microtubule assembly inhibition, induced concentration addition in CHO-K1 cells, even when individual chemicals were present at (or below) threshold levels [49]. Indeed, chemicals acting through a similar MoA and targeting the same signalling pathway may more potently and irreversibly compromise cellular defence and recovery mechanisms. However, further studies are needed to support this hypothesis.

Notably, the mixture containing all six chemicals resulted less detrimental on neurite length and the number of branch points than the similar MoA mixture (Fig. 7, and Additional file 3: Figure S8E, F), which suggests that the presence of VA in the ‘All’ mixture may induce opposite effects. Indeed, VA as a single chemical was found to stimulate neurite outgrowth (i.e., increase of both neurite length and the number of branch points/neurite, at LOAEC/2-neu) (Additional file 3: Figure S8E, F), as previously shown, for instance, in a murine Alzheimer’s disease model [50], and in human neuroblastoma cells [51].

Additionally, while individually administered CPF, Methyl-Hg and PCB138 at concentrations below LOAEC-neu (LOAEC/2-neu) caused a decrease in neurite number and the proportion of neuronal cells after 14 days of exposure, mixtures elicited opposite (possible synergistic) effects, as shown by an increase of neurites (by ~ 17%) and significant augmentation of neuronal cells (by ~ 55%) (Additional file 3: Figure S8G, H, black curves), especially in the ‘3-Sim’ mixture. The observed increase in the proportion of neurons, characterised by higher numbers of neurites (but shorter and less branched), may contribute to the observed alteration of synaptogenesis (i.e., increased levels and lack of co-localisation of pre- and post-synaptic markers).

These in vitro results are consistent with studies on neurodevelopmental disorders described in children. For instance, an increase of neuronal cell numbers was identified in the prefrontal cortex of autistic children (about 67%) compared with healthy control children [52], and neurons derived from autistic children presented impaired neurite morphology, with shorter and less branched neurites [53, 54]. Moreover, about 80% of the genes that are considered to be high-risk for autism spectrum disorder (ASD) play an important role in early neurodevelopmental functions, in particular neurite outgrowth and synapse formation [55].

The above changes (increased number of neurons, higher number of neurites, and alteration of synaptogenesis) could be linked to the observed increased BDNF levels. Indeed, similar MoA chemicals (BPA/CPF/Lead) upregulated BDNF levels and this increase was even more prominent with all six chemicals together (Fig. 8). BDNF is known to be involved in the promotion of neuronal survival and neuronal protection, modulating neurite outgrowth, excitability and synapse plasticity [56, 57]. Moreover, elevated BDNF levels both in peripheral blood [58] and in the frontal cortex [59] have been described in ASD children and confirmed by recent meta-analyses [60, 61].

Taken altogether, the observed increase in BDNF at the protein level and in the proportion of neuronal cells characterised by shorter and less branched neurites, as well as the alteration in synaptogenesis, suggest that the applied human in vitro model may permit the induction of some ASD-like phenotypic features upon exposure to the ‘3-Sim’ and the ‘All’ mixtures (not observed upon exposure to single chemicals). The correlation between the cellular changes observed in the developing brain of autistic children with the in vitro results obtained in this study may suggest that our approach is a reliable strategy for identifying chemical mixtures with potential to cause DNT effects. This approach is based on a mixed neuronal/astrocytic cultures derived from human iPSCs, which recapitulates key stages of neuronal differentiation, and in vitro assays anchored to CKEs of the DNT AOP network.

Previous DNT studies on MRA have described the combined effects of mixtures accounting for only one class of chemicals, such as PCBs (organotypic co-cultures of developing rat ventral mesencephalon and striatum [62]), or only polybrominated diphenyl ethers (co-culture of mouse cerebellar granule neurons and astrocytes [63]), or mixtures of only metals (perinatally exposed rats and in rat primary astrocytes [64]), highlighting additive or synergistic effects. Others have reported neither additive nor synergistic effects of mixtures with chemicals from different classes, such as Methyl-Hg and PCBs (e.g., [65, 66]), showing no differences in DNT effects comparing the mixtures with the individually tested chemicals. Here, deliberately, we have selected chemicals that represent different classes to more realistically represent real life exposure.

Another interesting observation comes from the comparison of LOAEC concentrations across the applied assays and testing of both single chemicals and mixtures, which suggests that alteration in synaptogenesis seems to be a more sensitive DNT endpoint than neurite outgrowth or changes in BDNF levels. In the case of single chemicals, the LOAEC specific for synaptogenesis (LOAEC-syn) was the lowest for lead (0.007 μM), followed by Methyl-Hg (0.26 μM), PCB138 (5.9 μM), BPA (28.9 μM), CPF (37.1 μM), and VA (420 μM) after acute (3 days) exposure, and equal or in some cases even lower after exposure for 14 days (BPA, 12.74 μM; CPF, 21 μM; lead, 0.007 μM; Methyl-Hg, 0.05 μM; PCB138, 0.06 μM; and VA, 2.1 μM) (see Table 3).

Based on LOAECs specific values for mixtures (Table 4), again synaptogenesis turned out to be the most sensitive DNT endpoint. Indeed, after 3 day exposure to ‘3-Sim’ chemicals, the LOAEC values for synaptogenesis were equal to LOAEC/4-syn (i.e., lead, 0.0018 μM; CPF, 9.28 μM; and BPA, 7.24 μM). However, after 14 days of exposure, LOAEC concentrations were slightly higher, LOAEC/2-syn (i.e., lead, 0.0037 μM; CPF, 10.5 μM and BPA, 6.3 μM), possibly due to the induction of defence mechanisms and adaptive changes during 2 week repeated treatments. The higher sensitivity of synaptogenesis was confirmed also by using mathematical modelling, which enabled the prediction of synergistic effects induced by mixtures on some of the synaptogenesis-related features (Fig. 9, solid arrows). For some of the tested chemicals (i.e., lead, Methyl-Hg and VA), these in vitro concentrations are relevant to human exposure based on concentrations found in human samples; for instance, in cord blood the concentrations of lead has been described in the range of ~ 0.004 and 0.13 μM, for Methyl-Hg between ~ 0.003 and 0.14 μM, and for VA between ~ 27 and 500 μM (Table 1). On the other hand, the concentrations of BPA, CPF and PCB138 found in human samples were lower than those tested in this and other in vitro studies, e.g., BPA [67, 68], CPF [26, 69], and PCB138 [70]. Cord blood concentrations of BPA have been reported to be ~ 0.009 μM and ranging between ~ 0.004 and 0.1 μM in children’s serum (i.e., about 60 times lower than the concentrations used in 14 day treatments). PCB138 has been found in the range of 4-5 × 10^− 4^ μM (i.e., about 100 times lower), and CPF in the range of ~ 7 × 10^− 6^ and 0.013 μM (i.e., at least a 1000 times lower) (Table 1). However, the actual levels of these chemicals reaching the developing brain and their possible accumulation during years of exposure are unknown. A pregnancy-physiologically based pharmacokinetic (P-PBPK) model to predict the toxicokinetic profile of BPA in the foetus during gestational growth has been developed by Sharma and co-authors [71]. Similar models to estimate the absorption, distribution, metabolism and excretion (ADME) of other environmental chemicals and their capacity to reach and accumulate in the developing brain are needed to predict concentration of chemicals in the brain that pass the blood-brain barrier.

To our knowledge, this is the first study reporting on the DNT effects triggered by exposure to mixtures of chemicals belonging to different classes using a human in vitro model and assays (such as synaptogenesis, neurite outgrowth and BDNF levels alteration) anchored to KEs identified in currently available DNT AOPs, and supported by mathematical modelling. These endpoints permitted the evaluation of common KEs identified in DNT AOP network leading to a similar AO (i.e., impairment of learning and memory in children or cognitive deficits) [14]. While individual AOPs are likely to be triggered by chemicals belonging to the same class, assembly of single AOPs into a network (Fig. 1) through interconnected pathways [14], likely represents a more realistic scenario, illustrating that exposure to mixtures of chemicals may trigger simultaneously multiple MIEs but still leading to the same AO (Fig. 1). In this study, the CKEs guided the selection of in vitro assays, allowing a more holistic understanding of the signalling pathways involved in impairment of learning and memory/cognitive deficit (AO). The obtained results confirmed the expected pattern of changes, and these data could be used to update the description of the relevant KERs, to enable their semi-quantitative understanding. The approach described here serves as an important example of how AOP network can be applied for testing not only single chemicals but also chemical mixtures [72]. Mechanistic knowledge built in the underlying AOP network increases scientific confidence in the produced in vitro data, hopefully facilitating their acceptance for regulatory purposes.

We have focused on learning and memory impairment/deficit in cognitive capacity of children since according to recent epidemiological studies this adverse outcome, together with other neurodevelopmental disorders (e.g., attention deficit hyperactivity disorder (ADHD), autism, lower IQ, etc.), is becoming increasingly prevalent, and exposure to environmental chemicals may contribute to the development of these diseases [1, 73]. Furthermore, learning and memory testing is an endpoint required by regulatory DNT studies, currently performed (when triggered) using rodents following the OECD TG 426 [74]. However, according to the EFSA Scientific Opinion [75], learning and memory assessment following the guidelines methodology is too flexible and its sensitivity varies, therefore some effects could remain undetected [76]. Furthermore, a recent consensus among various stakeholders (regulatory bodies, academia and industry) has been reached, arguing that a new testing framework based on alternative approaches is urgently needed to improve and speed up testing of chemicals for their DNT potential [34, 77]. In this context, the obtained data suggest that the applied in vitro approach could be included in Integrated Approaches to Testing and Assessment (IATA) for different regulatory purposes, as recently suggested [16]. The battery of in vitro assays applied in this study (i.e., synaptogenesis, neurite outgrowth and BDNF levels) and the use of human neuronal in vitro models (avoiding the need to extrapolate between different species) would be suitable for an initial screening to identify chemicals with potential to trigger DNT effects, particularly those associated with learning and memory impairment in children.

## Conclusions

The obtained results suggest that individual chemicals, representing different classes (industrial chemicals, pesticides, EDs, drugs and POPs) at non-cytotoxic and very low cytotoxic concentrations (relevant to human exposure), may become developmental neurotoxicants in a mixture. The applied in vitro model based on human cells and assays anchored to key events of the DNT AOPs permits better mechanistic understanding of toxicity pathways involved in *Learning and memory impairment/Cognitive damage* in children. Such an in vitro approach increases scientific confidence in the obtained data and could be incorporated in the current OECD DNT TG 426 to improve and speed up chemicals evaluation to identify especially those linked to children cognitive damage, the most prevalent neurodevelopmental disorder.

## Supplementary information

**Additional file 1: Table S1.** Chemicals acting through similar MoA (alterations of BDNF levels): summary of their effects and mode of action (MoA) based on literature review, along with epidemiological studies describing chemical concentrations found in human biological samples. **Table S2.** Chemicals acting through dissimilar MoAs (not directly linked to alterations of BDNF levels): summary of their effects and mode of action (MoA) based on literature review, along with epidemiological studies describing chemical concentrations found in human biological samples.

**Additional file 2: Figure S1.** Effects elicited by Bisphenol A (BPA); quantitative evaluation of immunocytochemistry using HCI (Cellomics platform). IMR90-derived NSCs were differentiated for 7 DIV and treated for either 3 or 14 days with three different concentrations of BPA (0.29 μM, IC20/100, white bars; 12.74 μM, IC5, grey bars; 28.96 μM, IC20, black bars) in comparison to solvent control (0.1% DMSO, Ctr) at the respective time point. Analysis of BPA effects on: (A) total (i.e., cell body and neurites) and in neurites only expression of SYP (pre-synaptic) and PSD95 (post-synaptic proteins (MAP2 staining was used as a marker of neurites), and the number of overlapping SYP/PSD95 spots (synapses) in the neurites; (B) neurite length and branch points per neurite; (C) total BDNF protein levels and BDNF expression ratio, comparing neurite to cell body. Data are represented as mean ± S.E.M. of 3–4 biological replicates. **Figure S2.** Effects elicited by Chlorpyrifos (CPF); quantitative evaluation of immunocytochemistry using HCI (Cellomics platform). IMR90-derived NSCs were differentiated for 7 DIV and treated for either 3 or 14 days with three different concentrations of CPF (0.37 μM, IC20/100, white bars; 21.01 μM, IC5, grey bars; 37.10 μM, IC20, black bars) in comparison to solvent control (0.1% DMSO, Ctr) at the respective time point. Analysis of CPF effects on: (A) total (i.e., cell body and neurites) and in neurites only expression of SYP (pre-synaptic) and PSD95 (post-synaptic proteins (MAP2 staining was used as a marker of neurites), and the number of overlapping SYP/PSD95 spots (synapses) in the neurites; (B) neurite length and branch points per neurite; (C) total BDNF protein levels and BDNF expression ratio, comparing neurite to cell body. Data are represented as mean ± S.E.M. of 3–4 biological replicates. **Figure S3.** Effects elicited by Lead(II) chloride (Lead); quantitative evaluation of immunocytochemistry using HCI (Cellomics platform). IMR90-derived NSCs were differentiated for 7 DIV and treated for either 3 or 14 days with three different concentrations of Lead (0.0073 μM, IC20/100, white bars; 0.17 μM, IC5, grey bars; 0.73 μM, IC20, black bars) in comparison to solvent control (0.1% DMSO, Ctr) at the respective time point. Analysis of Lead effects on: (A) total (i.e., cell body and neurites) and in neurites only expression of SYP (pre-synaptic) and PSD95 (post-synaptic proteins (MAP2 staining was used as a marker of neurites), and the number of overlapping SYP/PSD95 spots (synapses) in the neurites; (B) neurite length and branch points per neurite; (C) total BDNF protein levels and BDNF expression ratio, comparing neurite to cell body. Data are represented as mean ± S.E.M. of 3–4 biological replicates. **Figure S4.** Effects elicited by Methylmercury(II) chloride (Methyl-Hg); quantitative evaluation of immunocytochemistry using HCI (Cellomics platform). IMR90-derived NSCs were differentiated for 7 DIV and treated for either 3 or 14 days with three different concentrations of Methyl-Hg (0.0013 μM, IC20/100, white bars; 0.05 μM, IC5, grey bars; 0.13 μM, IC20, black bars) in comparison to solvent control (0.1% DMSO, Ctr) at the respective time point. Analysis of Methyl-Hg effects on: (A) total (i.e., cell body and neurites) and in neurites only expression of SYP (pre-synaptic) and PSD95 (post-synaptic proteins (MAP2 staining was used as a marker of neurites), and the number of overlapping SYP/PSD95 spots (synapses) in the neurites; (B) neurite length and branch points per neurite; (C) total BDNF protein levels and BDNF expression ratio, comparing neurite to cell body. Data are represented as mean ± S.E.M. of 3–4 biological replicates. **Figure S5.** Effects elicited by PCB138; quantitative evaluation of immunocytochemistry using HCI (Cellomics platform). IMR90-derived NSCs were differentiated for 7 DIV and treated for either 3 or 14 days with three different concentrations of PCB138 (0.0593 μM, IC20/100, white bars; 3.53 μM, IC5, grey bars; 5.93 μM, IC20, black bars) in comparison to solvent control (0.1% DMSO, Ctr) at the respective time point. Analysis of PCB138 effects on: (A) total (i.e., cell body and neurites) and in neurites only expression of SYP (pre-synaptic) and PSD95 (post-synaptic proteins (MAP2 staining was used as a marker of neurites), and the number of overlapping SYP/PSD95 spots (synapses) in the neurites; (B) neurite length and branch points per neurite; (C) total BDNF protein levels and BDNF expression ratio, comparing neurite to cell body. Data are represented as mean ± S.E.M. of 3–4 biological replicates. **Figure S6.** Effects elicited by Valproic acid (VA); quantitative evaluation of immunocytochemistry using HCI (Cellomics platform). IMR90-derived NSCs were differentiated for 7 DIV and treated for either 3 or 14 days with three different concentrations of VA (2.1 μM, IC20/100, white bars; 70 μM, IC5, grey bars; 210 μM, IC20, black bars) in comparison to solvent control (0.1% DMSO, Ctr) at the respective time point. Analysis of VA effects on: (A) total (i.e., cell body and neurites) and in neurites only expression of SYP (pre-synaptic) and PSD95 (post-synaptic proteins (MAP2 staining was used as a marker of neurites), and the number of overlapping SYP/PSD95 spots (synapses) in the neurites; (B) neurite length and branch points per neurite; (C) total BDNF protein levels and BDNF expression ratio, comparing neurite to cell body. Data are represented as mean ± S.E.M. of 3–4 biological replicates.

**Additional file 3: Figure S7.** Single chemicals and mixtures effects on synaptogenesis. HiPSC-derived NSCs were differentiated for 7 DIV, and then treated for either 3 days (A-C) or 14 days (D-F) with single chemicals (BPA, CPF, Lead, Methyl-Hg, PCB138 and VA) or three types of mixtures: (i) a mixture with the 3 similar MoA chemicals (‘3-Sim’), (ii) a mixture with the 3 dissimilar MoA chemicals (‘3-Diss’), and (iii) a mixture with all 6 chemicals (‘All’). Graphs show: (A, D) total PSD95 protein levels; (B, E) total SYP protein levels, and (C, F) the number or overlapping SYP and PSD95 spots at the levels of nerites (i.e., SYP/PSD95 co-localization, synapses). All samples were normalized to solvent control (0.1% DMSO, Ctr) at the respective time point. LOAEC-syn (red curves) and their serial dilutions (respectively black (1:2) and blue (1:4) curves) were tested as indicated in Table 3 (reporting concentrations tested for each individual chemical). Data are represented as mean ± S.E.M. of 3–4 biological replicates. **Figure S8.** Single chemicals and mixtures effects on neurite outgrowth. HiPSC-derived NSCs were differentiated for 7 DIV, and then treated for either 3 days (A-D) or 14 days (E-G) with single chemicals (BPA, CPF, Lead, Methyl-Hg, PCB138 and VA) or three types of mixtures: (i) a mixture with the 3 similar MoA chemicals (‘3-Sim’), (ii) a mixture with the 3 dissimilar MoA chemicals (‘3-Diss’), and (iii) a mixture with all 6 chemicals (‘All’). Graphs show: (A, E) neurite length, (B, F) the number of branch points per neurite, (C, G) the number of neurites per neuron, and (D, H) the percentage of β-III-Tubulin+ cells. All samples were normalized to solvent control (0.1% DMSO, Ctr) at the respective time point. LOAEC-neu (red curves) and their serial dilutions (respectively black (1:2), blue (1:4) and light blue (1:8) curves) were tested as indicated in Table 3 (reporting concentrations tested for each individual chemical). Data are represented as mean ± S.E.M. of 3–4 biological replicates. **Figure S9.** Effects of chlorpyrifos (CPF) and valproic acid (VA) withdrawal from mixtures on neurite outgrowth. HiPSC-derived NSCs were differentiated for 7 DIV, and then treated for 14 days with: (i) the ‘3-Sim’ mixture (BPA, Lead and CPF) and the same mixture without CPF, (ii) the ‘3-Dissim’ mixture (Methyl-Hg, PCB138 and VA) and the same mixture without VA, (iii) the ‘All’ mixture (BPA, CPF, Lead, Methyl-Hg, PCB138, and VA) and the same mixture excluding CPF and VA. (A) Representative pictures at 10x magnification (β-III-Tubulin, in red). (B-E) Graphs show neurite outgrowth after 14 day treatment with LOAEC/2-neu (black bars), and LOAEC/4-neu concentrations (blue bars). Graphs show total live (non-pyknotic) cell numbers (B, based on DAPI staining), neurite length (C), the number of branch points per neurite (D), and the number of neurites per neuron (E) analysed upon treatments with LOAEC/2-neu and LOAEC/4-neu concentrations as indicated in Table 3 (reporting concentrations tested for each individual chemical). Values were normalised to the corresponding complete mixtures, and data are represented as mean ± S.E.M. of 3 biological replicates. Statistical significance was assessed by unpaired t-test, comparing each differential mixture vs the original mixture (e.g., ‘3-Sim’ vs ‘3-Sim (−CPF)’). *p* < 0.05, ** *p* < 0.01, *** *p* < 0.001.

**Additional file 4: Figure S10.** Single chemical curves. Each panel represents the modeled response of single chemicals (i.e., A, BPA; B, CPF; C, Lead; D, Methyl-Hg; E, PCB138, and F, VA) for all the selected DNT endpoints. To calculate the single chemical dose response effects for all the selected DNT endpoints the following concentrations were tested: BPA (8.5, 12.7, 19.1, 28.7, 43.0, 60.2 μM), CPF (18.5, 21.2, 24.4, 28.1, 32.3, 37.1 μM), Lead (0.001, 0.007, 0.037, 0.18, 0.91, 1.27, 1.46, 1.68, 1.93, 2.22, 2.67 μM), Methyl-Hg (0.03, 0.05, 0.09, 0.16, 0.29, 0.33 μM), PCB138 (0.01, 0.06, 0.25, 1.05, 4.39, 9.41 μM), and VA (0.5, 2.1, 8.4, 33.6, 134.4, 537 μM). The effect was estimated according to seven different mathematical models (i.e., Hill, Power, Linear, Polynomial 2, Exponential 2, Exponential 3, Exponential 4, and Exponential 5) by using the BMDExpress.2 open access software (https://github.com/auerbachs/BMDExpress-2/wiki). The best-fitting curve across the range of concentration tested is represented in the figure as percentage of response compared to the solvent control (0.1% DMSO). **Table S3.** BMDs synaptogenesis. The table lists the best fitting model selected for the analysis of synaptogenesis performed by BMDExpress, according to the lowest Akaike information criterion and higher fit *P* value. The value of the calculated BMD_5_ for each chemical is also reported, including the respective BMDL and BMDU. **Table S4.** BMDs % neurons and neurite outgrowth. The table lists the best fitting models selected for the analysis of neuronal cell (β-III-tubulin^+^) percentage and neurite outgrowth-related parameters performed by BMDExpress, according to the lowest Akaike information criterion and higher fit P value. The value of the calculated BMD_5_ for each chemical is also reported, including the respective BMDL and BMDU. **Table S5.** BMDs BNDF levels. The table lists the best fitting models selected for the analysis of BDNF levels performed by BMDExpress, according to the lowest Akaike information criterion and higher fit P value. The value of the calculated BMD_5_ for each chemical is also reported, including the respective BMDL and BMDU.

## Data Availability

The datasets used and/or analysed during the current study are available from the corresponding author on reasonable request.
