# Supplementary material for: Assessment of developmental neurotoxicity induced by chemical mixtures using an adverse outcome pathway concept
Source: Environ Health. 2020 Feb 24;19:23. doi: 10.1186/s12940-020-00578-x (PMC7038628; doi:10.1186/s12940-020-00578-x)
Supplement: Supplementary file 1 — Additional file 1: Table S1. Chemicals acting through similar MoA (alterations of BDNF levels): summary of their effects and mode of action (MoA) based on literature review, along with epidemiological studies describing chemical concentrations found in human biological samples. Table S2. Chemicals acting through dissimilar MoAs (not directly linked to alterations of BDNF levels): summary of their effects and mode of action (MoA) based on literature review, along with epidemiological studies describing chemical concentrations found in human biological samples. [file 12940_2020_578_MOESM1_ESM.docx]

| **Table S1: Chemicals acting through similar MoA (alterations of BDNF levels)** | | | |
| --- | --- | --- | --- |
| **Chemical name** | **Class** | **MoAs and effects** | **Epidemiological data: human biological samples and concentrations** |
| Lead(II) chloride | Metal | Decreased CREB phosphorylation in rat cerebral cortex at 14 PND and also in both cortex and hippocampus at PND 50, altering NMDAR subtypes [[1-3](#_ENREF_1)].  Decreased both the both the cellular expression of proBDNF and the release of BDNF in primary hippocampal neurons, impairing vesicular release and synaptogenesis [[4](#_ENREF_4)];  Decreased dendritic proBDNF protein levels throughout the length of the dendrites; caused impairment of BDNF vesicle transport to sites of release in dendritic spines in primary hippocampal neurons, and decreased phosphorylation of Synapsin I [[5](#_ENREF_5)];  Other effects:  The sequestration of lead at the level of the choroid plexus undermines brain growth and affects learning and cognitive functions of CNS [[6](#_ENREF_6)];  Decreased the mRNA level of GFAP, interfered with astrocyte production, and modulated synaptic efficiency in the developing rat brain [[7](#_ENREF_7)], and in primary cultures of rat cerebellar granule cells [[8](#_ENREF_8)];  Activated MAPK signaling in treated rat primary astrocytes; induced apoptosis involved activation of ERK1/2 and JNK1/2, increased intracellular Ca2+, and ROS generation in rat primary astrocytes [[9](#_ENREF_9)] and in C6 glioma cells [[10](#_ENREF_10)];  Acute and chronic exposure to Pb affects protein kinase C (PKC) and the N-methyl-D-aspartate subtype of glutamate receptor (NMDAR) [[11](#_ENREF_11)];  Elevated oxidative stress in cortical synapse-specific mitochondria in developing rats [[12](#_ENREF_12)]. | Children hair:   - 0.63 ± 0.50 (0.18–1.7) μg/g [[13](#_ENREF_13)] - 6.65 ppm [[14-16](#_ENREF_14)] - 0.5 mg/kg [[17](#_ENREF_17)] - 5 - 22 ppm [[18](#_ENREF_18)]   Children urine:   - 1.3 μg/g creatinine [[19](#_ENREF_19)]   Cord blood:   - 11.41 μg/L (range 0.16–43.22) [[20](#_ENREF_20)] - 6.83–220.8 μg/L [[21](#_ENREF_21)] - (prenatal lead) 11.2 ± 0.9 μg/dL [[22](#_ENREF_22)] - 1.09 ±.88 μg/dL (range 0.15–7.45) [[23](#_ENREF_23)] - 8.30 μg/L (range 7.94-8.68) [[24](#_ENREF_24)]   Children blood:   - 8.9 μg/dL [[25](#_ENREF_25)] - 10 μg/dL [[26](#_ENREF_26)] - 5.1 ± 2.6 μg/dL (12 months) and 5.0 ± 2.9 μg/dL (24 months) [[27](#_ENREF_27)] - 1.73 μg/dL (SD = 0.8; median = 1.55; range = 0.42–4.91) [[28](#_ENREF_28)] - 1.71 μg/dL (median 1.5, range 0.44–10.2) [[29](#_ENREF_29)] - 4.53 μg/dL (range 4.27–4.79) [[30](#_ENREF_30)]   Maternal blood:   - 1.36 μg/dL (range 0.26–9.10) [[31](#_ENREF_31)] - 4.51 μg/dL (range 4.15–4.90) [[30](#_ENREF_30)] |
| Chlorpyrifos | Pesticide | Inhibits acetylcholinesterase (AChE);  Acts on BDNF at low concentrations during development;  Increased Bdnf expression in the CA1 region of rat hippocampus, increasing the levels of neuropeptides (CRHBP (Corticotropin releasing hormone binding protein) and NPY (Neuropeptide Y)) in the CA1 region of the hippocampus [[32](#_ENREF_32)];  Increased both BDNF gene and protein levels in adult zebrafish brain tissues, perturbing brain morphology [[33](#_ENREF_33)];  Increased Bdnf expression in differentiating PC12 cells in vitro [[34](#_ENREF_34)], and decreased cholinergic presynaptic markers (i.e., choline acetyltransferase activity (ChAT) and [3H]hemicholinium-3 binding (HC-3) in the hippocampus, midbrain, striatum, brainstem and cerebral cortex in the juvenile and young adult rats) [[35](#_ENREF_35)];  Increased both Bdnf gene (at PND20 and PND28 in the hippocampus and cerebral cortex) and protein levels (only in the hippocampus at PND20), reducing cholinesterase activity in young rats [[36](#_ENREF_36)];  Via non-cholinesterase mechanisms: CPF increased CREB phosphorylation in primary cortical and hippocampal neurons (but not astrocytes) [[37](#_ENREF_37)]; and inhibited neurite outgrowth in PC12 cells [[38](#_ENREF_38)];  Other effects:  Induced long-term dopaminergic neuronal damage in the substantia nigra mediated by the activation of inflammatory response, via NF-κB p65 (NF-κB is known to activate Bdnf expression) and p38 MAPK pathways (MAPK is known to be activated by BDNF) in the nigrostriatal system of young adult rats [[39](#_ENREF_39)];  Inhibited axonal outgrowth in embryonic rat dorsal root ganglia neurons (by inhibiting the morphogenic, rather than enzymatic, activity of AChE) [[40](#_ENREF_40)], and in primary cultures of embryonic rat sympathetic neurons (in a way that is independent of AChE inhibition) [[41](#_ENREF_41)];  Altered mitochondria dynamics and movement in rat cortical neurons [[42](#_ENREF_42)];  Caused long-lasting changes in spatial learning and memory formation in juvenile and adult rats [[43](#_ENREF_43)];  Prenatal exposure was associated with structural abnormalities that included thinning of the cerebral cortex as shown by MRI of the brain [[22](#_ENREF_22), [44](#_ENREF_44)]. | Breast milk:   - (CPF-ethyl) 4.61 ± 11.14 ng/g lipid (95 percentile: 17.06) [[45](#_ENREF_45)] - 20.5 pg/g milk before pasteurization (range: 4.2–54. 6) [[46](#_ENREF_46)]   Children urine:   - (within spray season): 3.8 μg/g creatinine (geometric mean) [[47](#_ENREF_47)] - for 3,5,6-Trichloro-2-pyridinol (TCPy, a CPF metabolite):   22.6 ng/mL [[48](#_ENREF_48)]  21.5 μg/g creatinine [[49](#_ENREF_49)]  3.36 ng/mL [[50](#_ENREF_50)]  1.12 ng/mL [[51](#_ENREF_51)]  5.3 ng/ml (max value 104 ng/ml) [[52](#_ENREF_52)]  1.4 μg/L (range: 0.7–3.1) [[53](#_ENREF_53)]  Cord plasma:   - > 6.17 pg/g plasma (in high exposed children) [[54](#_ENREF_54)] - at delivery: 4.65 ng/mL (99th percentile) [[55](#_ENREF_55)]   Cord blood:   - highly exposed: ≥ 4.39 pg/g [[22](#_ENREF_22)] - highly exposed: > 6.17 pg/g plasma [[54](#_ENREF_54)] - 3.17 ± 4.61 (range 0.09–32) pg/g [[23](#_ENREF_23)] - 2.5 pg/g (95^th^ percentile) (for TCPy) [[56](#_ENREF_56)]   Maternal blood:   - 2.5 pg/g (95^th^ percentile) (for TCPy) [[56](#_ENREF_56)]   Maternal urine:   - 3.5-7.8 μg/L (95^th^ percentile, in different prenatal weeks) (for TCPy) [[56](#_ENREF_56)] |
| Bisphenol A | Organic compound  (ED, estrogenic) | Increased DNA methylation of the putative 5' promoter region of Bdnf in the hippocampus of female rat offspring [[57](#_ENREF_57)];  Increased DNA methylation in the transcriptionally relevant region of the Bdnf gene (Bdnf IV, CREB-binding site) in the hippocampus and blood of BALB/c mice (lower Bdnf gene expression); induced hypermethylation in human cord blood (in males) [[58](#_ENREF_58)];  Decreased p-CREB and BDNF levels in the hippocampus of rat male offspring; this was associated with impairment of object recognition memory, and decreased Akt activation [[59](#_ENREF_59)];  Altered synaptic structure, widening synaptic cleft, thinning postsynaptic density, and lowering synaptic vesicles; decreased the mRNA and protein expressions of SYP, PSD95, GluR1 and NMDAR1 in the hippocampus of prenatally exposed rat male offspring [[60](#_ENREF_60), [61](#_ENREF_61)];  Decreased Bdnf gene expression levels in embryonic mouse hypothalamic cells treated with 200 μM BPA [[62](#_ENREF_62)];  Decreased phosphorylation of CREB and inhibited neurite extension, and ERK1/2 MAPK in PC12 cells [[63](#_ENREF_63)];  Decreased BDNF, p-CREB (and p-ERK) protein levels in the hippocampus of female offspring mice [[64](#_ENREF_64)];  Decreased Bdnf (and c-fos) gene expression in a dose dependent manner, and increased Ca^2+^ uptake in mouse cerebellar granule cells [[65](#_ENREF_65)];  Decreased working memory accuracy and the number of excitatory synaptic inputs on dendritic spines of pyramidal neurons in prefrontal cortex and hippocampus [[66](#_ENREF_66)], and decreased midbrain dopamine neurons and hippocampal spine synapses (in non-human primates) [[67](#_ENREF_67)];  Up-regulated synapsin I and PSD95 and NMDA receptor NR2B in the hippocampus of estrogen-deprived female mice [[68](#_ENREF_68)];  Interfered with gonadal steroid-induced synaptogenesis (i.e., inhibits by 70-100% the increase of hippocampal and prefrontal spine synapses induced by estrogens and androgens), resulting in loss of spine synapses, in both rats and non-human primates [[69](#_ENREF_69)];  Other effects:  Increased dopamine D2 receptor binding in the caudate putamen but decreased dopamine transporter binding; decreased NMDA receptor binding in the frontal cortex and CA1, CA3, and DG of the hippocampus in mice exposed during prenatal and postnatal periods [[70](#_ENREF_70)];  Mediated increased β-catenin phosphorylation, leading to a decrease of GSK-3β levels and β-catenin nuclear translocation in rat pups and in primary hippocampal NSCs [[71](#_ENREF_71)]. | Breast milk:   - 6.5 ng/g lipid (range 0.80–42.2) [[46](#_ENREF_46)] - 0.3-0.7 μg/L [[72](#_ENREF_72)]   Children urine:   - 4.76 μg/L (range 2.77-9.03) [[73](#_ENREF_73)] - 3.2 ± 5.0 μg/L, 4.9 ± 10.8 μg/g creatinine [[74](#_ENREF_74)] - 2.76 ng/mL [[75](#_ENREF_75)] - 232.60 ± 126.05 μg/L (geometric mean (GM ± SD) [[76](#_ENREF_76)] - 0.76 μg/g creatinine [[77](#_ENREF_77)] - low levels: 4.09 ± 1.75, high levels: 501.59 ± 237.86 μg/g creatinine [[78](#_ENREF_78)] - 1.08 μg/L [[79](#_ENREF_79)] - 5.1 ±13.3 μg/g creatinine [[80](#_ENREF_80)] - 11.84 ±3.35 ng/ml (BPA glucoronide, BPAG) [[59](#_ENREF_59)] - 1.3 μg/L (range 0.1-420) [[81](#_ENREF_81)] - 3.66 ± 3.23 ng/mL [[82](#_ENREF_82)]   Children serum:   - 0.85-22.5 ng/mL [[83](#_ENREF_83)] - 14.44 ± 6.88 ng/mL [[82](#_ENREF_82)]   Maternal urine:   - 2.6 μg/g creatinine [[84](#_ENREF_84)] - 2.0 ± 2.1 μg/g creatinine [[74](#_ENREF_74)] - 2.7 μg/g creatinine [[85](#_ENREF_85)] - 0.69 μg/L (range 0.64-0.74) [[24](#_ENREF_24)] - 0.70 µg/L (range 0.63–0.79) [[30](#_ENREF_30)] - 0.6-31.9 μg/L [[72](#_ENREF_72)]   Maternal serum:   - 0.3-0.8 μg/L [[72](#_ENREF_72)] |

| **Table S2: Chemicals acting through dissimilar MoAs (not directly linked to alterations of BDNF levels)** | | | |
| --- | --- | --- | --- |
| **Chemical name** | **Class** | **MoAs and effects** | **Epidemiological data: human biological samples and concentrations** |
| Methylmercury(II) chloride | Metal | Caused disassembly of microtubules with secondary collapse of vimentin filaments in epithelial kidney cells Ptk2 [[86](#_ENREF_86)];  Found to inhibit astrocytic glutamate uptake, increase glutamate release, lead to elevated extracellular glutamate levels, which causes overactivation of NMDA-type glutamate receptor, leading to increased influx of Ca^2+^ into postsynaptic neurons, and activation of cell death pathways (reviewed in [[87](#_ENREF_87)]);  Found to disrupt mitochondrial electron transport chain, leading to increased formation of ROS; deplete GSH by forming MeHg–GSH complex (MeHg-SG). This adduct is excreted into extracellular space through multidrug-resistance-associated protein, which is regulated by Nrf2 (reviewed in [[87](#_ENREF_87)]);  Has very high affinity for sulfur-containing anions, particularly the thiol (-SH) groups on the amino acid Cys and hence in proteins containing cysteine, forming a covalent bond [[87](#_ENREF_87), [88](#_ENREF_88)];  Interacts with selenol groups [[89](#_ENREF_89)];  Japanese pregnant women who consumed the MeHg-contaminated fish gave birth to infants with severe developmental disabilities, including cerebral palsy, mental retardation and seizures [[90](#_ENREF_90), [91](#_ENREF_91)];  In children, causes neuropsychological dysfunctions in language, attention, and memory domains [[92](#_ENREF_92)];  Modulates Keap1/Nrf2 signaling: increases heme-oxigenase-1 (HO-1), NQO1 NAD(P)H quinone dehydrogenase (NQO1), glutamate-cysteine ligase catalytic subunit (GCLC), and γ-Glutamylcysteine synthetase (γGCS) genes and proteins in several cells types (reviewed in [[93](#_ENREF_93)]);  Covalently modified PTEN, inhibiting its catalytic activity, thereby phosphorylating Akt through Thr473 and its downstream transcription factor CREB, leading to up-regulation of anti-apoptotic protein Bcl-2 in SH-SY5Y cells [[94](#_ENREF_94)];  Suppressed tropomyosin receptor kinase A (TrkA) pathway, inhibiting neurite extension, and causing cell death in PC12 cell-derived neurons [[95](#_ENREF_95)].  While it can inhibit neurotrophin signalling, it does not induce conformational changes of BDNF [[96](#_ENREF_96)]. | Breast milk:   - 0.376 μg/kg total mercury (THg) [[97](#_ENREF_97)]   Children hair:   - 0.33 ± 1.01 (0.03–4.4) μg/g [[13](#_ENREF_13)] - 0.25 mg/kg [[17](#_ENREF_17)] - 0.99 ppm [[14-16](#_ENREF_14)] - 0.12 μg/g [[98](#_ENREF_98)] - 0.61 μg/L (range 0.02 - 5.76) [[99](#_ENREF_99)] - 1.18 μg/g (range 0.06-9.70) [[100](#_ENREF_100)]   Cord blood:   - 12.17 µg/L (range 1.53–64.87 µg/L) [[20](#_ENREF_20)] - 15.7 ng/g (range 2.7-96.1) (total Hg) [[101](#_ENREF_101)] - 4.65 μg/L (range 0.77 -21.06) [[99](#_ENREF_99)] - 34.76 ng/mL [[102](#_ENREF_102)] - 0.70 μg/L (median) [[103](#_ENREF_103)] - 0.504 μg/L (MeHg), and 0.949 μg/L total mercury (tHg) [[97](#_ENREF_97)] - 34.48 ± 20.46 μg/L [[104](#_ENREF_104)] - 5.6-39.3 μg/L (tHg) [[105](#_ENREF_105)]   Maternal hair:   - 0.40 μg/g (range 0.37-0.42) [[24](#_ENREF_24)] - < 10 μg/g (50 nmol/g) [[92](#_ENREF_92)] - 0.2 μg/g [[98](#_ENREF_98)] - 0.75 μg/L (range 0.14 - 3.88) [[99](#_ENREF_99)] - 1.0-5.8 μg/g (tHg) (Llop et al. 2017)   Children blood:   - 2.33 μg/L (range 0.21 - 9.97) [[99](#_ENREF_99)] - 12.7 nmol/L (nM) [[106](#_ENREF_106)] - 6.81 ± 6.22 μg/L (tHg) [[107](#_ENREF_107)] - 1.46 ± 0.90 μg/L (range 0.09 to 9.97) (in dry bloodspots) [[108](#_ENREF_108)]   Maternal blood:   - 3.07 μg/L (range 0.53 -19.96) [[99](#_ENREF_99)] - 18.36 ng/mL [[102](#_ENREF_102)] - 1.03 μg/L [[109](#_ENREF_109)] |
| Valproic acid | (antiepileptic) drug  (ED, antiandrogenic) | Histone deacetylase (HDAC) inhibitor;  Known to induce autistic-like features (i.e., lower sensitivity to pain and higher sensitivity to non-painful stimuli, diminished acoustic prepulse inhibition, locomotor and repetitive/stereotypic-like hyperactivity and lower exploratory activity, reduced number of social behaviors and increased latency to social behaviors) in prenatally exposed rats [[110](#_ENREF_110), [111](#_ENREF_111)];  Increased Nanog and Oct4 gene expression, reduced Pax6, impacting neural development in human embryonic stem cells undergoing differentiation [[112](#_ENREF_112)];  Caused spinal nerve defects in prenatally exposed mouse embryos [[113](#_ENREF_113)];  Blocked voltage-dependent sodium channels, preventing NMDA antagonist neurotoxicity in adult female rats [[114](#_ENREF_114)];  Increased GABA brain levels, and stimulated GABA neurogenesis in rat forebrain stem cells [[115](#_ENREF_115)];  Known as a non-steroidal antagonist for human androgen receptor and progesterone receptor, but not estrogen receptor [[116](#_ENREF_116)],  Potent aromatase inhibitor [[117](#_ENREF_117)];  Decreased neurite outgrowth in murine neural embryonic stem cells [[118](#_ENREF_118)];  Increased neurite extension by downregulating plasminogen activator inhibitor-1 in astrocytes, upregulating the overall activity of tissue plasminogen activator (regulator of neuritogenesis and extension) in neurons and astrocytes [[1](#_ENREF_1)]. | Cord blood:   - 50 - 75 μg/ml (sodium valproate) [[119](#_ENREF_119)] - 5.4 - 72.1 mg/L (cord serum) [[120](#_ENREF_120)] - 48.60 ± 17.12 μg/ml (total), 3.87 ± 4.22 μg/ml (free, unbound) [[121](#_ENREF_121)]   Breast milk:   - 2.0-3.5 μg/ml (sodium valproate) [[119](#_ENREF_119)]   Maternal blood:   - 48 - 85 μg/ml (in late pregnancy) (sodium valproate) [[119](#_ENREF_119)] - 5.3 - 59.5 mg/L [[120](#_ENREF_120)] - 427 ± 97 μmol/L (total and unbound concentrations in serum) [[122](#_ENREF_122)] |
| PCB-138 | POP | One of the most prevalent PCB congeners found in humans;  in prenatally exposed female rat pups (a mix of three PCBs including PCB138):   - reduced serum progesterone levels, - reduced Pdyn (prodynorphin, or proenkephalin B) gene - increased Ahr (Aryl hydrocarbon receptor) and Arnt (Aryl hydrocarbon nuclear translocator) genes - increased Cyp 19a1 (Cytochrome P450, 19a1 aromatase) and Cyp 1b1 (Cytochrome P450, 1b1) genes - increased Esr1 (Estrogen receptor alpha) gene - increased Gabbr1 (GABA-B receptor 1) gene - increased Grin 2c (NMDAR subunit 2c) gene - increased Vdr (Vitamin D receptor) gene   [[123](#_ENREF_123)];  in prenatally exposed male rat pups (a mix of three PCBs including PCB138):   - reduced serum testosterone levels, - reduced Gabbr2 (GABA-B receptor 2) and Gabbr1 (GABA-B receptor 1) genes - decreased Ahr (Aryl hydrocarbon receptor) and Arnt (Aryl hydrocarbon nuclear translocator) genes - increased Cyp 1b1 (Cytochrome P450, 1b1) gene - decreased Grin2a (NMDAR subunit 2a), Gria2 (GluR2), Grik 2 (Kainate 2 receptor), Igf1r (Insulin-like growth factor 1 receptor) - decreased Slc17a1 (Vesicular glutamate transporter 1) gene - increased Tgfa (Transforming growth factor alpha)   [[123](#_ENREF_123)];  Reduced amount of NR1 subunit of NMDA receptors in the cerebellum, impairing learning in prenatally exposed rats [[124](#_ENREF_124)]  Decreased neurophyschological functions in prenatally exposed children (i.e., longer response times and lower scores on the Tower of London) [[125](#_ENREF_125)];  impacted neurological and cognitive development of children up to school-age [[126](#_ENREF_126)];  Interfered with cholinergic nicotinic receptors in the cerebral cortex, affected learning and memory functions in prenatally exposed mice [[127](#_ENREF_127)]. | Breast milk:   - 50.82 ± 22.92 ng/g lipid [[45](#_ENREF_45)] - 6.0 ng/g lipid (range 2.1–16.5) [[46](#_ENREF_46)] - 427 ng/g lipid (considering Σ PCB 138, 153 and 180) [[128](#_ENREF_128)] - 415 ng/g lipid (considering Σ PCB 138, 153 and 180) [[129](#_ENREF_129)] - 404 ng/g lipid (considering Σ PCB 138, 153 and 180) [[130](#_ENREF_130)]   Cord plasma:   - 0.14 ng/ml [[131](#_ENREF_131)] - 0.55 ng/ml (considering Σ PCB 138, 153 and 180) [[128](#_ENREF_128)] - 0.39 ng/ml (considering Σ PCB 138, 153 and 180) [[130](#_ENREF_130)]   Children blood:   - 0.18 ng/ml [[131](#_ENREF_131)]   Maternal blood:   - 11.13 ng/g lipid (range 10.33-11.94) (plasma) [[24](#_ENREF_24)] - 68.7 ng/g lipid (range 48.9–86.2) (PCB-138); 46.0 ng/g fresh weight (31–66) (3’-OH-PCB-138) (serum) [[132](#_ENREF_132)] - 1.22 ng/ml (considering Σ PCB 138, 153 and 180) [[129](#_ENREF_129), [130](#_ENREF_130)]   For a systematic review of studies describing human PCB levels, see [[133](#_ENREF_133)]. |

**References cited in Tables S1 and S2:**

1. Cho KS, Kwon KJ, Choi CS, Jeon SJ, Kim KC, Park JH, Ko HM, Lee SH, Cheong JH, Ryu JH *et al*: Valproic acid induces astrocyte-dependent neurite outgrowth from cultured rat primary cortical neuron via modulation of tPA/PAI-1 activity. *Glia* 2013, 61(5):694-709.

2. Toscano CD, Hashemzadeh-Gargari H, McGlothan JL, Guilarte TR: Developmental Pb2+ exposure alters NMDAR subtypes and reduces CREB phosphorylation in the rat brain. *Brain Res Dev Brain Res* 2002, 139(2):217-226.

3. Toscano CD, McGlothan JL, Guilarte TR: Lead exposure alters cyclic-AMP response element binding protein phosphorylation and binding activity in the developing rat brain. *Brain Res Dev Brain Res* 2003, 145(2):219-228.

4. Neal AP, Stansfield KH, Worley PF, Thompson RE, Guilarte TR: Lead exposure during synaptogenesis alters vesicular proteins and impairs vesicular release: potential role of NMDA receptor-dependent BDNF signaling. *Toxicol Sci* 2010, 116(1):249-263.

5. Stansfield KH, Pilsner JR, Lu Q, Wright RO, Guilarte TR: Dysregulation of BDNF-TrkB signaling in developing hippocampal neurons by Pb(2+): implications for an environmental basis of neurodevelopmental disorders. *Toxicol Sci* 2012, 127(1):277-295.

6. Zheng W: Toxicology of choroid plexus: special reference to metal-induced neurotoxicities. *Microsc Res Tech* 2001, 52(1):89-103.

7. Harry GJ, Schmitt TJ, Gong Z, Brown H, Zawia N, Evans HL: Lead-induced alterations of glial fibrillary acidic protein (GFAP) in the developing rat brain. *Toxicol Appl Pharmacol* 1996, 139(1):84-93.

8. Hogberg HT, Kinsner-Ovaskainen A, Coecke S, Hartung T, Bal-Price AK: mRNA expression is a relevant tool to identify developmental neurotoxicants using an in vitro approach. *Toxicol Sci* 2010, 113(1):95-115.

9. Rai A, Maurya SK, Khare P, Srivastava A, Bandyopadhyay S: Characterization of developmental neurotoxicity of As, Cd, and Pb mixture: synergistic action of metal mixture in glial and neuronal functions. *Toxicol Sci* 2010, 118(2):586-601.

10. Posser T, de Aguiar CB, Garcez RC, Rossi FM, Oliveira CS, Trentin AG, Neto VM, Leal RB: Exposure of C6 glioma cells to Pb(II) increases the phosphorylation of p38(MAPK) and JNK1/2 but not of ERK1/2. *Arch Toxicol* 2007, 81(6):407-414.

11. Marchetti C: Molecular targets of lead in brain neurotoxicity. *Neurotox Res* 2003, 5(3):221-236.

12. Ahmad F, Salahuddin M, Alamoudi W, Acharya S: Dysfunction of cortical synapse-specific mitochondria in developing rats exposed to lead and its amelioration by ascorbate supplementation. *Neuropsychiatr Dis Treat* 2018, 14:813-824.

13. Geier DA, Kern JK, King PG, Sykes LK, Geier MR: Hair toxic metal concentrations and autism spectrum disorder severity in young children. *Int J Environ Res Public Health* 2012, 9(12):4486-4497.

14. Marlowe M, Cossairt A, Moon C, Errera J, MacNeel A, Peak R, Ray J, Schroeder C: Main and interaction effects of metallic toxins on classroom behavior. *J Abnorm Child Psychol* 1985, 13(2):185-198.

15. Marlowe M, Stellern J, Errera J, Moon C: Main and interaction effects of metal pollutants on visual-motor performance. *Arch Environ Health* 1985, 40(4):221-225.

16. Moon C, Marlowe M, Stellern J, Errera J: Main and interaction effects of metallic pollutants on cognitive functioning. *J Learn Disabil* 1985, 18(4):217-221.

17. Obrenovich ME, Shamberger RJ, Lonsdale D: Altered heavy metals and transketolase found in autistic spectrum disorder. *Biol Trace Elem Res* 2011, 144(1-3):475-486.

18. Thatcher RW, Lester ML, McAlaster R, Horst R: Effects of low levels of cadmium and lead on cognitive functioning in children. *Arch Environ Health* 1982, 37(3):159-166.

19. Adams JB, Baral M, Geis E, Mitchell J, Ingram J, Hensley A, Zappia I, Newmark S, Gehn E, Rubin RA *et al*: The severity of autism is associated with toxic metal body burden and red blood cell glutathione levels. *J Toxicol* 2009, 2009:532640.

20. Lin CC, Chen YC, Su FC, Lin CM, Liao HF, Hwang YH, Hsieh WS, Jeng SF, Su YN, Chen PC: In utero exposure to environmental lead and manganese and neurodevelopment at 2 years of age. *Environ Res* 2013, 123:52-57.

21. Parajuli RP, Fujiwara T, Umezaki M, Watanabe C: Association of cord blood levels of lead, arsenic, and zinc with neurodevelopmental indicators in newborns: a birth cohort study in Chitwan Valley, Nepal. *Environ Res* 2013, 121:45-51.

22. Rauh VA, Perera FP, Horton MK, Whyatt RM, Bansal R, Hao X, Liu J, Barr DB, Slotkin TA, Peterson BS: Brain anomalies in children exposed prenatally to a common organophosphate pesticide. *Proc Natl Acad Sci U S A* 2012, 109(20):7871-7876.

23. Rauh V, Arunajadai S, Horton M, Perera F, Hoepner L, Barr DB, Whyatt R: Seven-year neurodevelopmental scores and prenatal exposure to chlorpyrifos, a common agricultural pesticide. *Environ Health Perspect* 2011, 119(8):1196-1201.

24. Dereumeaux C, Saoudi A, Pecheux M, Berat B, de Crouy-Chanel P, Zaros C, Brunel S, Delamaire C, le Tertre A, Lefranc A *et al*: Biomarkers of exposure to environmental contaminants in French pregnant women from the Elfe cohort in 2011. *Environ Int* 2016, 97:56-67.

25. Calderon J, Navarro ME, Jimenez-Capdeville ME, Santos-Diaz MA, Golden A, Rodriguez-Leyva I, Borja-Aburto V, Diaz-Barriga F: Exposure to arsenic and lead and neuropsychological development in Mexican children. *Environ Res* 2001, 85(2):69-76.

26. Rosado JL, Ronquillo D, Kordas K, Rojas O, Alatorre J, Lopez P, Garcia-Vargas G, Del Carmen Caamano M, Cebrian ME, Stoltzfus RJ: Arsenic exposure and cognitive performance in Mexican schoolchildren. *Environ Health Perspect* 2007, 115(9):1371-1375.

27. Claus Henn B, Schnaas L, Ettinger AS, Schwartz J, Lamadrid-Figueroa H, Hernandez-Avila M, Amarasiriwardena C, Hu H, Bellinger DC, Wright RO *et al*: Associations of early childhood manganese and lead coexposure with neurodevelopment. *Environ Health Perspect* 2012, 120(1):126-131.

28. Kim Y, Kim BN, Hong YC, Shin MS, Yoo HJ, Kim JW, Bhang SY, Cho SC: Co-exposure to environmental lead and manganese affects the intelligence of school-aged children. *Neurotoxicology* 2009, 30(4):564-571.

29. Lucchini RG, Zoni S, Guazzetti S, Bontempi E, Micheletti S, Broberg K, Parrinello G, Smith DR: Inverse association of intellectual function with very low blood lead but not with manganese exposure in Italian adolescents. *Environ Res* 2012, 118:65-71.

30. Goodrich JM, Dolinoy DC, Sanchez BN, Zhang Z, Meeker JD, Mercado-Garcia A, Solano-Gonzalez M, Hu H, Tellez-Rojo MM, Peterson KE: Adolescent epigenetic profiles and environmental exposures from early life through peri-adolescence. *Environ Epigenet* 2016, 2(3):dvw018.

31. Kim Y, Ha EH, Park H, Ha M, Hong YC, Kim EJ, Kim BN: Prenatal lead and cadmium co-exposure and infant neurodevelopment at 6 months of age: the Mothers and Children's Environmental Health (MOCEH) study. *Neurotoxicology* 2013, 35:15-22.

32. Lee YS, Lewis JA, Ippolito DL, Hussainzada N, Lein PJ, Jackson DA, Stallings JD: Repeated exposure to neurotoxic levels of chlorpyrifos alters hippocampal expression of neurotrophins and neuropeptides. *Toxicology* 2016, 340:53-62.

33. Ozdemir S, Altun S, Ozkaraca M, Ghosi A, Toraman E, Arslan H: Cypermethrin, chlorpyrifos, deltamethrin, and imidacloprid exposure up-regulates the mRNA and protein levels of bdnf and c-fos in the brain of adult zebrafish (Danio rerio). *Chemosphere* 2018, 203:318-326.

34. Slotkin TA, Seidler FJ, Fumagalli F: Targeting of neurotrophic factors, their receptors, and signaling pathways in the developmental neurotoxicity of organophosphates in vivo and in vitro. *Brain Res Bull* 2008, 76(4):424-438.

35. Slotkin TA, Cousins MM, Tate CA, Seidler FJ: Persistent cholinergic presynaptic deficits after neonatal chlorpyrifos exposure. *Brain Res* 2001, 902(2):229-243.

36. Betancourt AM, Filipov NM, Carr RL: Alteration of neurotrophins in the hippocampus and cerebral cortex of young rats exposed to chlorpyrifos and methyl parathion. *Toxicol Sci* 2007, 100(2):445-455.

37. Schuh RA, Lein PJ, Beckles RA, Jett DA: Noncholinesterase mechanisms of chlorpyrifos neurotoxicity: altered phosphorylation of Ca2+/cAMP response element binding protein in cultured neurons. *Toxicol Appl Pharmacol* 2002, 182(2):176-185.

38. Das KP, Barone S, Jr.: Neuronal differentiation in PC12 cells is inhibited by chlorpyrifos and its metabolites: is acetylcholinesterase inhibition the site of action? *Toxicol Appl Pharmacol* 1999, 160(3):217-230.

39. Zhang J, Dai H, Deng Y, Tian J, Zhang C, Hu Z, Bing G, Zhao L: Neonatal chlorpyrifos exposure induces loss of dopaminergic neurons in young adult rats. *Toxicology* 2015, 336:17-25.

40. Yang D, Howard A, Bruun D, Ajua-Alemanj M, Pickart C, Lein PJ: Chlorpyrifos and chlorpyrifos-oxon inhibit axonal growth by interfering with the morphogenic activity of acetylcholinesterase. *Toxicol Appl Pharmacol* 2008, 228(1):32-41.

41. Howard AS, Bucelli R, Jett DA, Bruun D, Yang D, Lein PJ: Chlorpyrifos exerts opposing effects on axonal and dendritic growth in primary neuronal cultures. *Toxicol Appl Pharmacol* 2005, 207(2):112-124.

42. Middlemore-Risher ML, Adam BL, Lambert NA, Terry AV, Jr.: Effects of chlorpyrifos and chlorpyrifos-oxon on the dynamics and movement of mitochondria in rat cortical neurons. *J Pharmacol Exp Ther* 2011, 339(2):341-349.

43. Johnson FO, Chambers JE, Nail CA, Givaruangsawat S, Carr RL: Developmental chlorpyrifos and methyl parathion exposure alters radial-arm maze performance in juvenile and adult rats. *Toxicol Sci* 2009, 109(1):132-142.

44. Grandjean P, Landrigan PJ: Neurobehavioural effects of developmental toxicity. *Lancet Neurol* 2014, 13(3):330-338.

45. Schlumpf M, Kypke K, Wittassek M, Angerer J, Mascher H, Mascher D, Vokt C, Birchler M, Lichtensteiger W: Exposure patterns of UV filters, fragrances, parabens, phthalates, organochlor pesticides, PBDEs, and PCBs in human milk: correlation of UV filters with use of cosmetics. *Chemosphere* 2010, 81(10):1171-1183.

46. Hartle JC, Cohen RS, Sakamoto P, Barr DB, Carmichael SL: Chemical Contaminants in Raw and Pasteurized Human Milk. *J Hum Lact* 2018, 34(2):340-349.

47. Galea KS, MacCalman L, Jones K, Cocker J, Teedon P, Cherrie JW, van Tongeren M: Urinary biomarker concentrations of captan, chlormequat, chlorpyrifos and cypermethrin in UK adults and children living near agricultural land. *J Expo Sci Environ Epidemiol* 2015, 25(6):623-631.

48. Heffernan AL, English K, Toms L, Calafat AM, Valentin-Blasini L, Hobson P, Broomhall S, Ware RS, Jagals P, Sly PD *et al*: Cross-sectional biomonitoring study of pesticide exposures in Queensland, Australia, using pooled urine samples. *Environ Sci Pollut Res Int* 2016, 23(23):23436-23448.

49. Babina K, Dollard M, Pilotto L, Edwards JW: Environmental exposure to organophosphorus and pyrethroid pesticides in South Australian preschool children: a cross sectional study. *Environ Int* 2012, 48:109-120.

50. Roca M, Miralles-Marco A, Ferre J, Perez R, Yusa V: Biomonitoring exposure assessment to contemporary pesticides in a school children population of Spain. *Environ Res* 2014, 131:77-85.

51. United States Centers for Disease Control and Prevention. Fourth national report on human exposure to environmental chemicals. [https://[www.cdc.gov/exposurereport/](http://www.cdc.gov/exposurereport/)]

52. Morgan MK, Sheldon LS, Croghan CW, Jones PA, Robertson GL, Chuang JC, Wilson NK, Lyu CW: Exposures of preschool children to chlorpyrifos and its degradation product 3,5,6-trichloro-2-pyridinol in their everyday environments. *J Expo Anal Environ Epidemiol* 2005, 15(4):297-309.

53. van Wendel de Joode B, Mora AM, Lindh CH, Hernandez-Bonilla D, Cordoba L, Wesseling C, Hoppin JA, Mergler D: Pesticide exposure and neurodevelopment in children aged 6-9 years from Talamanca, Costa Rica. *Cortex* 2016, 85:137-150.

54. Rauh VA, Garfinkel R, Perera FP, Andrews HF, Hoepner L, Barr DB, Whitehead R, Tang D, Whyatt RW: Impact of prenatal chlorpyrifos exposure on neurodevelopment in the first 3 years of life among inner-city children. *Pediatrics* 2006, 118(6):e1845-1859.

55. Silver MK, Shao J, Zhu B, Chen M, Xia Y, Kaciroti N, Lozoff B, Meeker JD: Prenatal naled and chlorpyrifos exposure is associated with deficits in infant motor function in a cohort of Chinese infants. *Environ Int* 2017, 106:248-256.

56. Whyatt RM, Garfinkel R, Hoepner LA, Andrews H, Holmes D, Williams MK, Reyes A, Diaz D, Perera FP, Camann DE *et al*: A biomarker validation study of prenatal chlorpyrifos exposure within an inner-city cohort during pregnancy. *Environ Health Perspect* 2009, 117(4):559-567.

57. Cheong A, Johnson SA, Howald EC, Ellersieck MR, Camacho L, Lewis SM, Vanlandingham MM, Ying J, Ho SM, Rosenfeld CS: Gene expression and DNA methylation changes in the hypothalamus and hippocampus of adult rats developmentally exposed to bisphenol A or ethinyl estradiol: a CLARITY-BPA consortium study. *Epigenetics* 2018, 13(7):704-720.

58. Kundakovic M, Gudsnuk K, Herbstman JB, Tang D, Perera FP, Champagne FA: DNA methylation of BDNF as a biomarker of early-life adversity. *Proc Natl Acad Sci U S A* 2015, 112(22):6807-6813.

59. Wang IJ, Chen CY, Bornehag CG: Bisphenol A exposure may increase the risk of development of atopic disorders in children. *Int J Hyg Environ Health* 2016, 219(3):311-316.

60. Wang C, Niu R, Zhu Y, Han H, Luo G, Zhou B, Wang J: Changes in memory and synaptic plasticity induced in male rats after maternal exposure to bisphenol A. *Toxicology* 2014, 322:51-60.

61. Xu X, Xie L, Hong X, Ruan Q, Lu H, Zhang Q, Zhang G, Liu X: Perinatal exposure to bisphenol-A inhibits synaptogenesis and affects the synaptic morphological development in offspring male mice. *Chemosphere* 2013, 91(8):1073-1081.

62. Warita K, Mitsuhashi T, Ohta K, Suzuki S, Hoshi N, Miki T, Takeuchi Y: In vitro evaluation of gene expression changes for gonadotropin-releasing hormone 1, brain-derived neurotrophic factor and neurotrophic tyrosine kinase, receptor, type 2, in response to bisphenol A treatment. *Congenit Anom (Kyoto)* 2013, 53(1):42-45.

63. Seki S, Aoki M, Hosokawa T, Saito T, Masuma R, Komori M, Kurasaki M: Bisphenol-A suppresses neurite extension due to inhibition of phosphorylation of mitogen-activated protein kinase in PC12 cells. *Chem Biol Interact* 2011, 194(1):23-30.

64. Jang YJ, Park HR, Kim TH, Yang WJ, Lee JJ, Choi SY, Oh SB, Lee E, Park JH, Kim HP *et al*: High dose bisphenol A impairs hippocampal neurogenesis in female mice across generations. *Toxicology* 2012, 296(1-3):73-82.

65. Imamura L, Kurashina K, Kawahira T, Omoteno M, Tsuda M: Additional repression of activity-dependent c-fos and BDNF mRNA expression by lipophilic compounds accompanying a decrease in Ca2+ influx into neurons. *Neurotoxicology* 2005, 26(1):17-25.

66. Elsworth JD, Jentsch JD, Groman SM, Roth RH, Redmond ED, Jr., Leranth C: Low circulating levels of bisphenol-A induce cognitive deficits and loss of asymmetric spine synapses in dorsolateral prefrontal cortex and hippocampus of adult male monkeys. *J Comp Neurol* 2015, 523(8):1248-1257.

67. Elsworth JD, Jentsch JD, Vandevoort CA, Roth RH, Redmond DE, Jr., Leranth C: Prenatal exposure to bisphenol A impacts midbrain dopamine neurons and hippocampal spine synapses in non-human primates. *Neurotoxicology* 2013, 35:113-120.

68. Xu X, Gu T, Shen Q: Different effects of bisphenol-A on memory behavior and synaptic modification in intact and estrogen-deprived female mice. *J Neurochem* 2015, 132(5):572-582.

69. Hajszan T, Leranth C: Bisphenol A interferes with synaptic remodeling. *Front Neuroendocrinol* 2010, 31(4):519-530.

70. Tian YH, Baek JH, Lee SY, Jang CG: Prenatal and postnatal exposure to bisphenol a induces anxiolytic behaviors and cognitive deficits in mice. *Synapse* 2010, 64(6):432-439.

71. Tiwari SK, Agarwal S, Tripathi A, Chaturvedi RK: Bisphenol-A Mediated Inhibition of Hippocampal Neurogenesis Attenuated by Curcumin via Canonical Wnt Pathway. *Mol Neurobiol* 2016, 53(5):3010-3029.

72. Hines EP, Mendola P, von Ehrenstein OS, Ye X, Calafat AM, Fenton SE: Concentrations of environmental phenols and parabens in milk, urine and serum of lactating North Carolina women. *Reprod Toxicol* 2015, 54:120-128.

73. Perez-Lobato R, Mustieles V, Calvente I, Jimenez-Diaz I, Ramos R, Caballero-Casero N, Lopez-Jimenez FJ, Rubio S, Olea N, Fernandez MF: Exposure to bisphenol A and behavior in school-age children. *Neurotoxicology* 2016, 53:12-19.

74. Lim YH, Bae S, Kim BN, Shin CH, Lee YA, Kim JI, Hong YC: Prenatal and postnatal bisphenol A exposure and social impairment in 4-year-old children. *Environ Health* 2017, 16(1):79.

75. Carlsson A, Sorensen K, Andersson AM, Frederiksen H, Juul A: Bisphenol A, phthalate metabolites and glucose homeostasis in healthy normal-weight children. *Endocr Connect* 2018, 7(1):232-238.

76. Amin MM, Ebrahim K, Hashemi M, Shoshtari-Yeganeh B, Rafiei N, Mansourian M, Kelishadi R: Association of exposure to Bisphenol A with obesity and cardiometabolic risk factors in children and adolescents. *Int J Environ Health Res* 2019, 29(1):94-106.

77. Lee S, Lee HA, Park B, Han H, Park BH, Oh SY, Hong YS, Ha EH, Park H: A prospective cohort study of the association between bisphenol A exposure and the serum levels of liver enzymes in children. *Environ Res* 2018, 161:195-201.

78. Khan A, Park H, Lee HA, Park B, Gwak HS, Lee HR, Jee SH, Park YH: Elevated Metabolites of Steroidogenesis and Amino Acid Metabolism in Preadolescent Female Children With High Urinary Bisphenol A Levels: A High-Resolution Metabolomics Study. *Toxicol Sci* 2017, 160(2):371-385.

79. Lv Y, Rui C, Dai Y, Pang Q, Li Y, Fan R, Lu S: Exposure of children to BPA through dust and the association of urinary BPA and triclosan with oxidative stress in Guangzhou, China. *Environ Sci Process Impacts* 2016, 18(12):1492-1499.

80. Vafeiadi M, Roumeliotaki T, Myridakis A, Chalkiadaki G, Fthenou E, Dermitzaki E, Karachaliou M, Sarri K, Vassilaki M, Stephanou EG *et al*: Association of early life exposure to bisphenol A with obesity and cardiometabolic traits in childhood. *Environ Res* 2016, 146:379-387.

81. Findlay LC, Kohen DE: Bisphenol A and child and youth behaviour: Canadian Health Measures Survey 2007 to 2011. *Health Rep* 2015, 26(8):3-9.

82. Choi J, Eom J, Kim J, Lee S, Kim Y: Association between some endocrine-disrupting chemicals and childhood obesity in biological samples of young girls: a cross-sectional study. *Environ Toxicol Pharmacol* 2014, 38(1):51-57.

83. Yang F, Xu L, Zhu L, Zhang Y, Meng W, Liu R: Competitive immunoassay for analysis of bisphenol A in children's sera using a specific antibody. *Environ Sci Pollut Res Int* 2016, 23(11):10714-10721.

84. Casas M, Forns J, Martinez D, Avella-Garcia C, Valvi D, Ballesteros-Gomez A, Luque N, Rubio S, Julvez J, Sunyer J *et al*: Exposure to bisphenol A during pregnancy and child neuropsychological development in the INMA-Sabadell cohort. *Environ Res* 2015, 142:671-679.

85. Woods MM, Lanphear BP, Braun JM, McCandless LC: Gestational exposure to endocrine disrupting chemicals in relation to infant birth weight: a Bayesian analysis of the HOME Study. *Environ Health* 2017, 16(1):115.

86. Sager PR, Matheson DW: Mechanisms of neurotoxicity related to selective disruption of microtubules and intermediate filaments. *Toxicology* 1988, 49(2-3):479-492.

87. Farina M, Rocha JB, Aschner M: Mechanisms of methylmercury-induced neurotoxicity: evidence from experimental studies. *Life Sci* 2011, 89(15-16):555-563.

88. Aschner M, Syversen T: Methylmercury: recent advances in the understanding of its neurotoxicity. *Ther Drug Monit* 2005, 27(3):278-283.

89. Khan MA, Wang F: Mercury-selenium compounds and their toxicological significance: toward a molecular understanding of the mercury-selenium antagonism. *Environ Toxicol Chem* 2009, 28(8):1567-1577.

90. Ekino S, Susa M, Ninomiya T, Imamura K, Kitamura T: Minamata disease revisited: an update on the acute and chronic manifestations of methyl mercury poisoning. *J Neurol Sci* 2007, 262(1-2):131-144.

91. Harada M: Minamata disease: methylmercury poisoning in Japan caused by environmental pollution. *Crit Rev Toxicol* 1995, 25(1):1-24.

92. Grandjean P, Weihe P, White RF, Debes F, Araki S, Yokoyama K, Murata K, Sorensen N, Dahl R, Jorgensen PJ: Cognitive deficit in 7-year-old children with prenatal exposure to methylmercury. *Neurotoxicol Teratol* 1997, 19(6):417-428.

93. Unoki T, Akiyama M, Kumagai Y, Goncalves FM, Farina M, da Rocha JBT, Aschner M: Molecular Pathways Associated With Methylmercury-Induced Nrf2 Modulation. *Front Genet* 2018, 9:373.

94. Unoki T, Abiko Y, Toyama T, Uehara T, Tsuboi K, Nishida M, Kaji T, Kumagai Y: Methylmercury, an environmental electrophile capable of activation and disruption of the Akt/CREB/Bcl-2 signal transduction pathway in SH-SY5Y cells. *Sci Rep* 2016, 6:28944.

95. Fujimura M, Usuki F: Methylmercury causes neuronal cell death through the suppression of the TrkA pathway: in vitro and in vivo effects of TrkA pathway activators. *Toxicol Appl Pharmacol* 2015, 282(3):259-266.

96. Colquhoun A, Eibl JK, Krol KM, Chan HM, Ross GM: Conformational analysis of the effects of methylmercury on nerve growth factor and brain derived neurotrophic factor. *Environ Toxicol Pharmacol* 2009, 27(2):298-302.

97. Ursinyova M, Masanova V, Uhnakova I, Murinova LP, Patayova H, Rausova K, Trnovec T, Stencl J, Gajdos M: Prenatal and Early Postnatal Exposure to Total Mercury and Methylmercury from Low Maternal Fish Consumption. *Biol Trace Elem Res* 2018.

98. McDowell MA, Dillon CF, Osterloh J, Bolger PM, Pellizzari E, Fernando R, Montes de Oca R, Schober SE, Sinks T, Jones RL *et al*: Hair mercury levels in U.S. children and women of childbearing age: reference range data from NHANES 1999-2000. *Environ Health Perspect* 2004, 112(11):1165-1171.

99. Oulhote Y, Shamim Z, Kielsen K, Weihe P, Grandjean P, Ryder LP, Heilmann C: Children's white blood cell counts in relation to developmental exposures to methylmercury and persistent organic pollutants. *Reprod Toxicol* 2017, 68:207-214.

100. Weinhouse C, Ortiz EJ, Berky AJ, Bullins P, Hare-Grogg J, Rogers L, Morales AM, Hsu-Kim H, Pan WK: Hair Mercury Level is Associated with Anemia and Micronutrient Status in Children Living Near Artisanal and Small-Scale Gold Mining in the Peruvian Amazon. *Am J Trop Med Hyg* 2017, 97(6):1886-1897.

101. Tatsuta N, Murata K, Iwai-Shimada M, Yaginuma-Sakurai K, Satoh H, Nakai K: Psychomotor Ability in Children Prenatally Exposed to Methylmercury: The 18-Month Follow-Up of Tohoku Study of Child Development. *Tohoku J Exp Med* 2017, 242(1):1-8.

102. Xu Y, Wahlberg K, Love TM, Watson GE, Yeates AJ, Mulhern MS, McSorley EM, Strain JJ, Davidson PW, Shamlaye CF *et al*: Associations of blood mercury and fatty acid concentrations with blood mitochondrial DNA copy number in the Seychelles Child Development Nutrition Study. *Environ Int* 2019, 124:278-283.

103. Patel NB, Xu Y, McCandless LC, Chen A, Yolton K, Braun J, Jones RL, Dietrich KN, Lanphear BP: Very low-level prenatal mercury exposure and behaviors in children: the HOME Study. *Environ Health* 2019, 18(1):4.

104. Wahlberg K, Love TM, Pineda D, Engstrom K, Watson GE, Thurston SW, Yeates AJ, Mulhern MS, McSorley EM, Strain JJ *et al*: Maternal polymorphisms in glutathione-related genes are associated with maternal mercury concentrations and early child neurodevelopment in a population with a fish-rich diet. *Environ Int* 2018, 115:142-149.

105. Llop S, Tran V, Ballester F, Barbone F, Sofianou-Katsoulis A, Sunyer J, Engstrom K, Alhamdow A, Love TM, Watson GE *et al*: CYP3A genes and the association between prenatal methylmercury exposure and neurodevelopment. *Environ Int* 2017, 105:34-42.

106. Ripley S, Robinson E, Johnson-Down L, Andermann A, Ayotte P, Lucas M, Nieboer E: Blood and hair mercury concentrations among Cree First Nations of Eeyou Istchee (Quebec, Canada): time trends, prenatal exposure and links to local fish consumption. *Int J Circumpolar Health* 2018, 77(1):1474706.

107. You SH, Wang SL, Pan WH, Chan WC, Fan AM, Lin P: Risk assessment of methylmercury based on internal exposure and fish and seafood consumption estimates in Taiwanese children. *Int J Hyg Environ Health* 2018, 221(4):697-703.

108. Basu N, Eng JWL, Perkins M, Santa-Rios A, Martincevic G, Carlson K, Neitzel RL: Development and application of a novel method to characterize methylmercury exposure in newborns using dried blood spots. *Environ Res* 2017, 159:276-282.

109. Vejrup K, Brandlistuen RE, Brantsaeter AL, Knutsen HK, Caspersen IH, Alexander J, Lundh T, Meltzer HM, Magnus P, Haugen M: Prenatal mercury exposure, maternal seafood consumption and associations with child language at five years. *Environ Int* 2018, 110:71-79.

110. Schneider T, Przewlocki R: Behavioral alterations in rats prenatally exposed to valproic acid: animal model of autism. *Neuropsychopharmacology* 2005, 30(1):80-89.

111. Verrotti A, Scaparrotta A, Cofini M, Chiarelli F, Tiboni GM: Developmental neurotoxicity and anticonvulsant drugs: a possible link. *Reprod Toxicol* 2014, 48:72-80.

112. Balmer NV, Weng MK, Zimmer B, Ivanova VN, Chambers SM, Nikolaeva E, Jagtap S, Sachinidis A, Hescheler J, Waldmann T *et al*: Epigenetic changes and disturbed neural development in a human embryonic stem cell-based model relating to the fetal valproate syndrome. *Hum Mol Genet* 2012, 21(18):4104-4114.

113. Bold J, Sakata-Haga H, Fukui Y: Spinal nerve defects in mouse embryos prenatally exposed to valproic acid. *Anat Sci Int* 2018, 93(1):35-41.

114. Farber NB, Jiang XP, Heinkel C, Nemmers B: Antiepileptic drugs and agents that inhibit voltage-gated sodium channels prevent NMDA antagonist neurotoxicity. *Mol Psychiatry* 2002, 7(7):726-733.

115. Laeng P, Pitts RL, Lemire AL, Drabik CE, Weiner A, Tang H, Thyagarajan R, Mallon BS, Altar CA: The mood stabilizer valproic acid stimulates GABA neurogenesis from rat forebrain stem cells. *J Neurochem* 2004, 91(1):238-251.

116. Death AK, McGrath KC, Handelsman DJ: Valproate is an anti-androgen and anti-progestin. *Steroids* 2005, 70(14):946-953.

117. Jacobsen NW, Halling-Sorensen B, Birkved FK: Inhibition of human aromatase complex (CYP19) by antiepileptic drugs. *Toxicol In Vitro* 2008, 22(1):146-153.

118. Theunissen PT, Robinson JF, Pennings JL, de Jong E, Claessen SM, Kleinjans JC, Piersma AH: Transcriptomic concentration-response evaluation of valproic acid, cyproconazole, and hexaconazole in the neural embryonic stem cell test (ESTn). *Toxicol Sci* 2012, 125(2):430-438.

119. Tsuru N, Maeda T, Tsuruoka M: Three cases of delivery under sodium valproate--placental transfer, milk transfer and probable teratogenicity of sodium valproate. *Jpn J Psychiatry Neurol* 1988, 42(1):89-96.

120. Kacirova I, Grundmann M, Brozmanova H: Serum levels of valproic acid during delivery in mothers and in umbilical cord - correlation with birth length and weight. *Biomed Pap Med Fac Univ Palacky Olomouc Czech Repub* 2015, 159(4):569-575.

121. Bank AM, Stowe ZN, Newport DJ, Ritchie JC, Pennell PB: Placental passage of antiepileptic drugs at delivery and neonatal outcomes. *Epilepsia* 2017, 58(5):e82-e86.

122. Johannessen Landmark C, Farmen AH, Burns ML, Baftiu A, Lossius MI, Johannessen SI, Tomson T: Pharmacokinetic variability of valproate during pregnancy - Implications for the use of therapeutic drug monitoring. *Epilepsy Res* 2018, 141:31-37.

123. Dickerson SM, Cunningham SL, Gore AC: Prenatal PCBs disrupt early neuroendocrine development of the rat hypothalamus. *Toxicol Appl Pharmacol* 2011, 252(1):36-46.

124. Boix J, Cauli O, Felipo V: Developmental exposure to polychlorinated biphenyls 52, 138 or 180 affects differentially learning or motor coordination in adult rats. Mechanisms involved. *Neuroscience* 2010, 167(4):994-1003.

125. Vreugdenhil HJ, Mulder PG, Emmen HH, Weisglas-Kuperus N: Effects of perinatal exposure to PCBs on neuropsychological functions in the Rotterdam cohort at 9 years of age. *Neuropsychology* 2004, 18(1):185-193.

126. Boersma ER, Lanting CI: Environmental exposure to polychlorinated biphenyls (PCBs) and dioxins. Consequences for longterm neurological and cognitive development of the child lactation. *Adv Exp Med Biol* 2000, 478:271-287.

127. Eriksson P, Fredriksson A: Developmental neurotoxicity of four ortho-substituted polychlorinated biphenyls in the neonatal mouse. *Environ Toxicol Pharmacol* 1996, 1(3):155-165.

128. Winneke G, Bucholski A, Heinzow B, Kramer U, Schmidt E, Walkowiak J, Wiener JA, Steingruber HJ: Developmental neurotoxicity of polychlorinated biphenyls (PCBS): cognitive and psychomotor functions in 7-month old children. *Toxicol Lett* 1998, 102-103:423-428.

129. Winneke G, Kramer U, Sucker K, Walkowiak J, Fastabend A, Heinzow B, Steingruber HJ: PCB-related neurodevelopmental deficit may be transient: follow-up of a cohort at 6 years of age. *Environ Toxicol Pharmacol* 2005, 19(3):701-706.

130. Walkowiak J, Wiener JA, Fastabend A, Heinzow B, Kramer U, Schmidt E, Steingruber HJ, Wundram S, Winneke G: Environmental exposure to polychlorinated biphenyls and quality of the home environment: effects on psychodevelopment in early childhood. *Lancet* 2001, 358(9293):1602-1607.

131. Forns J, Torrent M, Garcia-Esteban R, Grellier J, Gascon M, Julvez J, Guxens M, Grimalt JO, Sunyer J: Prenatal exposure to polychlorinated biphenyls and child neuropsychological development in 4-year-olds: an analysis per congener and specific cognitive domain. *Sci Total Environ* 2012, 432:338-343.

132. Ruel MVM, Bos AF, Soechitram SD, Meijer L, Sauer PJJ, Berghuis SA: Prenatal exposure to organohalogen compounds and children's mental and motor development at 18 and 30 months of age. *Neurotoxicology* 2019, 72:6-14.

133. El Majidi N, Bouchard M, Carrier G: Systematic analysis of the relationship between standardized prenatal exposure to polychlorinated biphenyls and mental and motor development during follow-up of nine children cohorts. *Regul Toxicol Pharmacol* 2013, 66(1):130-146.
