# Supplementary material for: Assessment of developmental neurotoxicity induced by chemical mixtures using an adverse outcome pathway concept
Source: Environ Health. 2020 Feb 24;19:23. doi: 10.1186/s12940-020-00578-x (PMC7038628; doi:10.1186/s12940-020-00578-x)

**Figure S1: Effects elicited by Bisphenol A (BPA)**

**
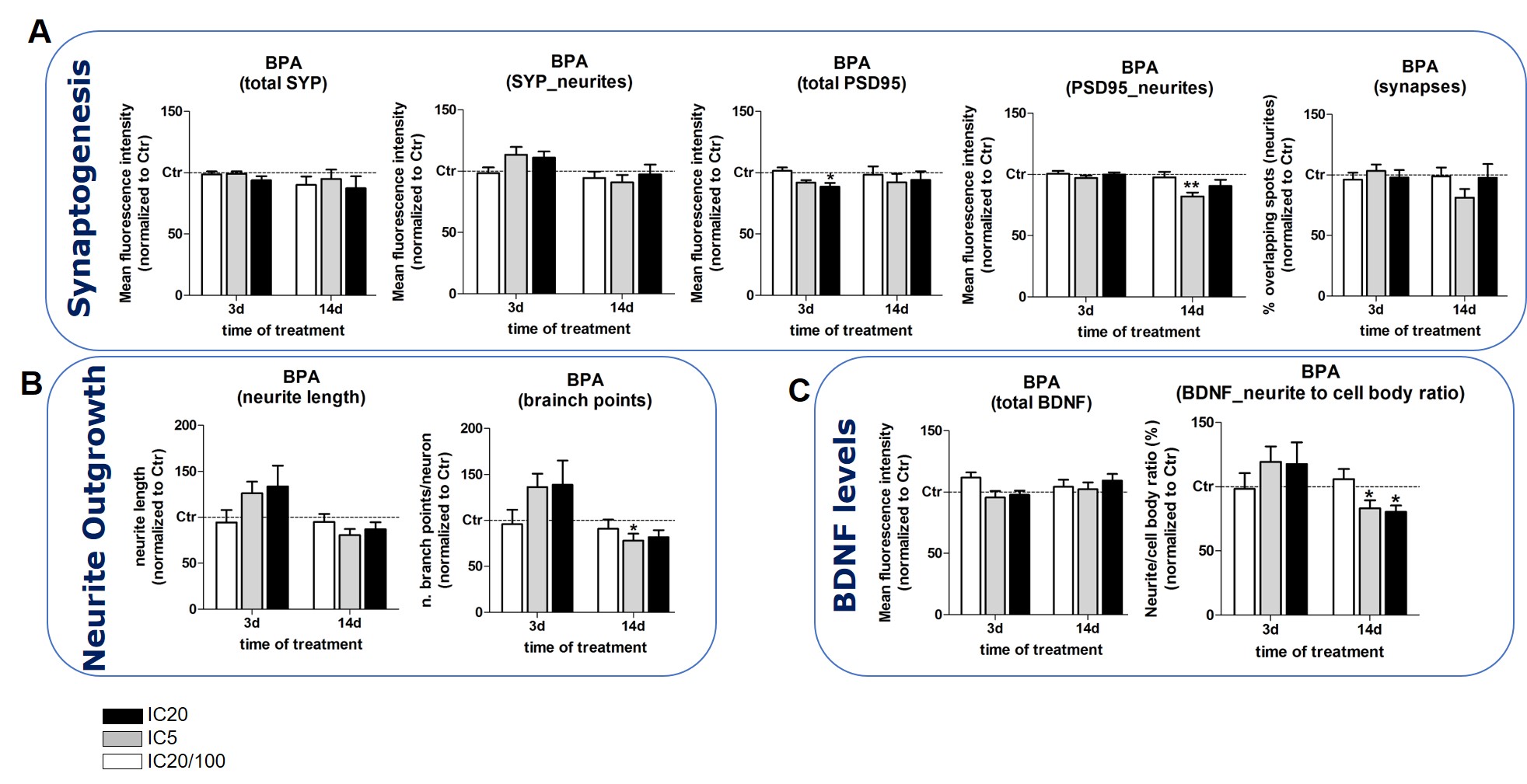
**

**Figure S2: Effects elicited by Chlorpyrifos (CPF)**

**
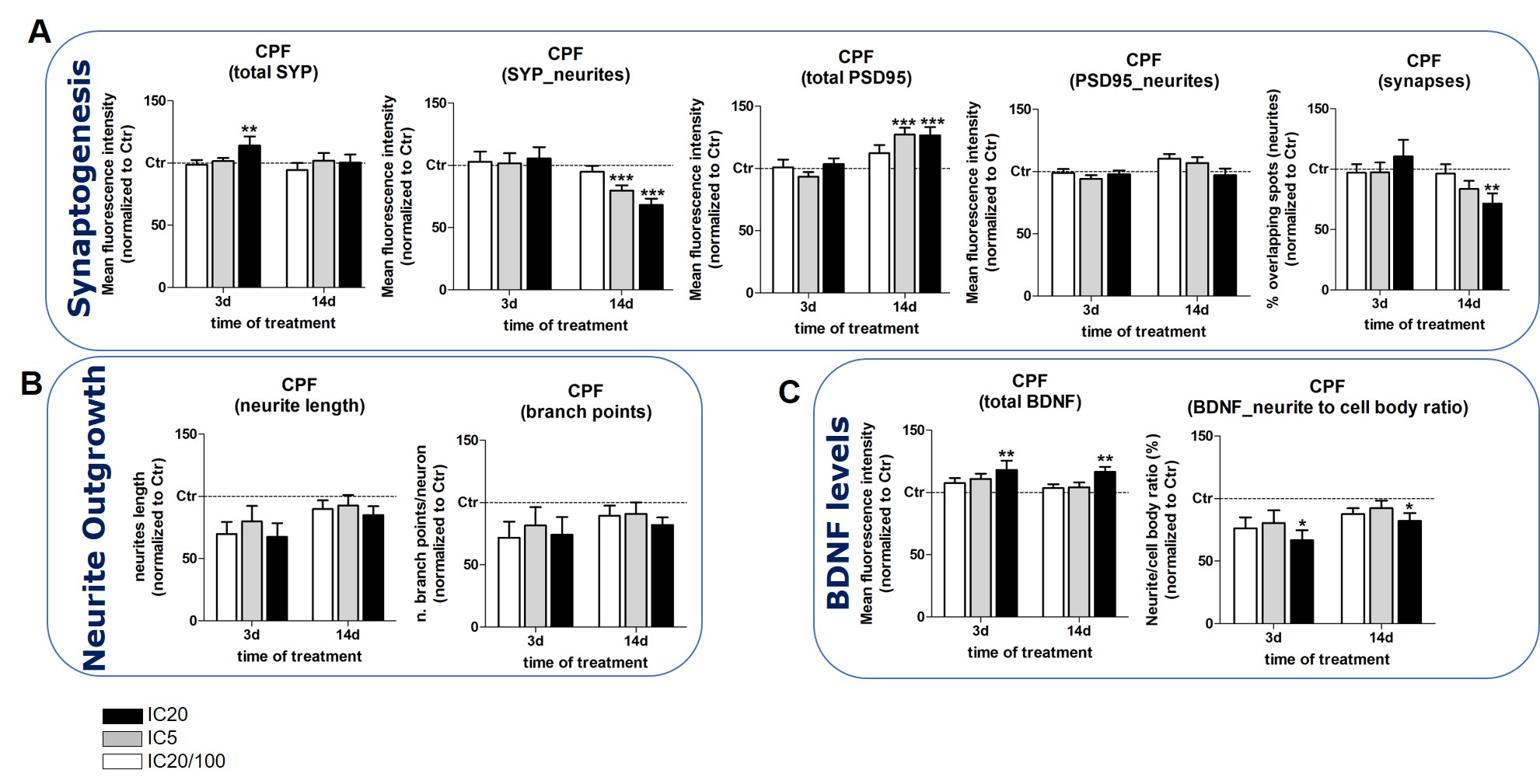
**

**Figure S3: Effects elicited by Lead(II) chloride (Lead)**


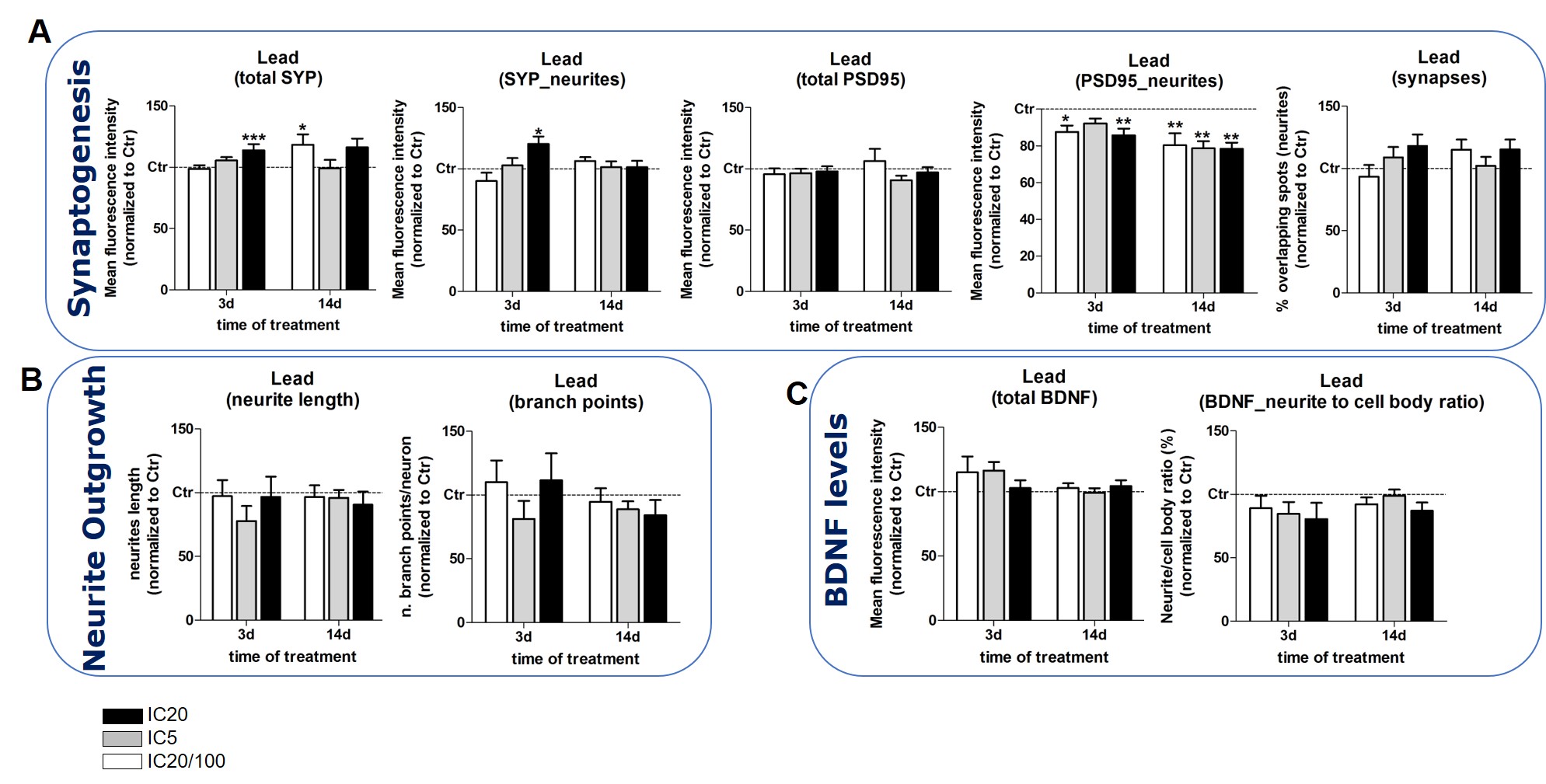


**Figure S4: Effects elicited by Methylmercury(II) chloride (Methyl-Hg)**


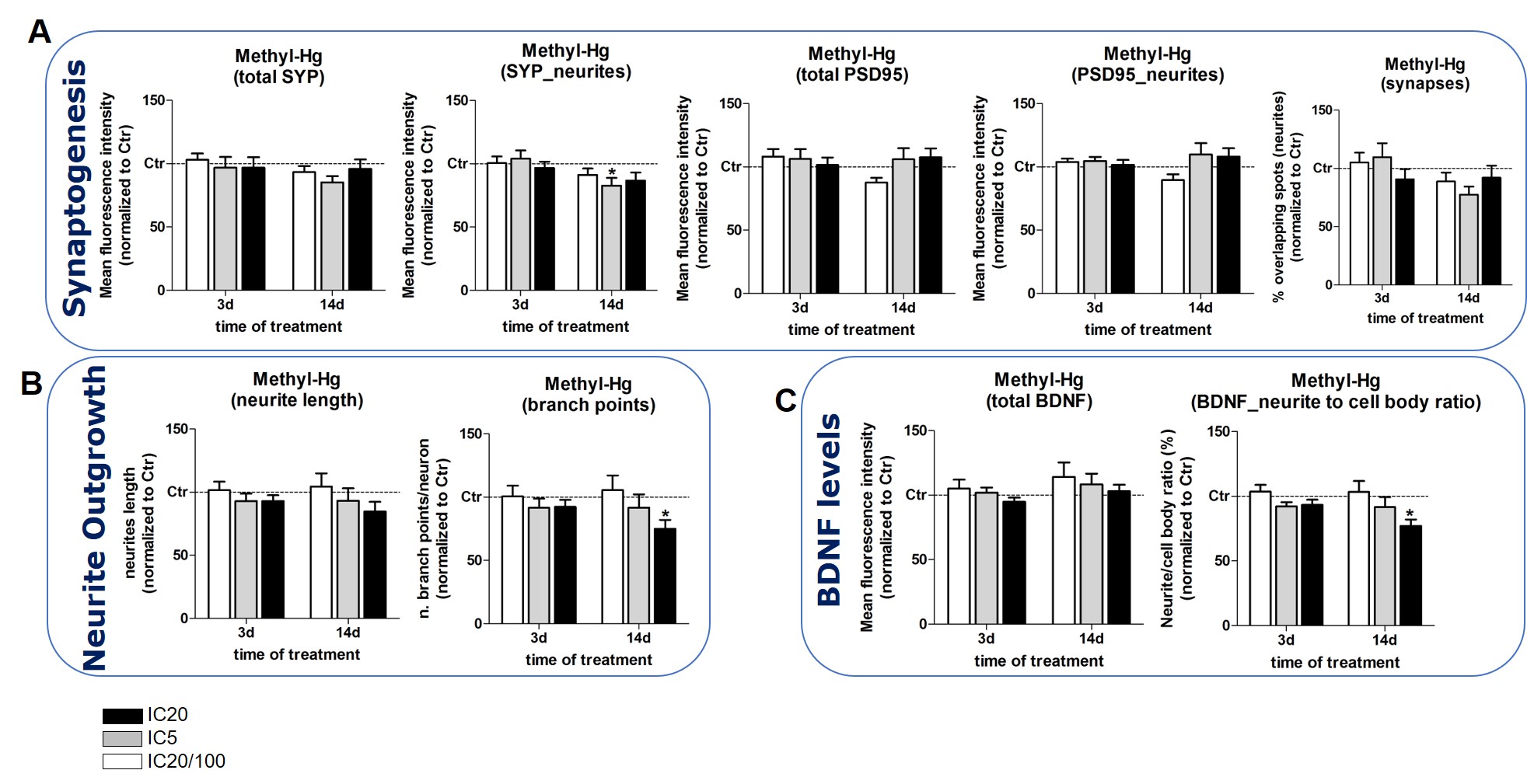


**Figure S5: Effects elicited by PCB138**


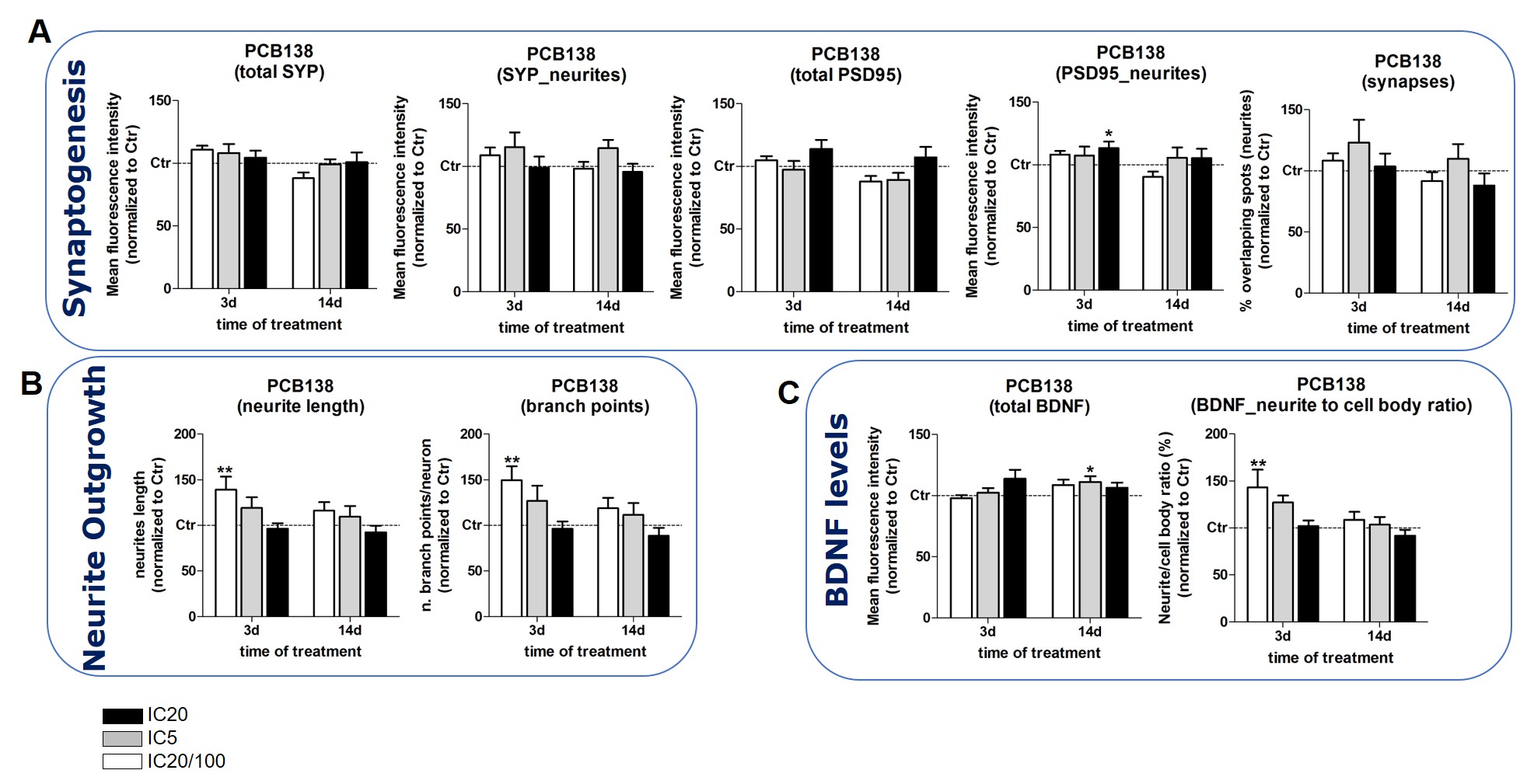


**Figure S6: Effects elicited by Valproic acid (VA)**


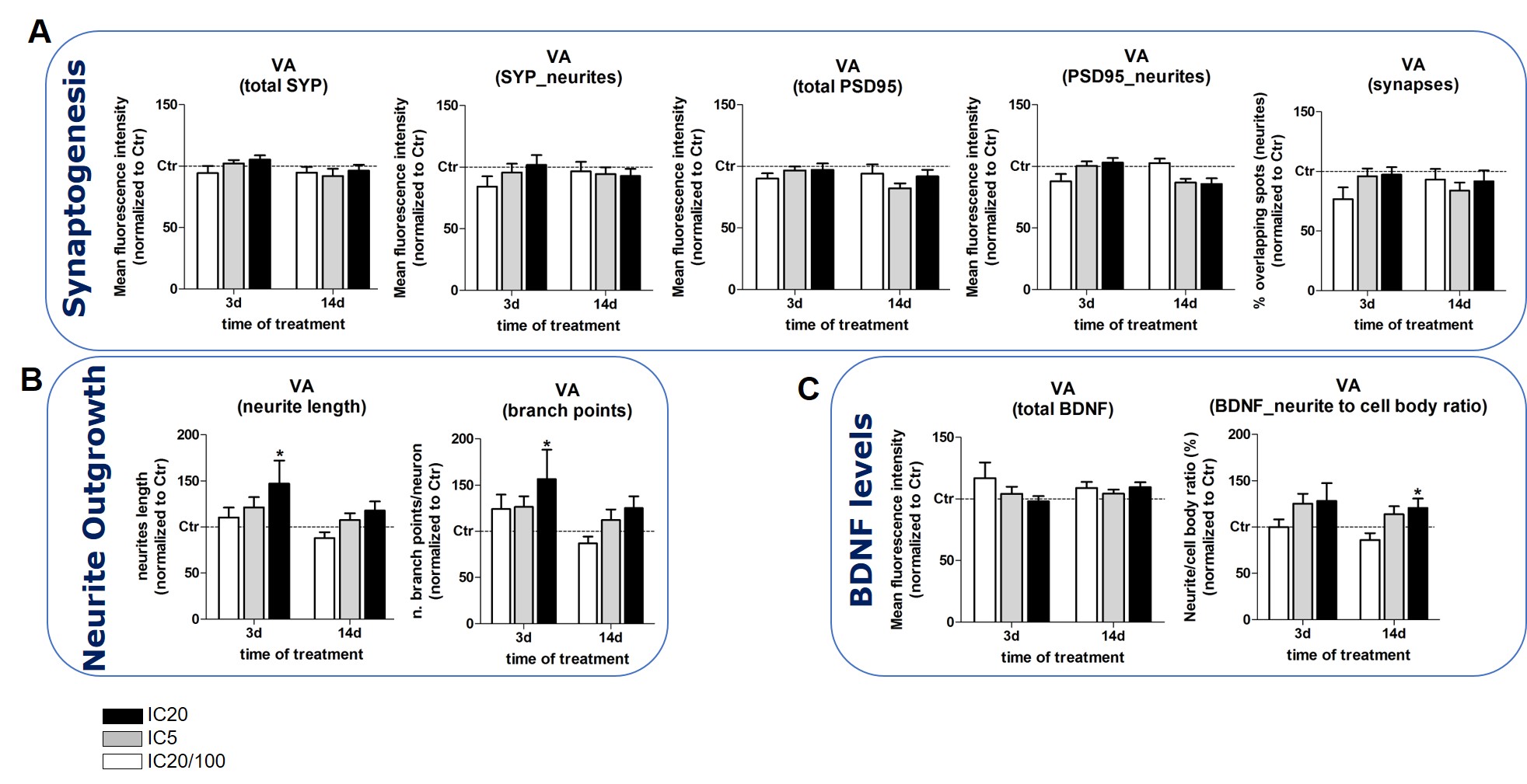

Supplement: Supplementary file 2 — Additional file 2: Figure S1. Effects elicited by Bisphenol A (BPA); quantitative evaluation of immunocytochemistry using HCI (Cellomics platform). IMR90-derived NSCs were differentiated for 7 DIV and treated for either 3 or 14 days with three different concentrations of BPA (0.29 μM, IC20/100, white bars; 12.74 μM, IC5, grey bars; 28.96 μM, IC20, black bars) in comparison to solvent control (0.1% DMSO, Ctr) at the respective time point. Analysis of BPA effects on: (A) total (i.e., cell body and neurites) and in neurites only expression of SYP (pre-synaptic) and PSD95 (post-synaptic proteins (MAP2 staining was used as a marker of neurites), and the number of overlapping SYP/PSD95 spots (synapses) in the neurites; (B) neurite length and branch points per neurite; (C) total BDNF protein levels and BDNF expression ratio, comparing neurite to cell body. Data are represented as mean ± S.E.M. of 3–4 biological replicates. Figure S2. Effects elicited by Chlorpyrifos (CPF); quantitative evaluation of immunocytochemistry using HCI (Cellomics platform). IMR90-derived NSCs were differentiated for 7 DIV and treated for either 3 or 14 days with three different concentrations of CPF (0.37 μM, IC20/100, white bars; 21.01 μM, IC5, grey bars; 37.10 μM, IC20, black bars) in comparison to solvent control (0.1% DMSO, Ctr) at the respective time point. Analysis of CPF effects on: (A) total (i.e., cell body and neurites) and in neurites only expression of SYP (pre-synaptic) and PSD95 (post-synaptic proteins (MAP2 staining was used as a marker of neurites), and the number of overlapping SYP/PSD95 spots (synapses) in the neurites; (B) neurite length and branch points per neurite; (C) total BDNF protein levels and BDNF expression ratio, comparing neurite to cell body. Data are represented as mean ± S.E.M. of 3–4 biological replicates. Figure S3. Effects elicited by Lead(II) chloride (Lead); quantitative evaluation of immunocytochemistry using HCI (Cellomics platform). IMR90-derived NSCs wer [file 12940_2020_578_MOESM2_ESM.docx]
