# Supplementary material for: Assessment of developmental neurotoxicity induced by chemical mixtures using an adverse outcome pathway concept
Source: Environ Health. 2020 Feb 24;19:23. doi: 10.1186/s12940-020-00578-x (PMC7038628; doi:10.1186/s12940-020-00578-x)
Supplement: Supplementary file 3 — Additional file 3: Figure S7. Single chemicals and mixtures effects on synaptogenesis. HiPSC-derived NSCs were differentiated for 7 DIV, and then treated for either 3 days (A-C) or 14 days (D-F) with single chemicals (BPA, CPF, Lead, Methyl-Hg, PCB138 and VA) or three types of mixtures: (i) a mixture with the 3 similar MoA chemicals (‘3-Sim’), (ii) a mixture with the 3 dissimilar MoA chemicals (‘3-Diss’), and (iii) a mixture with all 6 chemicals (‘All’). Graphs show: (A, D) total PSD95 protein levels; (B, E) total SYP protein levels, and (C, F) the number or overlapping SYP and PSD95 spots at the levels of nerites (i.e., SYP/PSD95 co-localization, synapses). All samples were normalized to solvent control (0.1% DMSO, Ctr) at the respective time point. LOAEC-syn (red curves) and their serial dilutions (respectively black (1:2) and blue (1:4) curves) were tested as indicated in Table 3 (reporting concentrations tested for each individual chemical). Data are represented as mean ± S.E.M. of 3–4 biological replicates. Figure S8. Single chemicals and mixtures effects on neurite outgrowth. HiPSC-derived NSCs were differentiated for 7 DIV, and then treated for either 3 days (A-D) or 14 days (E-G) with single chemicals (BPA, CPF, Lead, Methyl-Hg, PCB138 and VA) or three types of mixtures: (i) a mixture with the 3 similar MoA chemicals (‘3-Sim’), (ii) a mixture with the 3 dissimilar MoA chemicals (‘3-Diss’), and (iii) a mixture with all 6 chemicals (‘All’). Graphs show: (A, E) neurite length, (B, F) the number of branch points per neurite, (C, G) the number of neurites per neuron, and (D, H) the percentage of β-III-Tubulin+ cells. All samples were normalized to solvent control (0.1% DMSO, Ctr) at the respective time point. LOAEC-neu (red curves) and their serial dilutions (respectively black (1:2), blue (1:4) and light blue (1:8) curves) were tested as indicated in Table 3 (reporting concentrations tested for each individual chemical). Data are represented as mean ± S.E.M. of [file 12940_2020_578_MOESM3_ESM.docx]

**Figure S7: Single chemicals and mixtures effects on synaptogenesis**

**
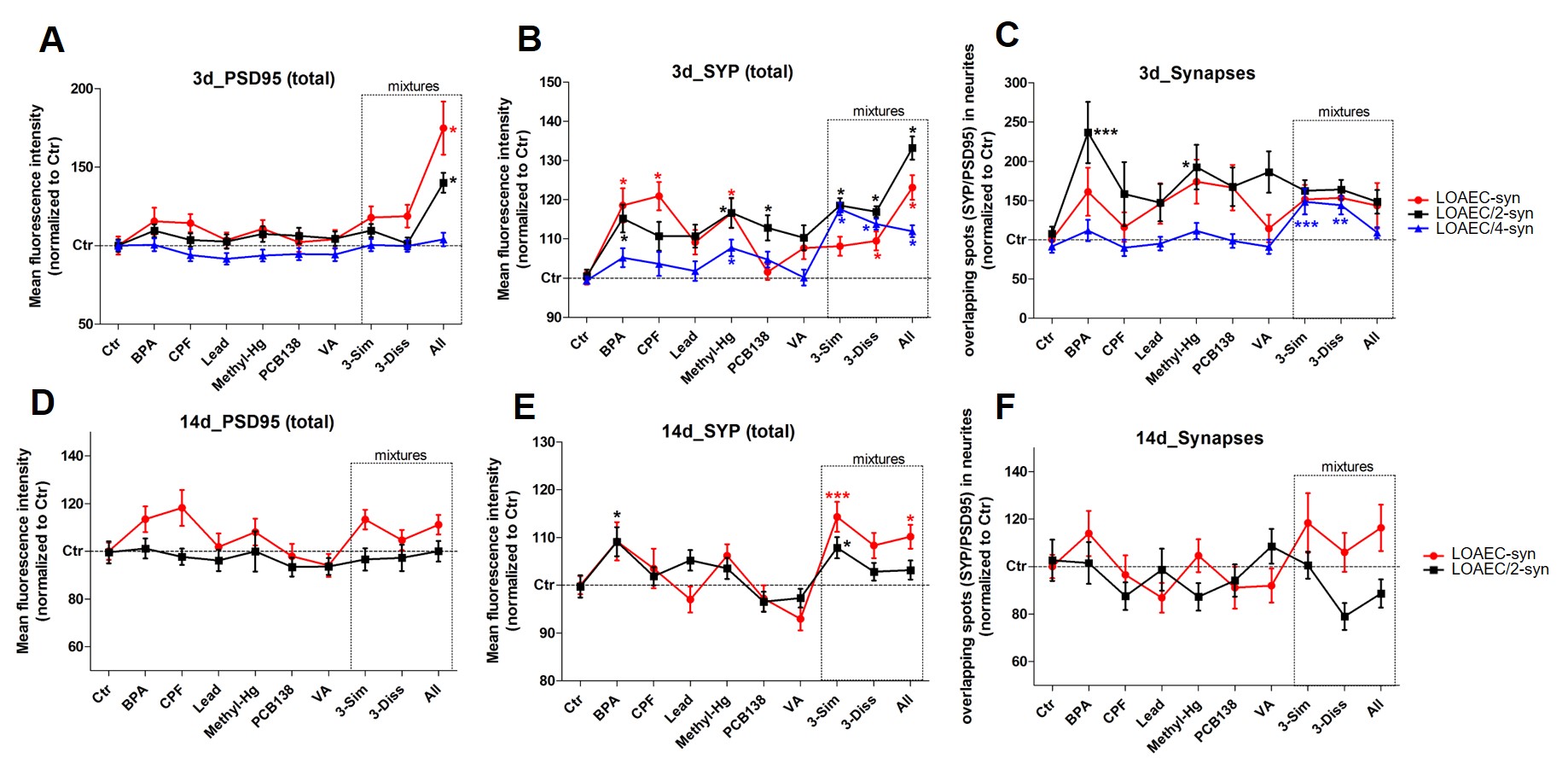
**

**Figure S8: Single chemicals and mixtures effects on neurite outgrowth**

**
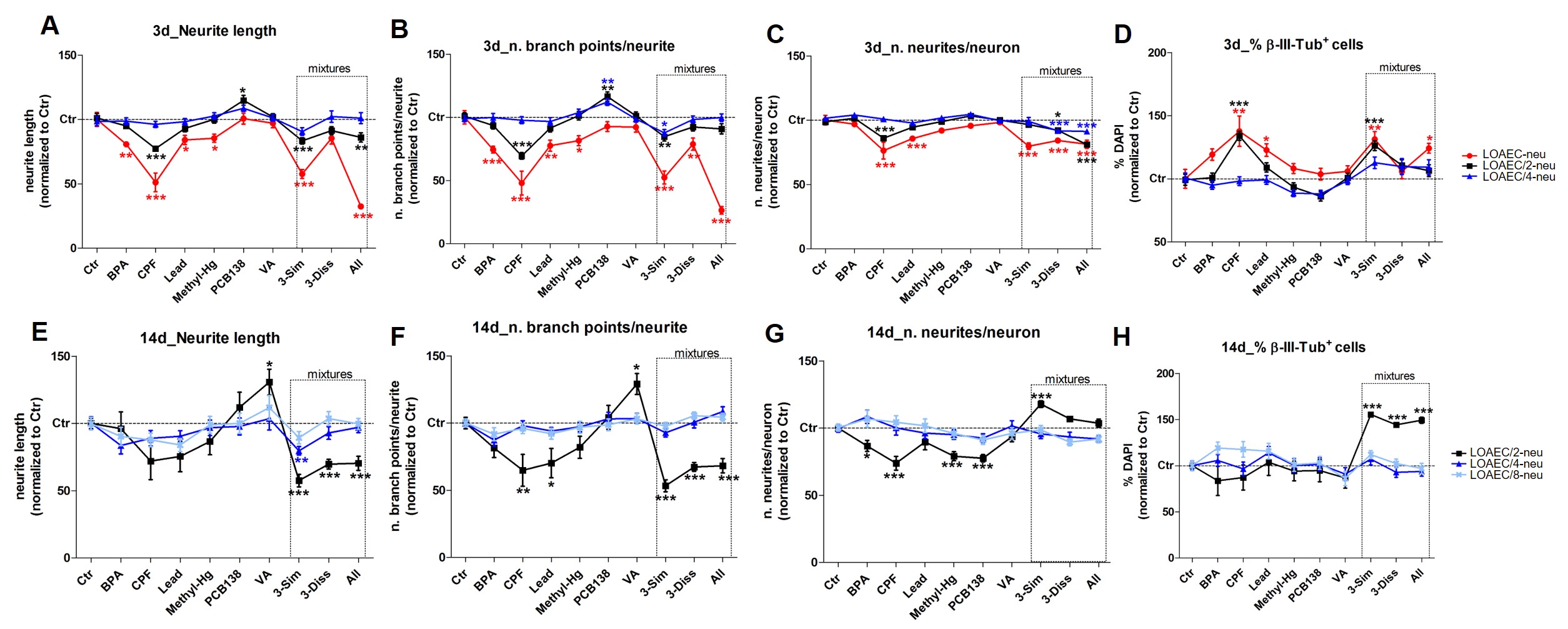
**

**Figure S9: Effects of chlorpyrifos (CPF) and valproic acid (VA) withdrawal from mixtures on neurite outgrowth**

**
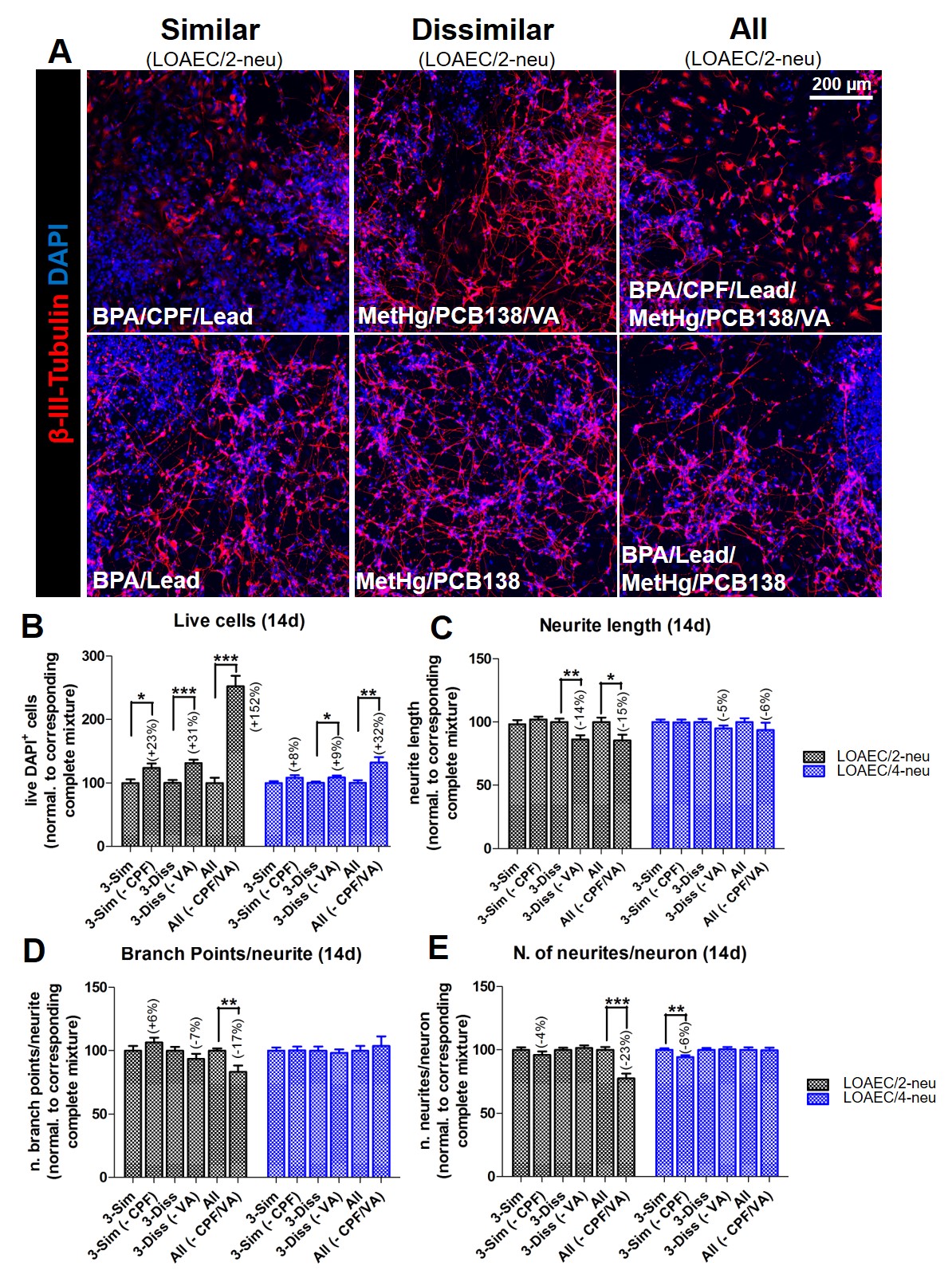
**
