# Supplementary material for: Assessment of developmental neurotoxicity induced by chemical mixtures using an adverse outcome pathway concept
Source: Environ Health. 2020 Feb 24;19:23. doi: 10.1186/s12940-020-00578-x (PMC7038628; doi:10.1186/s12940-020-00578-x)

**Figure S10: Single chemical curves**


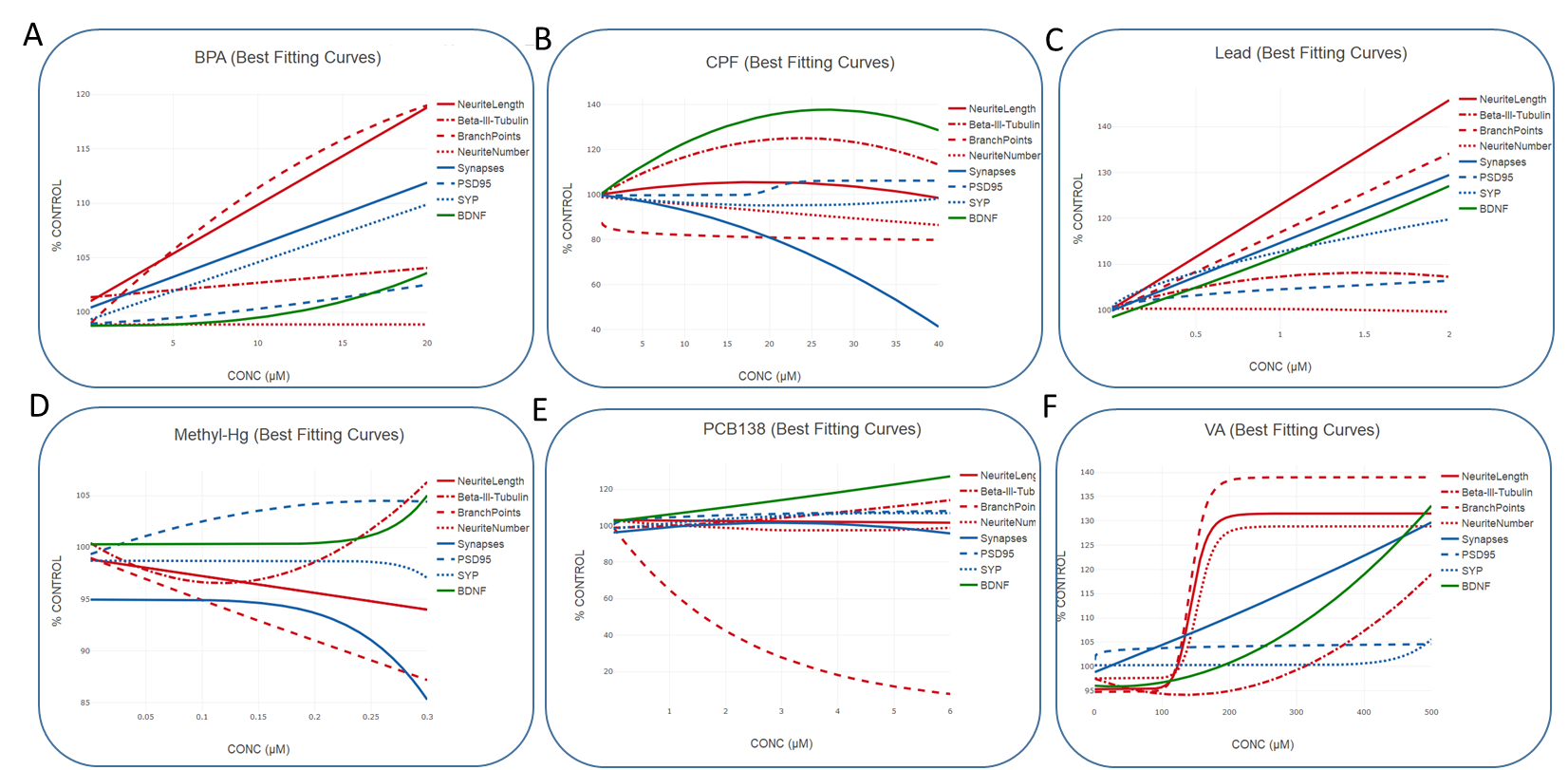


**Table S3: BMDs for synaptogenesis**

**
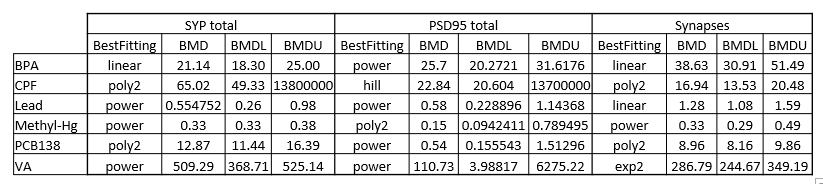
**

**Table S4: BMDs for β-III-tubulin^+^ cell percentage and neurite outgrowth**

**
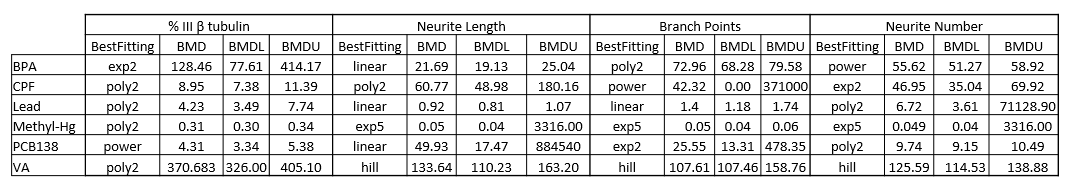
**

**Table S5: BMDs for BDNF levels**


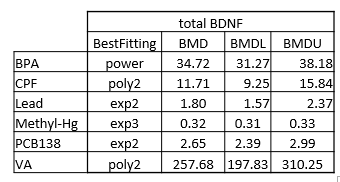

Supplement: Supplementary file 4 — Additional file 4: Figure S10. Single chemical curves. Each panel represents the modeled response of single chemicals (i.e., A, BPA; B, CPF; C, Lead; D, Methyl-Hg; E, PCB138, and F, VA) for all the selected DNT endpoints. To calculate the single chemical dose response effects for all the selected DNT endpoints the following concentrations were tested: BPA (8.5, 12.7, 19.1, 28.7, 43.0, 60.2 μM), CPF (18.5, 21.2, 24.4, 28.1, 32.3, 37.1 μM), Lead (0.001, 0.007, 0.037, 0.18, 0.91, 1.27, 1.46, 1.68, 1.93, 2.22, 2.67 μM), Methyl-Hg (0.03, 0.05, 0.09, 0.16, 0.29, 0.33 μM), PCB138 (0.01, 0.06, 0.25, 1.05, 4.39, 9.41 μM), and VA (0.5, 2.1, 8.4, 33.6, 134.4, 537 μM). The effect was estimated according to seven different mathematical models (i.e., Hill, Power, Linear, Polynomial 2, Exponential 2, Exponential 3, Exponential 4, and Exponential 5) by using the BMDExpress.2 open access software (https://github.com/auerbachs/BMDExpress-2/wiki). The best-fitting curve across the range of concentration tested is represented in the figure as percentage of response compared to the solvent control (0.1% DMSO). Table S3. BMDs synaptogenesis. The table lists the best fitting model selected for the analysis of synaptogenesis performed by BMDExpress, according to the lowest Akaike information criterion and higher fit P value. The value of the calculated BMD5 for each chemical is also reported, including the respective BMDL and BMDU. Table S4. BMDs % neurons and neurite outgrowth. The table lists the best fitting models selected for the analysis of neuronal cell (β-III-tubulin+) percentage and neurite outgrowth-related parameters performed by BMDExpress, according to the lowest Akaike information criterion and higher fit P value. The value of the calculated BMD5 for each chemical is also reported, including the respective BMDL and BMDU. Table S5. BMDs BNDF levels. The table lists the best fitting models selected for the analysis of BDNF levels performed by BMDExpress, according to the lowest [file 12940_2020_578_MOESM4_ESM.docx]
